# Oxidative stress and inflammation in neurodegenerative disorders

**DOI:** 10.1007/s00204-026-04518-5

**Published:** 2026-08-11

**Authors:** Klaudia Jomova, Suliman Y. Alomar, Richard Valko, Eugenie Nepovimova, Kamil Kuca, Marian Valko

**Affiliations:** 1 Department of Chemistry, Faculty of Natural Sciences and Informatics, Constantine the Philosopher University in Nitra, Nitra, 949 74 Slovakia; 2 Doping Research Chair, Zoology Department, College of Science, King Saud University, Riyadh, 11451 Saudi Arabia; 3 Department of Chemistry, Faculty of Sciences, University of Hradec Kralove, Hradec Kralove, 50003 Czech Republic; 4 Center of Advanced Innovation Technologies, VSB-Technical University of Ostrava, Ostrava-Poruba, 708 00 Czech Republic; 5 Biomedical Research Center, University Hospital Hradec Kralove, Hradec Kralove, 5005 Czech Republic; 6 Centre for Basic and Applied Research, Faculty of Informatics and Management, University of Hradec Kralove, Hradec Kralove, 50003 Czech Republic; 7 Faculty of Chemical and Food Technology, Slovak University of Technology, 812 37, Bratislava, Slovakia

**Keywords:** Neurodegenerative disorders, Oxidative stress, Redox metals, Antioxidant therapy

## Abstract

The brain’s consumption of approximately 20% of the body’s oxygen contributes to oxidative stress, a significant pathological factor in neurodegenerative diseases such as Alzheimer’s disease, Parkinson’s disease, Huntington’s disease, and amyotrophic lateral sclerosis. This oxidative stress, linked to low levels of antioxidant enzymes, drives neuronal death by facilitating membrane peroxidation of fatty acids, proteins, and DNA. Alzheimer’s disease is characterized by amyloid-beta (Aβ) plaque accumulation and hyperphosphorylated tau aggregates, both of which interact with mitochondria to generate reactive oxygen species (ROS). Aβ peptides bind metals such as iron and copper, catalyzing the formation of damaging hydroxyl radicals. Peripheral markers of oxidative damage, such as elevated malondialdehyde and protein carbonyls, are correlated with these processes in affected patients. In Parkinson’s disease, the loss of dopaminergic neurons in the substantia nigra is associated with pathological iron accumulation and mitochondrial complex I dysfunction, which are worsened by misfolded α-synuclein and mutations in antioxidant genes such as PINK1 and Parkin. The autooxidation of dopamine also drives oxidative stress through the generation of hydrogen peroxide and reactive quinones. Huntington’s disease involves the degeneration of medium spiny neurons in the striatum due to a polyglutamine repeat expansion in the huntingtin gene, which disrupts mitochondrial function and downregulates antioxidants, leading to excitotoxicity and ROS spikes. Amyotrophic lateral sclerosis primarily affects motor neurons due to the mutations in SOD1, which result in the production of aggregates that impair mitochondria and generate reactive nitrogen species (RNS), such as peroxynitrite. Mitigating oxidative stress in neurodegenerative disorders presents a considerable translational challenge. While low-molecular-weight antioxidant therapies for neurodegenerative disorders have shown promising results in preclinical and animal studies because they mitigate oxidative stress, their clinical efficacy is hampered by low bioavailability and difficulty in penetrating the blood‒brain barrier. To overcome these limitations, current medical research is focused on alternative delivery systems. Innovations such as nanoparticle-based drug delivery are being actively studied to help transport low-molecular-weight antioxidants across the blood‒brain barrier more safely and effectively. Several promising epidemiological trials linked high dietary intake of vitamins C and E to a reduced risk of Parkinson’s disease, and plant-derived antioxidants such as polyphenols were explored for their ability to combat neuroinflammation and reduce cognitive decline. Refined oxidative stress-suppressing strategies involve the (ii) application of mitochondrial-targeted agents to preserve ATP production; (ii) boosting the Nrf2 pathway may trigger a cascade of detoxifying enzymes; (iii) supplementation with polyphenols such as quercetin, resveratrol, and curcumin can suppress oxidative stress and dampen microglial activation (neuroinflammation); (iv) and the use of substances affecting the bidirectional network linking oxidative stress and autophagy can clear ROS-generating components. Despite some promising epidemiological data, translating oral or systemic antioxidant therapy into effective clinical treatments for humans requires further effort. A survey of current knowledge of oxidative stress and antioxidant therapy in neurodegenerative diseases is the main subject of this review.

## Introduction

Neurodegenerative diseases are a category of neurological disorders characterized by the gradual and permanent degradation of neurons within the brain and spinal cord. These diagnoses are the second leading cause of death and a primary factor of disability (Houldsworth 2024; Feigin et al. 2020). The most common neurodegenerative disorders are Alzheimer’s disease, Parkinson’s disease, Huntington’s disease (AD, PD, HD), and amyotrophic lateral sclerosis (ALS). Neurological conditions include multiple sclerosis, stroke, muscular dystrophy, and certain neuropsychiatric disorders (Soto and Pritzkow 2018). Common examples of brain conditions are epilepsy and stroke, and spine conditions are represented by spinal cord injury.

Neurodegenerative diseases are intricate and multifactorial, lacking a singular cause, and their etiology is very complex. The interplay of genetic susceptibility, environmental factors, lifestyle, and the aging process contributes to their onset (Teleanu et al. 2022). In addition, oxidative stress, which is associated with the dysregulation of metal ions and other phenomena, is a common denominator across many neurological diseases. A variety of biomarkers substantiates multiple confirmed signs, and the oxidative stress component is a common denominator of neurodegenerative disorders ( Ciurea et al. 2023) and neurological conditions.

The vulnerability of the brain to oxidative stress is determined primarily by elevated metabolic activity and oxygen consumption. High oxygen consumption and limited regenerative capacity are the main causes of oxidative damage in the brain, contributing to the increased oxidative stress component of neurodegenerative disease pathology (Olufunmilayo et al. 2023). An excess of ROS, accompanied by impaired antioxidant systems, can shift the redox balance, as evidenced by iron-mediated lipid peroxidation and protein and DNA damage, resulting in cellular dysfunction and neuronal death. However, the precise mechanisms by which cellular and molecular processes contribute to the progression of these diseases remain ambiguous. Furthermore, the absence of reliable and sensitive biomarkers complicates the potential for early diagnosis in most of these conditions. A critical issue lies in the design of suitable vectors capable of overcoming the brain–blood barrier (BBB) (Kadry et al. 2020).

Detailed insight into the role of oxidative stress in these disorders is essential for formulating potential therapies aimed at targeting reactive oxygen species (ROS) and reactive nitrogen species (RNS). Increased ROS/RNS are associated with mitochondrial dysfunction and neuroinflammation, resulting in energy deficits, disturbed redox and nonredox metal homeostasis, and protein aggregation into toxic clumps that disrupt brain cell function and contribute to the progression of neurodegenerative diseases (Murphy 2009; Guo et al. 2013).

Deeper insight into the intricate relationship between oxidative stress and neurological disorders may result in the development of therapeutic strategies, ultimately improving the quality of life for individuals impacted by these conditions. One avenue involves the use of antioxidants that may protect against oxidative stress, potentially preventing or mitigating the progression of neurological diseases.

This review examines the current understanding of the extensive effects of oxidative stress on the central nervous system (CNS) and its impact on neurodegenerative pathologies (Sies et al. 2017). The relationships among oxidative stress, neuroinflammation, mitochondrial dysfunction, and neuroprotection provided by the antioxidant network will also be the subject of discussion.

### ROS-induced oxidative stress and neurodegeneration

The vast majority of ROS acting in biological systems are derived from paramagnetic (di)oxygen molecules, which feature two parallel unpaired electrons located on antibonding π* orbitals (Jomova et al. 2023). Molecular oxygen is an oxidizing agent that can accept electrons from surrounding substances, thereby oxidizing them. Electron transfer reactions are essential for critical processes, such as the oxidative burst in macrophages, the mitochondrial electron transport chain involving ATP production via oxidative phosphorylation, the microsomal electron transport chain responsible for the detoxification of various compounds, and other processes. All these and many other processes involve radical species derived from oxygen and nitrogen as key chemical constituents in electron transfer reactions.

Physiological levels of ROS and RNS play crucial regulatory and mediating roles in cellular metabolism. An uncontrolled increase in ROS/RNS concentrations results in a prooxidant state, ultimately disrupting cellular function through extensive damage to key biomolecules. This state is termed oxidative stress and is defined as a condition arising from an imbalance between the production of ROS/RNS and the body’s ability to counteract them through the antioxidant network (Sies 1985). This phenomenon is attributed to damage to cellular components, including lipids, proteins, carbohydrates, and nucleic acids (Sies 2020). Therefore, a suitable antioxidant defense mechanism is essential for maintaining the concentration of these reactive species within physiological limits. In addition to the endogenous mechanisms that contribute to oxidative stress, external factors such as xenobiotics, ionizing radiation, viral and bacterial infections, unhealthy diets, alcohol intake, and smoking also represent a significant source of ROS (Forman and Zhang 2021).

### Superoxide radical anion and hydrogen peroxide

The main radical generated by the mitochondria is the superoxide radical anion (O_2_^•−^). The antioxidant enzyme superoxide dismutase (SOD) transforms superoxide radicals into hydrogen peroxide (H_2_O_2_) according to the following reaction: 2O_2_^•−^ + 2 H^+^ → H_2_O_2_ + O_2_. These processes occur within four membrane-associated, multisubunit protein complexes I–IV, known as the respiratory chain or electron transport chain (ETC), located in the inner mitochondrial membrane (Li et al. 2013). These complexes accept and sequentially transfer electrons, followed by proton pumping from the matrix into the intermembrane space of the mitochondria, which is essential for ATP production by oxidative phosphorylation (Olufunmilayo et al. 2023). Complex IV, also known as cytochrome c oxidase, facilitates the final step in the ETC by donating electrons to O_2_, followed by four-electron reduction, forming H_2_O_2_ molecules. During cellular respiration, electrons may leak from chain complexes (mainly I–III) and react with molecular oxygen, forming superoxide radical anions (O_2_ + 1e^−^ → O_2_^•−^) and subsequently H_2_O_2_. Concentrations of these species above physiological levels can be harmful to cells and may contribute to irreversible neurodegenerative damage.

Xanthine oxidase and NADPH oxidase are both sources of ROS, but they play distinct roles. Xanthine oxidase is an enzyme involved in the metabolism of purines and generates O_2_^•−^ and H_2_O_2_ during the conversion of hypoxanthine to xanthine and consequently to uric acid (Lim et al. 2023). NADPH oxidase, on the other hand, is a membrane-bound enzyme complex that produces O_2_^•−^ by electron transfer from NADPH across the membrane to oxygen as part of the immune response and other cellular processes. Both enzymes can contribute to oxidative stress and inflammation.

Peroxidases are a family of enzymes that use H₂O₂ to oxidize various substrates. However, peroxidases can also play a role in the generation of O_2_^•−^ via several mechanisms, such as H₂O₂−dependent or H₂O₂−independent mechanisms with the possible involvement of ferrous ions (Jomova et al. 2024).

Superoxide radicals are involved in the functioning of the innate immune system, signaling in vasodilation, the regulation of gene expression, senescence and apoptosis, inflammatory processes, and cellular differentiation. The pathological roles of O_2_^•−^ involve the production of damaging peroxynitrite (O_2_^•−^ + NO^•^→ ONOO^−^), membrane damage, altered signal transduction pathways, and impaired DNA repair mechanisms (Sánchez et al. 2025).

Hydrogen peroxide has dual functions in biological systems, acting as both a signaling molecule and a potentially harmful species (Sies 2024). The cells maintain a fragile equilibrium of H_2_O_2_. Physiological levels of H_2_O_2_ prevent damage, while controlled production and degradation enable H_2_O_2_ to function as a signaling molecule. H_2_O_2_ is involved in the regulation of various cellular activities, including cell growth, proliferation, and inflammatory responses. H_2_O_2_ plays a role in wound healing, antibacterial defense, stem cell proliferation, and adaptive responses in astrocytes that lead to neuronal protection (Sies 2017). Excessive production of H_2_O_2_ can induce oxidative stress, resulting in damage to cellular proteins, lipids, and DNA. At elevated concentrations, H_2_O_2_ can damage cell membranes and initiate apoptosis through mitochondrial pathways. Abnormal H_2_O_2_ production is associated with neurodegenerative diseases and other chronic disorders.

The cellular management of O_2_^•−^ and H_2_O_2_ is tightly associated with antioxidant enzymes. Cells employ enzymes such as superoxide dismutases (SODs) to convert O_2_^•−^ to H_2_O_2_ and catalase and peroxidase to decompose H_2_O_2_ into water and oxygen, thereby reducing its harmful effects (Zheng et al. 2023). Cells have established complex redox signaling pathways to regulate H_2_O_2_ levels and harness H_2_O_2_ for specific functions.

### Hydroxyl radicals

Hydroxyl radicals (^•^OH) are extremely reactive entities capable of inflicting considerable damage to DNA. Hydroxyl radicals are formed through the Fenton reaction, which involves the decomposition of hydrogen peroxide catalyzed by ferric ions (Xiao et al. 2024)

$${\mathrm{F}\mathrm{e}}^{2+}+{\mathrm{H}}_{2}{\mathrm{O}}_{2}\:\to\:\:\:{\mathrm{F}\mathrm{e}}^{3+}+{}^\cdot{}\mathrm{O}\mathrm{H}+{\mathrm{O}\mathrm{H}}^{-}$$.

Other sources of hydroxyl radical formation involve photolysis, decomposition of hydroperoxides (ROOH), and electrochemical processes. Hydroxyl radicals interact with DNA components (bases and the sugar‒phosphate backbone) at or close to diffusion-controlled rates, resulting in multiple forms of DNA damage, such as base alterations, strand breaks, and cross-linking (Dizdaroglu et al. 2017). This type of damage may lead to genomic instability and is associated with a range of diseases, including neurodegenerative diseases and cancer. Hydroxyl radicals can also modify proteins by oxidizing various amino acid side chains and potentially the protein backbone and trigger the lipid peroxidation process by abstracting a hydrogen atom from a polyunsaturated fatty acid (PUFA), leaving behind a lipid radical.

### Reactive nitrogen species

Reactive nitrogen species (RNS) are a group of nitrogen-derived molecules that are usually highly reactive and perform various functions within biological systems. This group includes molecules such as nitric oxide (NO^•^), nitrogen dioxide (NO_2_), and peroxynitrite (ONOO^−^), which participate in both physiological signaling and pathological mechanisms (Wang et al. 2024).

The family of enzymes, nitric oxide synthases (NOSs), consists of three isoforms (neuronal, inducible, and endothelial) and is responsible for the synthesis of NO^•^, which utilizes the amino acid L-arginine. NO^•^ is synthesized by a variety of cells throughout the body, with a significant site of production being the inner lining of blood vessels, the endothelium (Knowles and Moncada 1994). Additionally, it is generated by other cell types, such as neurons and macrophages. Although NO^•^ is synthesized mainly by nitric oxide synthase (NOS) enzymes located in the cytoplasm, it can also be produced within mitochondria, subsequently affecting mitochondrial functionality.

Neuronal nitric oxide synthase (nNOS) is an isoform responsible for the production of NO^•^ within the nervous system. NO^•^ serves as a signaling molecule that plays a crucial role in numerous physiological processes, such as synaptic plasticity, vasodilation, and neurotransmission. In addition to managing cerebral blood circulation, NO^•^ plays a crucial role in processes related to memory and learning (Yang et al. 2025).

The pathological roles of NO^•^ include nitrosylation of proteins and inhibition of platelet aggregation. Excessive production of NO^•^, especially during inflammatory processes, may play a significant role in the onset and progression of atherosclerosis, hypertension, and cardiovascular disease. In neurodegenerative disorders, elevated levels of NO^•^ can lead to neuronal injury and cell death and intensify inflammatory reactions. Abnormally high levels of NO^•^ can significantly decrease blood pressure.

NO^•^ reacts very rapidly with O_2_^•−^ to produce peroxynitrite (ONOO^−^) (Han et al. 2025).

$$\:{\mathrm{N}\mathrm{O}}^{\cdot}+\:{\mathrm{O}}_{2}^{\cdot-}\:\:\rightarrow{\mathrm{O}\mathrm{N}\mathrm{O}\mathrm{O}}^{-}\:\:$$ (k ~ 10^9^ – 10^10^ M^−1^s^− 1^).

ONOO^−^ does not contain an unpaired electron and, therefore, is not a radical; however, its ability to damage biological molecules is greater than that of many other “true” radical species. Peroxynitrite is stable in alkaline solutions, with a pKa of 6.8. However, upon protonation, it undergoes rapid decomposition, exhibiting a half-life of less than several seconds at physiological pH and temperature (Khan et al. 2000). This decomposition, depending on the conditions, results in the formation of reactive species such as hydroxyl radicals (^•^OH), nitrite (NO_2_^−^), nitrogen dioxide (NO_2_), and carbonate radical anions (CO_3_^•−^) that can readily interact with all important biomolecules, including membrane lipids, proteins, and DNA. Although ONOO^−^ is frequently associated with detrimental effects in disease, it also has several physiological functions. These functions include the modulation of cellular signaling, especially via tyrosine nitration, and the regulation of vascular tone and blood flow.

### Antioxidant protection against oxidative stress in neurodegenerative diseases

Cells have developed various protective mechanisms to mitigate the effects of oxidative stress. These include enhancing both enzymatic and nonenzymatic (small molecular weight) antioxidant defenses, chelating redox-active metal ions, and suppressing the production of reactive oxygen species (ROS) (Jomova et al. 2024).

While synthetic antioxidants are compounds with diverse chemical structures created and tested by the pharmaceutical industry for the benefit of humans, natural antioxidants are found in nature in living organisms, such as microorganisms, fungi, plants, and animals, frequently for their own benefit (Stoia et al. 2022).

The efficiency of antioxidants is mediated by the mechanism of action, polarity, site of action, stability, regeneration capacity, molecular size, and other criteria. Depending on their mechanism of action, studies categorize antioxidants into two groups: (i) primary and secondary antioxidants and (ii) catalytic (enzymatic) antioxidants.

### Primary and secondary low-molecular-weight antioxidants

Primary and secondary antioxidants differ in their mechanism of action and role in preventing oxidation. Primary antioxidants, such as vitamins C and E, flavonoids, and some synthetic compounds, directly neutralize free radicals by donating hydrogen atoms (H-atom transfer or HAT) to them, thereby neutralizing the free radicals and disrupting the oxidation chain reaction (Jomova et al. 2024). Secondary antioxidants, such as citric acid and EDTA, act by delaying oxidation through other indirect mechanisms, such as metal chelation, peroxide decomposition, or oxygen scavenging. Metal chelation by antioxidants usually suppresses their catalytic action in various radical-mediated reactions, such as the Fenton reaction, resulting in the controlled decomposition of peroxides. Certain antioxidants, such as carotenoids, have the ability to physically neutralize singlet oxygen (^1^O_2_) by quenching it, primarily through a physical process of energy transfer but also through chemical reactions. This process excites the antioxidant (e.g., the carotenoid molecule) to an excited state, which subsequently dissipates the energy as heat, thereby restoring the antioxidant to its original ground state (Losada-Barreiro et al. 2022).

### Vitamin C

Ascorbate is a water-soluble antioxidant with the ability to scavenge radicals. Ascorbate is taken up by cells through two sodium-dependent vitamin C cotransporters (Zhitkovich 2020). These transporters play crucial roles in controlling the uptake of vitamin C from the plasma into various tissues. While the plasma concentration of vitamin C is approximately 50 µM, the levels in most tissues are significantly higher, reaching typically 1–5 mM. The concentration of vitamin C in neurons exceeds 10 mM (Higdon et al. 2023).

Ascorbate scavenges superoxide radicals more than 100 times more efficiently than does glutathione (3 × 10^5^ M^−1^s^− 1^ compared with 1 × 10^3^ M^−1^s^− 1^) (Zhitkovich 2020). Considering the efficiency of superoxide radical scavenging by superoxide dismutase (SOD), it has been estimated that its removal by vitamin C accounts for approximately 8% of the cytoplasmic content.

At physiological pH, ascorbic acid (AscH_2_) is deprotonated to form the ascorbate anion (AscH^−^). This form of ascorbate reacts with ROS and redox metals more rapidly than AscH_2_does. Superoxide (O_2_^•−^) and hydroperoxyl (HOO^•^) radicals oxidize AscH^−^ to ascorbyl radical (Asc^•−^) (Shen et al. 2021).

The half-lives of ROS in biological systems are very short; therefore, their experimental detection represents a series of technical obstacles. Given that ascorbate is prevalent in various tissues, especially in the brain, indirect insights into the transient ROS generated in biological contexts can be gleaned from the qualitative and quantitative assessment of ascorbyl radical levels (Asc^•−^), which are regarded as terminal radicals in the complexity of chain radical reactions that take place in biological systems (Sharma et al. 1994; Buettner et al. 1995). Compared with the initially formed radicals in the reaction cascade, the ascorbyl radicals exhibit relatively high stability and markedly reduced reactivity. Asc^•−^ in various tissues, including the brain, can be relatively easily detected and quantified using spin trapping Electron Paramagnetic Resonance (EPR) spectroscopy (Kasparova et al. 2005).

## Vitamin E

Vitamin E is a fat-soluble vitamin found in nature and comprises eight distinct isoforms. These isoforms consist of four tocopherols (α-, β-, γ-, and δ-tocopherol) and four tocotrienols (α-, β-, γ-, and δ-tocotrienol). α- and γ-tocopherols are the most prevalent in both dietary sources and bodily tissues; the concentration of α-forms in tissues is ten times greater than that of γ-isoforms (Miyazawa et al. 2019).

The antioxidant properties of vitamin E are related to the existence of a hydroxyl group on the chromanol ring. The ROS-scavenging mechanism involves the transfer of a hydrogen atom from the hydroxyl group of the chromanol ring of vitamin E (T–OH) to the ROS (R^•^), resulting in the formation of vitamin E radicals (tocopheryl radical, T–O^•^) and a terminal, nonradical product (RH).

Vitamin E protects membranes against oxidative stress by deactivating ROS such as peroxyl (ROO^•^) and alkoxyl (RO^•^) radicals. The primary antioxidant function of vitamin E involves the conversion of lipid hydroperoxyl radicals into lipid hydroperoxides. Considering the value of the half-redox potential of vitamins C and E, vitamin C has the capacity to regenerate vitamin E from its radical tocopheryl state, thereby indirectly maintaining the integrity of cellular membranes.

It has been suggested that mitochondrial dysfunction and oxidative stress play a role in the onset and progression of neurological conditions, including Alzheimer’s disease. Patients experiencing cognitive decline exhibit reduced levels of vitamin E; the nervous system is particularly susceptible to vitamin E deficiency (Kontush and Schekatolina 2004). The administration of vitamin E (400 IU/day) over a period of one month increased vitamin E levels by more than 120% within the cerebrospinal fluid and nearly 150% in the plasma of patients with Alzheimer’s disease. Thus, vitamin E supplementation may provide some degree of protection against lipoprotein oxidation in cerebrospinal fluid.

### Flavonoids

Flavonoids constitute a vast family of polyphenolic compounds categorized into 12 primary subclasses on the basis of their chemical structure. Six subclasses of flavonoids are recognized for their dietary importance (Prochazkova et al. 2011). The absorption spectra of flavonoids exhibit two spectral bands: band I, associated with the cinnamoyl system of the B ring (300–400 nm), and band II, linked to the benzoyl system of ring A (240–285 nm). Generally, flavanones possess a saturated C ring, which may typically lead to a reduction in antioxidant activity.

The antioxidant and prooxidant properties of flavonoids are affected by the number and arrangement of hydroxyl groups. An important structural feature that influences their antioxidant ability is their low redox potential, which allows them to reduce oxidizing agents such as peroxyl and hydroxyl radicals. Their planarity and the network of conjugated π-bonds that can hold unpaired electrons also contribute to their antioxidant properties. Flavonoids mainly neutralize ROS through electron transfer or hydrogen atom transfer to hydroxyl, peroxyl, or other radical groups, creating resonance-stabilized flavonoid radicals that are less reactive (Simunkova et al. 2019). Additionally, their indirect antioxidant activity is supported by their ability to chelate redox-active metal ions, reducing their catalytic activity in radical reactions such as the Fenton reaction. The primary metal-binding sites of flavonoids include (i) the catechol moiety located in ring B, (ii) the 3-hydroxyl and 4-oxo groups found in heterocyclic ring C, and (iii) the 4-oxo and 5-hydroxy groups situated between the C and A rings. When flavonoids interact with ROS, they form flavonoid phenoxyl radicals (Fl–O^•^), which can then react with other radicals, ultimately producing the stable flavonoid quinone structure Fl–(C = O)2 (Pietta 2000a, b).

The protective effect of flavonoids against oxidative stress has primarily been documented in relation to chronic diseases associated with oxidative stress, including cardiovascular diseases, cancer, and neurological disorders. Flavonoids may enhance neuronal survival and activity in brain regions responsible for memory, such as the hippocampus (Kolibius et al. 2023). The increased activity of hippocampal neurons is related to increased production of neurotrophins, a group of enzymes that support neuronal survival. Furthermore, flavonoids safeguard dopaminergic striatal neurons from damage induced by ROS and mitigate neuroinflammation by inhibiting proinflammatory mediators (Sokolov et al. 2013). Additionally, flavonoids may promote the synthesis of nitric oxide (NO^•^), which subsequently protects endothelial functions and offers defense against the development of atherosclerotic plaques (Vauzour et al. 2008).

### Carotenoids

Carotenoids are natural pigments extensively found in the plant kingdom and are responsible for the yellow, orange, and red colors observed in numerous plants, fruits, and vegetables (Jomova and Valko 2013). Carotenoids are categorized into two primary groups: carotenes (such as beta-carotene) and xanthophylls (including lutein and zeaxanthin).

Carotenoids interact with ROS via multiple mechanisms. The initial mechanism of radical addition reactions was described more than four decades ago (Burton and Ingold 1984). This mechanism involves the incorporation of lipid peroxyl radicals (ROO•) into the polyene chain of the carotenoid (CAR), thereby generating a carbon-centered radical from the carotenoid (ROO-CAR^•^). In tissues with increased partial pressure of oxygen (such as the lungs), carotenoid carbon-centered radicals may interact with molecular oxygen, resulting in the formation of carotenoid-derived peroxyl radicals (ROO-CAR^•^ + O_2_ → ROO-CAR-OO^•^). Carotenoids can react with ROS via an electron transfer mechanism, resulting in the generation of a carotenoid cation or anion radical according to the reaction (CAR + R^•^ → CAR^•+^ + R^−^). Another mechanism involves hydrogen abstraction. This mechanism has been reported for the reaction between β-carotene and peroxyl radicals in an ethanolic environment (CAR + ROO^•^ → CAR^•^ + ROOH).

Numerous studies have suggested that dietary lutein and zeaxanthin enhance cognitive plasticity, particularly in memory (Edwards et al. 2022). These findings were further supported by a double-blind, placebo-controlled trial in which cognitively healthy women aged 60 to 80 years received supplements of lutein and zeaxanthin (12 mg and 0.5 mg, respectively) over four months (Johnson et al. 2008). Cognitive testing indicated a significant enhancement in cognitive functions among the participants in the study group. Interestingly, individuals with mild cognitive impairments presented a marked decrease in the levels of luteolin in postmortem evaluations (Johnson et al. 2013).

### Antioxidant enzymes

The radical-scavenging capacity of low-molecular-weight antioxidants has certain limitations. The additional and principal antioxidant capacity of the cell is mediated by antioxidant enzymes, representing the first line of defense against oxidative stress (Jomova et al. 2024). The concerted action of antioxidant enzymes provides a shield against the damaging effects of ROS. Antioxidant enzymes interact with intracellular ROS at a rate several thousand to millions of times greater than that of low-molecular-weight antioxidants. In addition to extracellular SOD (EC-SOD), the extracellular space is primarily protected against ROS using low-molecular-weight antioxidants. The most prominent antioxidant enzymes are superoxide dismutases (SODs), catalase (CAT), and glutathione peroxidases (GPXs).

### Superoxide dismutases

SOD is a family of the most potent cellular antioxidant enzymes and represents the primary line of cellular antioxidant defense against biological oxidants. SODs are enzymes that have an integrally coordinated metal ion in their structure. These enzymes facilitate the conversion of two molecules of superoxide radical anions (O_2_^•−^) into hydrogen peroxide and molecular oxygen (2O_2_^•−^ + 2 H^+^ $\:\underrightarrow{SOD}$ O_2_ + H_2_O_2_).

In mammals, there are three distinct types of SODs (Abreu and Cabelli 2010). Cu, Zn-SOD (SOD1) exists as a stable homodimer, with each subunit containing one copper and one zinc atom. The zinc atom has a structural role, whereas the copper atom is essential for catalytic electron transfer. SOD1 is expressed mainly in the nucleus and cytoplasm and between the inner and outer mitochondrial membranes. Mn-SOD (SOD2) is a homotetrameric enzyme that contains a manganese atom at its active site. SOD2 is located in the mitochondrial matrix, where the pH is slightly greater (approximately 7.8) than in the intermembrane space (approximately 7.0–7.4). Tetrameric Cu, Zn-SOD (SOD3) is highly prevalent in the lungs and is primarily found in the extracellular space.

The process of catalytic dismutation of the superoxide radical is quite intricate, occurs through two reactions that are nearly limited by diffusion, and has been described in detail elsewhere (Perry et al. 2010).

Altered expression and/or activity of SOD have been associated with various chronic diseases, including neurodegenerative disorders (Trist et al. 2021). The D90A mutation of SOD1 (Cu, Zn-SOD) is the most commonly observed variant and is linked to amyotrophic lateral sclerosis (ALS, referred to as Lou Gehrig’s disease), a lethal neurodegenerative disorder that impacts motor neurons. More than 100 different mutations in SOD1, resulting in changes in the amino acid sequence at a single position, have been related to familial (inherited) ALS.

### Catalase

Catalases (along with peroxidases) are oxidoreductase enzymes found in nearly all aerobic organisms. Catalase is expressed in all human organs and has the highest turnover of all the enzymes. The generally accepted mechanism of catalase activity was extensively described by Glorieux and Calderon (Glorieux and Calderon 2017) and Bauer (Bauer 2015). The majority of catalases are homotetramers that consist of four prosthetic groups, with molecular weights ranging from 200 to 340 kDa (Glorieux and Calderon 2017). There are three categories of catalases depending on their structural and functional characteristics. The first two categories include typical or true heme-containing catalases and catalase peroxidases. The third category comprises Mn-containing (nonheme) catalases. The group of typical (true) catalases is the most extensive.

These enzymes are essential for reducing oxidative stress caused by the body’s own production of hydrogen peroxide (H_2_O_2_) during aerobic metabolism. Catalase helps breakdown H_2_O_2_ by splitting the O − O bond, producing oxygen and water (2H_2_O_2_ → H_2_O + 2O_2_), which prevents the formation of reactive oxygen species (ROS) and helps maintain the cellular redox balance (Glorieux and Calderon 2017).

Changes in catalase expression contribute to a range of diseases, including neurological disorders (such as Alzheimer’s disease and Parkinson’s disease), psychiatric disorders (bipolar disorder and schizophrenia), and other chronic diseases (Glorieux and Calderon 2017).

### Glutathione peroxidase

Glutathione peroxidase (GPx) is a group of antioxidant enzymes present in all living organisms. The GPx protein family has a range of biological functions that influence numerous cellular processes (Pei et al. 2023). Eight distinct isoforms of GPx (GPx1–GPx8) have been identified in humans thus far. The most prevalent isoform is a tetrameric enzyme, glutathione peroxidase 1 (GPx1), which is composed of four identical subunits and is located in the cytoplasm of all tissues (Handy and Loscalzo 2022).

GPx facilitates the reduction of hydroperoxides (such as H_2_O_2_) to water (H_2_O) through the oxidation of reduced glutathione (GSH) to its oxidized form (GSSH) (HOOH + 2GSH $\:\underrightarrow{GPx}$ 2HOH + GSSG) and operates in conjunction with superoxide dismutase (SOD) and catalase to establish the primary defense mechanism against ROS-induced oxidative stress.

Glutathione peroxidases exhibit a nonsequential ping-pong mechanism consisting of an oxidation reaction followed by a reduction step. More details can be found elsewhere (Weaver and Skouta 2022).

**Fig. 1:**
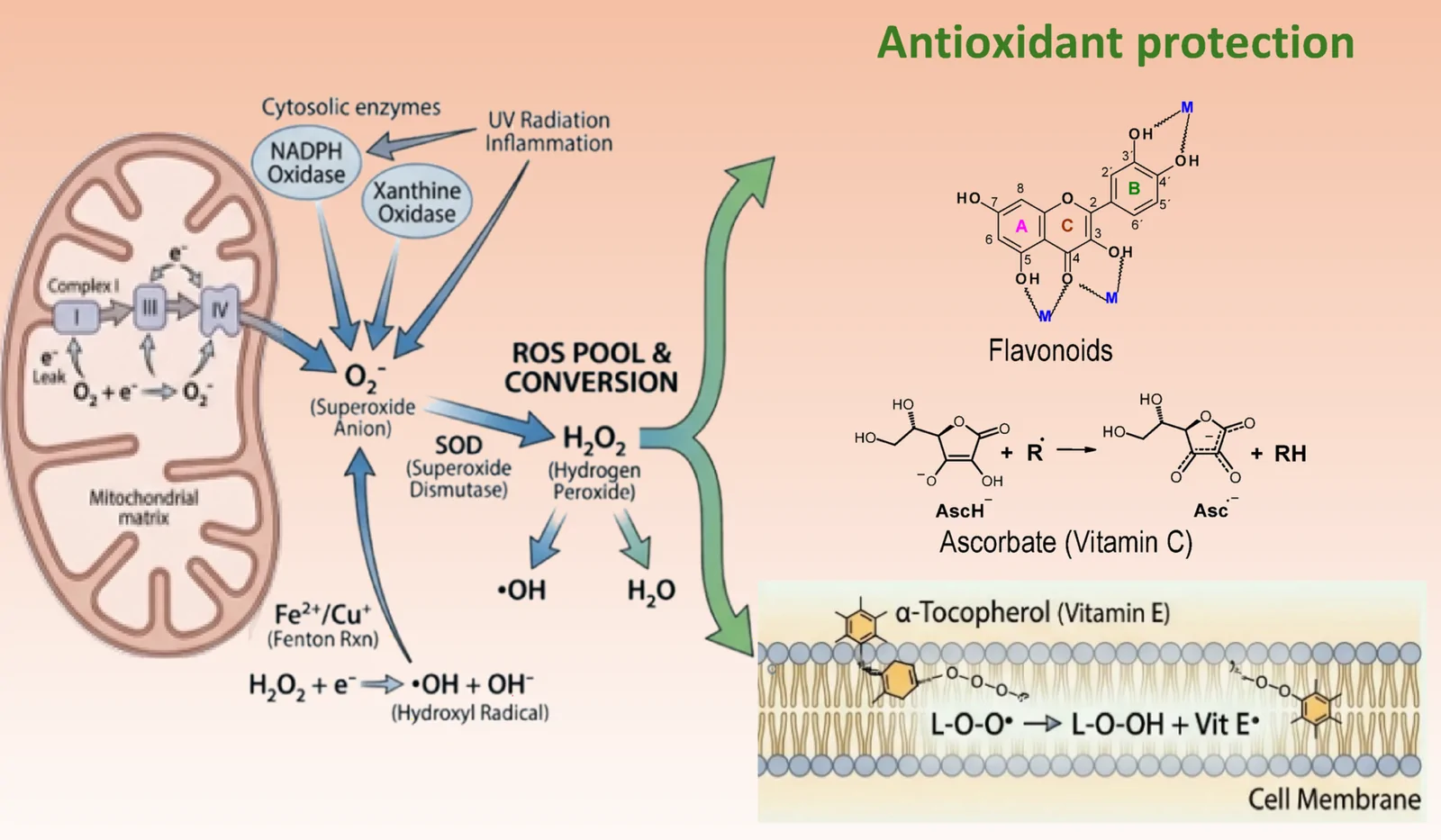
ROS formation and antioxidant protection against ROS-induced oxidative stress

GPx-mediated reduction of toxic lipid hydroperoxides (LOOHs) helps preserve the integrity, structure, and overall function of cell and neuronal membranes. Supportive antioxidant therapy may stimulate synthesis and increase GPx levels, highlighting the importance of GPx as a key antioxidant enzyme. Imperatorin, a furocoumarin and phytochemical compound, has been shown to modulate GPx in vivo, which then enhances memory in an Alzheimer’s disease mouse model (Budzynska et al. 2015). Various sources of ROS and their elimination by antioxidants are outlined in Fig. 1.

### Enzyme mimics - synthetic alternatives to natural enzymes

Antioxidant enzymes operate primarily in the intracellular environment. An effective alternative for antioxidant protection against oxidative stress in the extracellular environment can be offered by enzyme mimics. Numerous SOD mimics are bifunctional and eliminate hydrogen peroxide effectively, demonstrating catalase-like activity. Several SOD mimics can also diminish the generation of potentially harmful ONOO^−^. Among the most effective glutathione peroxidase mimics is ebselen, which is currently undergoing clinical trials for the treatment of rare inner ear disorders (Ménière’s disease) and bipolar disorder (Kil et al. 2022).

Recently, nanomaterials that exhibit enzyme-like properties, referred to as nanozymes, have garnered significant attention because of their benefits over natural enzymes (Ren et al. 2022a, b). Nanozymes display high stability under challenging conditions, adjustable physicochemical characteristics, durability, good responsiveness, and affordability. A variety of metal- and nonmetal-based nanomaterials have been evaluated for superoxide dismutase (SOD), catalase (CAT), glutathione peroxidase (GPx), and other nanomaterial-mimicking enzyme functions, with many demonstrating encouraging outcomes (Singh et al. 2023). Future investigations into nanozymes will be limited by the need for enhanced substrate specificity, regulated catalytic performance in multienzyme-like activities, and exploration of the mechanisms underlying catalytic activity.

### Key impacts of oxidative stress on the central nervous system

The central nervous system is especially susceptible to oxidative damage due to its high lipid content, particularly in neurons and their myelin-coated axons. Excessive production of ^•^OH, ONOO^−^, and other reactive species can trigger lipid peroxidation, a radical chain reaction that quickly spreads and damages many lipid molecules in cellular and organelle membranes, as well as lipoproteins (Ayala et al. 2014). This lipid peroxidation significantly contributes to neuronal damage and neurodegeneration. When ROS attack unsaturated fatty acids, they produce various breakdown products. These include lipid hydroperoxides (LOOHs), the primary products, along with secondary toxic byproducts such as malondialdehyde (MDA), 4-hydroxynonenal (HNE), acrolein, isoprostanes, and conjugated dienes (Marnett 1999). These products disrupt membrane structure and function by altering membrane fluidity, permeability, and lipid‒protein interactions, ultimately impairing ion and nutrient transport and signaling pathways, which can lead to cell death. Lipid peroxidation byproducts also interact with proteins and DNA, modifying their structure and function.

Proteins are susceptible to oxidative stress-induced conformational changes that can impair or suppress their activity (Stadtman 2004). ROS have the potential to cause alterations in their structure and functionality. ROS-induced oxidative stress may play a significant role in the misfolding and aggregation of key proteins such as tau and amyloid-beta proteins in AD, α-synuclein in Parkinson’s disease (PD), TDP-43 and SOD1 in ALS, and mutant huntingtin in Huntington disease (HD) (Koszła and Sołek 2024). The formation of these aggregates subsequently enhances ROS production and contributes to the activation of inflammatory signaling cascades, promoting chronic neuroinflammation, which exacerbates neuronal damage.

Oxidative stress-induced DNA damage is characterized by several DNA oxidation products (Dizdaroglu et al. 2022). A pivotal marker of endogenous oxidative DNA damage is a product of guanine oxidation, 8-oxo-2’-deoxyguanosine (8-OHdG) (Halliwell et al. 2021). Increased levels of 8-OHdG in bodily fluids such as urine are often used as sensitive markers of oxidative stress in an organism. Other oxidative DNA damage markers include a product of thymine oxidation, namely, thymidine glycol, an oxidized cytosine, 5-hydroxycytosine, and an oxidized form of uracil, 5-hydroxyuracil.

ROS-mediated modifications of nucleotides involve deoxyribose sugars or the phosphate backbone of DNA, such as 2’-deoxyribonolactone, 5’,8-cyclo-2’-deoxyguanosine, 5’,8-cyclo-2’-deoxyadenosine, and other products (Chatgilialoglu et al. 2011). Such modifications may have numerous cellular consequences and may interfere with standard cellular functions, which could result in the incidence of disease states or play a role in the aging process.

Advanced glycation end products (AGEs) constitute another group of compounds implicated in neurodegenerative diseases (Twarda-Clapa et al. 2022). They represent a heterogeneous and chemically varied collection of compounds that are generated either exogenously or endogenously through various metabolic pathways by a process commonly known as the Maillard reaction. Typically, these compounds are produced nonenzymatically through the condensation of carbonyl groups from reducing sugars with free amine groups found in nucleic acids, proteins, or lipids, which is subsequently followed by additional rearrangements that result in stable, irreversible end products. AGEs are increasingly implicated in the aging, development, and progression of numerous diseases, including neurodegenerative disorders.

Oxidative damage to biomolecules in the CNS can interfere with cellular processes, including neurotransmission and cellular signaling. This damage is responsible for neurodegeneration in diseases such as Alzheimer’s disease (AD), Parkinson’s disease (PD), Huntington’s disease (HD), amyotrophic lateral sclerosis (ALS), and other neurodegenerative conditions (Olufunmilayo et al. 2023). Oxidative damage mediated by ROS can disrupt mitochondrial function, impair energy production, and lead to further ROS generation, forming a vicious cycle that exacerbates neuronal damage. Increased ROS are known to modulate signaling pathways in AD and PD, involving Nrf2, PI3K/AKT/GSK3β, p38 MAPK/NF-κB, and JNK/p53, contributing to neuronal death and disease progression (Jha et al. 2015). ROS can also activate c/EBPβ/AEP signaling, driving AD and PD pathogenesis and promoting neuroinflammation, lipid accumulation, and increased formation of amyloid-β (Aβ) in AD, as well as the loss of dopaminergic neurons in PD (Zhou et al. 2022).

The concerted action of several stressors, including calcium dysregulation and mitochondrial dysfunction, can disrupt neurocircuitry and diminish the connections among the hippocampus, amygdala, and cortex, ultimately resulting in behavioral and cognitive deficits. It has been proposed that strict regulation of oxidative stress, whether through the enhancement of antioxidant defense systems or by directly neutralizing pro-oxidants, may mitigate neurodegenerative and psychiatric symptoms (Salim et al. 2017).

### Astrocytes and microglia in the context of oxidative stress in neurodegenerative disorders

Astrocytes, also known as astroglia, are the most abundant glial cells in the central nervous system (Gradisnik et al. 2023). Astrocytes, together with microglia, are two categories of glial cells that are essential for preserving homeostasis and facilitating neuronal function.

Astrocytes are also involved in managing the blood‒brain barrier and engaging in the processes of synaptic formation and pruning. Since oxidative stress is known to induce inflammation leading to concurrent astrogliosis, astrocytes are recognized for their role in managing inflammatory responses and innate immunity within the central nervous system (Pekny et al. 2016; Rizor et al. 2019). Reactive astrogliosis, an important characteristic of various CNS disorders, refers to the phenomenon in which astrocytes react to stimuli, injury, or disease by altering their quantity, shape, and functionality. This phenomenon represents a multifaceted response to various challenges, including increased oxidative stress, inflammation, trauma, or infection (Rizor et al. 2019; Sofroniew et al. 2009).

ROS are known to activate inflammasomes, such as astrocytic NOD-like receptors (NLRs), specifically those containing leucine-rich repeats (LRRs), and pyrin domain-containing protein 3 (NLRP3). NLRP3 is a cytosolic multiprotein complex that, upon activation by various stimuli, such as increased ROS levels, facilitates the recruitment and activation of procaspase-1. Activated caspase-1 then cleaves and activates interleukin-1β (IL-1β), a key inflammatory cytokine, leading to an inflammatory response (Strowig et al. 2012; Zhou et al. 2011).

Microglia are immune cells that act as resident macrophages of the CNS and regulate the development of the brain, maintenance of the neuronal network, and many other functions, such as synaptic plasticity, learning, and memory (Colonna et al. 2017). Although microglia are crucial for preserving brain health, they play a key role in the interplay between oxidative stress and neurodegeneration. Their impairment, especially in response to oxidative stress, can lead to neurodegeneration (Simpson et al. 2020).

Microglia can exist in two distinct activation states, M1 and M2 (Appel et al. 2011). M1 microglia are proinflammatory and can be neurotoxic. M2 microglia, on the other hand, are associated with anti-inflammatory effects and neuroprotective properties, helping to repair damaged tissue. The two-faced behavior of microglia involves both the progression and alleviation of the pathophysiological mechanisms associated with neurodegeneration. Hyperactivated microglia can generate excessive ROS, leading to oxidative stress that can damage neurons and suppress the brain’s antioxidant defense systems, thereby advancing the progression of neurodegenerative disorders. Activated microglia can secrete proinflammatory cytokines and chemokines, signaling molecules that can further exacerbate neuronal injury.

The bidirectional communication between microglia and astrocytes, which is mediated by secreted factors and direct cell‒cell interactions, plays a crucial role in maintaining neuronal function and responding to injury or disease. Oxidative stress-mediated release of proinflammatory cytokines by astrocytes ultimately stimulates microglia, thereby sustaining neuroinflammation (Zuo et al. 2019; Ehrlich et al. 1998; Bhusal et al. 2023).

Microglia are among the most significant cell types involved in neurodegeneration in Alzheimer’s disease (Chen et al. 2022). As a key element of the brain’s innate immune system, microglia play a crucial role in combating Alzheimer’s disease (AD) pathology, particularly the burden of amyloid plaques. Activated microglia create a barrier that arrests Aβ plaques, thereby restricting their spread and reducing Aβ-induced neuronal damage. In contrast, a recent report revealed a significant relationship between the migration of host-activated microglia and the increased formation of new Aβ plaques in previously unaffected donor grafts (D’Errico et al. 2022).

Although the activation of microglia can lead to neuroinflammation and oxidative damage, recent research has revealed the protective role of microglia (Colonna et al. 2017). Understanding the activation mechanism will help reveal potential therapeutic approaches for neurodegenerative disorders. Specific cell surface receptor expression, polarization responses, and the release of inflammatory mediators are typical features of activated microglia.

Oxidative stress triggers a phenotypic transition toward a proinflammatory M1 state, characterized by the release of proinflammatory cytokines, particularly IL-1β and TNF-α. The proinflammatory M1 subtype is activated by interferon gamma (IFN-γ), a cytokine with diverse functions in the immune system, and the bacterial endotoxin lipopolysaccharide (LPS) (Goldmann et al. 2016). These two key players of the immune system, interferons and lipopolysaccharides, are both capable of activating Janus kinases (JAKs) and signal transducers and activators of transcription (STATs) signaling pathways via Toll-like receptor 4 (TLR4) (Bharadwaj et al. 2020). LPS, which is a component of gram-negative bacteria, triggers TLR4, resulting in downstream signaling cascades that include the activation of JAKs and STATs. Similarly, IFNs also engage receptor complexes that activate the JAK‒STAT pathway. The interaction between these pathways is intricate, with LPS-induced IFN-β potentially augmenting TLR4-mediated signaling and vice versa (Wendimu and Hooks 2022).

Activated microglia, particularly those polarized to the M1 phenotype, exhibit increased expression of both cluster of differentiation 45 (CD45) and MHC class II molecules (MHCII), which are involved in antigen presentation and the initiation of inflammatory responses (Boche et al. 2013; Chhor et al. 2013). While CD45 and MHC class II molecules are involved in the immune response, their direct role in the stimulation of proinflammatory cytokines is not known. However, they play a key role in T-cell activation, which can indirectly stimulate the production of various proinflammatory mediators. These may involve cytokines such as tumor necrosis factor-α (TNFα) and interleukins (IL-1β, IL-6, and IL-12), chemokines (CCL12 and CXCL10), ROS/RNS, inducible nitric oxide synthase (iNOS), and cyclooxygenase-2 (COX-2) (Chhor et al. 2013; Nakagawa and Chiba 2015; Chauhan et al. 2021).

Taken together, while M1 microglia are important in the innate immune response, their persistent activation under pathological conditions contributes to oxidative stress, neuroinflammation, and neurotoxicity (Lehnardt 2010).

**Signaling pathways in microglia and astrocytes.** Toxic proteins, such as amyloid-β in the case of Alzheimer’s disease and the mutant protein huntingtin in the case of Huntington’s disease, can activate the NF-kB signaling pathway within microglia. Under both conditions, activated NF-kB is involved in the production of proinflammatory cytokines (Dash et al. 2025; Singh and Singh 2020; Sun et al. 2021a, b). Both of these neurodegenerative disorders involve other significant pathways, such as the kynurenine pathway (KP), a major metabolic route through which the amino acid tryptophan is activated in microglia. Dysregulation of the kynurenine pathway involves imbalances in the metabolites produced during tryptophan breakdown and results in the overproduction of neurotoxic species such as 3-hydroxykynurenine and quinolinic acid (Dantzer et al. 2017). These metabolites can contribute to neuronal damage, neuroinflammation, and dysfunction and have been observed in increased quantities in patients suffering from both of these disorders.

The activation of NF-κB in astrocytes appears to exert both harmful and beneficial effects on neurodegenerative disorders, particularly AD (Phatnani and Maniatis 2015). While A1 astrocytes are characterized by their proinflammatory and neurotoxic properties, A2 astrocytes are known for their neuroprotective and tissue repair functions. The activation of the NF-κB signaling pathway plays a vital role in the transformation of astrocytes into the A1 phenotype. In contrast, the signal transducer and activator of transcription 3 (STAT3) pathway is implicated in the promotion of the neuroprotective A2 phenotype. Damage to astrocytes precedes and leads to demyelination and the death of oligodendrocytes in conditions such as Alexander disease and vanishing white matter (Nutma et al. 2020).

In addition to NF-kB, another related inflammatory signaling pathway, mitogen-activated protein kinase (MAPK), which encompasses the extracellular signal-regulated kinase (ERK), c-Jun N-terminal kinase (JNK), and p38 MAPK pathways, is known to be activated by amyloid-β (Islam et al. 2023) and results in the release of proinflammatory cytokines, thereby exacerbating neuronal inflammation.

Receptor-interacting protein kinase 1 (RIPK1) is important in the context of neurodegenerative diseases, especially in microglia (Mifflin et al. 2020). RIPK1 is involved in various cell survival and death pathways. The increased activity of RIPK1 in microglia is correlated with increased ROS formation and the release of proinflammatory cytokines and chemokines, thereby contributing to neuroinflammation and the progression of neurodegenerative diseases such as Alzheimer’s disease, multiple sclerosis, and amyotrophic lateral sclerosis. The activation of RIPK1 may also result in microglial dysfunction, which could hinder the capacity of microglia to eliminate cellular debris, including amyloid plaques, which are found in Alzheimer’s disease. Under certain conditions, the activation of RIPK1 can facilitate cell death pathways, such as necroptosis, which may lead to neuronal injury. Inhibiting the kinase activity of RIPK1 has demonstrated potential in preclinical research aimed at treating neurodegenerative conditions.

Triggering receptor expressed on myeloid cells 2 (TREM2) is a cell surface receptor that is expressed on myeloid cells, including microglia, and plays a role in the immune response in neurodegenerative and inflammatory diseases (Walter 2016). Mutations in TREM2 have been linked to a heightened risk of amyotrophic lateral sclerosis (ALS) (Deming et al. 2018). Although this pathway shares some similarities with pathways involved in microglial activation, it is crucial to recognize that the specific triggers and subsequent effects may vary among these neurodegenerative diseases owing to the unique pathological characteristics of each condition.

In summary, although microglia play a vital role in maintaining brain health, their dysfunction—especially as a result of oxidative stress—can greatly exacerbate neurodegenerative diseases. Gaining insight into the processes governing microglial activation and oxidative stress is essential for the formulation of effective treatments for these debilitating conditions.

### Mitochondrial dysfunction in neurodegenerative diseases

Mitochondria are vital organelles that play crucial roles in energy production, cell death regulation, and various cellular processes. To support neuronal activity, neurons require a significant energy supply and proper mitochondrial function (Johri and Beal 2012a; Spina et al. 2025). Optimal mitochondrial function is controlled by several mechanisms, among which mitophagy, a specialized form of autophagy that removes superfluous or dysfunctional mitochondria, plays a key role. In other words, mitophagy is a crucial mechanism for mitochondrial quantity and quality control (Chen et al. 2020).

Mitochondrial dysfunction is a defining feature of idiopathic neurodegenerative disorders, such as Parkinson’s disease, amyotrophic lateral sclerosis, Alzheimer’s disease, and Huntington’s disease (Antico et al. 2025; Yang 2025). Mitochondrial dysfunction further affects intercellular communication and signaling pathways, calcium signaling and apoptosis, and lipid biosynthesis, all of which are involved in the development of neurodegenerative diseases (Keogh et al. 2015).

Dysfunctional mitochondria contribute to neurodegeneration by increasing the production of ROS and causing oxidative damage. Mitochondrial DNA (mtDNA), which is located in proximity to the site of ROS production and devoid of protective mechanisms such as histones, is especially susceptible to damage, resulting in mutations that further hinder the production of adenosine triphosphate (ATP) (Alexeyev et al. 2013). Increased ROS can disrupt the mitochondrial membrane potential (ΔΨm) and increase the permeability of the transition pore (MPTP), allowing the transport of molecules through the inner mitochondrial membrane and regulating calcium, ultimately resulting in neuronal injury (Langston et al. 1983). A decrease in the mitochondrial membrane potential (ΔΨm) triggers the opening of mitochondrial permeability transition pores, which in turn results in the release of cytochrome c (cyt c) and other proapoptotic factors from the intermembrane space. This process initiates the formation of the apoptosome and the activation of caspases, ultimately culminating in apoptosis (Green et al. 2011). The intrinsic pathway of apoptosis is also associated with the protein Apaf-1 (Apoptotic Protease-Activating Factor 1), which acts as a scaffold protein, assembling the apoptosome — a complex that activates caspases (Lauber et al. 2001).

Bcl-2 and Bax are key players in mitochondrial membrane damage and apoptosis (Qian et al. 2022). Mitochondrial membranes are composed of Bcl-2 proteins that have the capacity to either support or suppress cell death via interactions with various other proteins. Pro-apoptotic proteins, including Bax and Bad, for instance, aid in the opening of the mitochondrial permeability transition pore. Conversely, antiapoptotic proteins such as Bcl-2 and Bcl-xL inhibit cell death by binding to and inactivating proapoptotic proteins.

Abnormally toxic proteins, which are typical of neurodegenerative disorders, such as amyloid-β or α-synuclein, disrupt cell membrane integrity and inhibit the oxidative phosphorylation (OXPHOS) system, as manifested by decreased ATP production (Kawamata et al. 2017). The interaction of these toxic proteins with mitochondrial proteins and specific neurons can spontaneously generate ROS in the brain. This increased ROS accumulation triggers a damaging cycle that affects the ETC and mtDNA, further exacerbating metabolic decline. Dysfunctional ETC complexes can release electrons at a relatively high rate, thereby intensifying oxidative stress (Zhao et al. 2019a, b).

An altered electron transport chain, complex I deficiency, and impaired function of citric acid cycle enzymes (Sharma et al. 2009) are key pathologies contributing to diminished brain metabolism. Certain therapeutic approaches seek to stabilize complex I or offer alternative energy sources, although clinical outcomes have been limited thus far (Zhu et al. 2010; Clemente-Suárez et al. 2023).

Apoptosis-inducing factor (AIF) is a mitochondrial protein that promotes both cell survival and cell death, especially in the context of neurodegeneration (Bano et al. 2018). It contributes to the preservation of mitochondrial structure and functionality; however, toxic proteins such as amyloid-β may trigger its release from the mitochondria, initiating a caspase-independent mechanism of programmed cell death. The release of AIF and its subsequent translocation to the nucleus can result in DNA fragmentation and accelerate neurodegeneration.

In addition, abnormally toxic proteins may interfere with the influx of proteins into mitochondria and diminish the activity of mitochondrial proteins such as amyloid-beta binding alcohol dehydrogenase (ABAD), the α-ketoglutarate dehydrogenase complex (αKGDH), and cyclophilin D (Eckert et al. 2011). Furthermore, toxic proteins disrupt the regulation of mitochondrial Ca^2+^, leading to its overload, which subsequently results in a decrease in the ΔΨm and an increase in ROS production, the opening of the mitochondrial permeability transition pore (MPTP), the release of proapoptotic factors, and a reduction in ATP production (Görlach et al. 2015).

It is increasingly evident that processes associated with PINK1 and PARKIN can influence neurodegeneration and neuroinflammation. This modulation occurs through the elimination of dysfunctional mitochondria, the regulation of mtDNA release, or the promotion of neuroprotective and anti-inflammatory phenotypes (Quinn et al. 2020a, b). PINK1, also known as PTEN-induced kinase 1, is a serine/threonine-protein kinase that is incorporated into mitochondria and plays a crucial role in protecting cells from mitochondrial dysfunction. Under healthy conditions, PINK1 is rapidly degraded; however, damaged mitochondria accumulate PINK1 on the outer membrane. Damaged mitochondria with accumulated PINK1 consequently recruit Parkin, a ubiquitin ligase, from the cytoplasm, where it is localized. Parkin then attaches ubiquitin tags to various mitochondrial proteins, marking them for degradation (Narendra et al. 2024). These two proteins, PINK1 and Parkin, are implicated in the development of Parkinson’s disease.

Enhancing the mitophagy pathway may represent a novel therapeutic approach for targeting the underlying pathogenic causes of familial forms of Parkinson’s disease and amyotrophic lateral sclerosis, with the potential to maintain neuroprotection and disease modification. Molecular and preclinical model-based evidence now supports the targeting of mitophagy via small-molecule-based approaches for selective mitophagy enhancement. Several promising activators are currently entering phase I clinical trials for the first time (Antico et al. 2025).

### Oxidative stress and specific brain regions

The central nervous system (CNS) has a notably high metabolic rate, which predisposes both neurons and glial cells to produce significant amounts of ROS/RNS, increasing their susceptibility to oxidative damage (Bai et al. 2022). Biochemically, distinct neurons clearly exhibit varying degrees of susceptibility to oxidative stress. Thus, the degree of oxidative stress-mediated damage depends on the affected CNS region. Research has indicated that the hippocampus, amygdala, cerebellar granule cells, and prefrontal cortex are among the most vulnerable to oxidative stress (Wang et al. 2010) and, as a result, are believed to be the initial regions that experience functional decline (Salim et al. 2017). The significant differences in the sensitivity of individual brain structures to oxidative stress result in different levels of intrinsic oxidative stress in these structures, different levels of ROS/RNS signaling in these structures, and differences in DNA repair capacity and calcium signaling, which result in differences in glial-neuronal communication mechanisms and, subsequently, their modification within a cell.

It has been reported that the amygdala and prefrontal cortex may experience dendritic changes, particularly in the context of chronic stress (Radley et al. 2006). Reports indicate that dendritic shrinking in the medial prefrontal cortex alongside dendritic expansion in neurons of the amygdala occurs in response to stress factors. It is well documented that stressful stimuli can modify the dendritic structure and neuronal connections within the prefrontal cortex (Liston et al. 2009). These findings indicate that oxidative stress within the brain undermines the biochemical integrity of both the hippocampus and the amygdala.

The hippocampus is an essential structure of the brain that plays a vital role in learning and memory, especially in the processes of creating new long-term memories and spatial navigation. Oxidative stress triggers biochemical alterations in the hippocampus that ultimately impact neuronal connections and functionality. One of the most affected regions within the hippocampus is the “cornu ammonis”, a specific series of subfields (CA1, CA2, CA3, CA4) crucial for memory and spatial navigation. CA3 neurons, specifically pyramidal cells within the CA3 region, display structural plasticity and the capacity for remodeling and regeneration (Sousa et al. 2000). Recent research has revealed a transition in selective vulnerability from CA1 neurons to those in CA3 neurons (Einenkel et al. 2024). Notably, notable differences in the structure, function, and susceptibility to oxidative damage are present between the CA1 and CA3 regions of the hippocampus (Salim 2017). These variations play crucial roles in defining their unique contributions to memory processing and various cognitive functions.

The dentate gyrus is a part of the brain’s hippocampal formation and is important in memory formation, particularly in the encoding of new memories and pattern separation. Various studies have indicated that the pyramidal cells in CA3 and the granule cells in the dentate gyrus (DG) are prone to oxidative stress, whereas other studies have shown that the pyramidal cells in CA1 are more vulnerable to oxidative damage (Uysal et al. 2012). Nonetheless, the region-specific increase in oxidative stress within the cornu ammonis regions CA1 and CA3, as well as the dentate gyrus, is crucial and can lead to significant functional implications. This is especially important considering that the DG plays a key role in learning and memory processes, whereas the ventral hippocampus is associated with anxiety and depression.

More pronounced cellular damage in CA3, exceeding that observed in CA1, is influenced by various factors, such as methodology, species, age, and duration of observation. Although the fundamental cellular mechanisms are currently not known, multiple factors, including disruptions in the trafficking of tetrameric AMPA-type ionotropic glutamate receptors (de León-López et al. 2025), the level of oxidative stress, the disturbed balance of pro- and antiapoptotic Bcl-2 factors, and the extent of mitochondrial damage, play a role in the vulnerability of either CA1 or CA3.

### Aging – a common denominator of neurodegenerative diseases

While there are several common risk factors for neurodegenerative diseases, aging appears to be of key importance. Therefore, all impactful interventions should consider the basic mechanisms of the aging process (Hou et al. 2019; Wyss-Coray 2016).

Although neurodegenerative diseases are typical of advanced age, certain studies have suggested that the likelihood of neurodegenerative diseases is linked to early developmental anomalies, indicating that alterations in brain structure may occur significantly earlier than cognitive deterioration (Dean et al. 2014). The apolipoprotein E (APOE) ε4 allele is the major genetic risk factor for the development of sporadic (late-onset) Alzheimer’s disease. MRI analysis of white matter myelin and gray matter volume across different brain regions of infants carrying this genetic risk factor revealed notable differences compared with measurements from noncarriers (Dean et al. 2014). This risk group must be particularly careful because exposure to harmful environmental factors such as trauma, drugs, or environmental toxins, including pesticides, during developmental stages has been suggested to lead to repercussions in later life (for example, by negatively affecting neuroplasticity) (Schaefers et al. 2016).

Quantitative analysis of samples from elderly individuals revealed the accumulation of aggregated toxic proteins, including hyperphosphorylated tau (p-tau), amyloid-β (Aβ), and α-synuclein, which are typical of most common neurodegenerative diseases. Nevertheless, it is still uncertain whether the concentrations of these deposits are correlated with the extent of cognitive decline (Elobeid et al. 2016).

Recent results suggested the molecular and cellular mechanisms, as well as biomarkers, that correlate with aging across various mammalian species (López-Otín et al. 2023). More than a decade ago, nine key hallmarks of aging were categorized into three categories (López-Otín et al. 2013). These three categories, (i) primary hallmarks (DNA instability, telomere attrition, epigenetic alterations, loss of proteostasis), (ii) antagonistic hallmarks (deregulated nutrient-sensing, mitochondrial dysfunction, and cellular senescence) and (iii) integrative hallmarks (stem cell exhaustion and altered intercellular communication), have been recently identified as primary hallmarks—disabled macroautophagy—and two integrative hallmarks—chronic inflammation and dysbiosis (López–Otín et al. 2023). All these hallmarks of aging are observed in the most common neurodegenerative diseases, such as Alzheimer’s disease, Parkinson’s disease, Huntington’s disease, and amyotrophic lateral sclerosis. Ataxia-telangiectasia, a rare genetic condition that affects the function of the nervous and immune systems, has been linked with genetic instability and mitochondrial dysfunction hallmarks (Hou et al. 2019; Wyss-Coray et al. 2016).

Compared with their proliferating counterparts, postmitotic neurons and oligodendrocytes are particularly affected by aging because of their susceptibility to DNA damage. Aging brains are characterized by accumulated DNA damage, which plays a significant role in the incidence of neurodegeneration. Several important hallmarks of aging associated with the phenomenon of oxidative stress and its impact on neurodegenerative diseases are briefly described below.

### Genomic instability and DNA damage

Genomic instability plays a central role in the hallmarks of neurodegenerative disorders. Genomic stability depends on the equilibrium between DNA damage (exogenous and endogenous) and DNA repair pathways. Endogenous or exogenous factors induce various DNA lesions that play a role in both normal and pathological aging. While exogenous factors include chemical, biological, and physical agents, endogenous challenges involve oxidative stress, DNA replication errors, chromosome segregation, and spontaneous hydrolytic reactions. Genetic lesions resulting from these extrinsic or intrinsic sources of damage include point mutations, single- and double-strand breaks, deletions, translocations, telomere shortening, chromosomal rearrangements, defects in nuclear architecture, and gene disruption due to the integration of viruses or transposons (Vijg and Dong 2020). These lesions can be repaired through multiple mechanisms, which tend to diminish in efficiency with aging. The accumulation of DNA damage, inadequate DNA repair, changes in nuclear structure, and telomere shortening promote the aging process.

The most prominent forms of DNA damage associated with neurodegeneration include single- and double-strand breaks, base mismatches, and insertions and deletions, and the key DNA repair mechanisms include base excision repair (BER), mismatch repair, nucleotide excision repair (NER), and DNA double-strand break repair (Jeppesen et al. 2011). Oxidative DNA damage is tightly associated with increased ROS-induced oxidative stress, which is usually accompanied by increased inflammation, contributing to the incidence of chronic diseases such as neurodegenerative diseases and cancer (Thanan et al. 2014).

The base excision repair (BER) pathway is considered the primary repair mechanism of oxidative base damage and single-strand breaks (Maynard et al. 2009), and mutations in genes involved in this pathway can lead to increased DNA damage. DNA damage not only leads to genomic instability but also triggers signaling cascades such as the induction of poly(ADP‒ribose) polymerase 1 (PARP1), which negatively affects DNA repair and chromatin remodeling. This, in turn, enhances PARylation (synthesis of PAR polymers at sites of DNA damage or alterations), resulting in the depletion of NAD^+^, which is a crucial factor for sirtuins, DNA repair, and normal functioning of mitochondria. Sirtuins 1 and 3 are known to facilitate BER and other DNA repair mechanisms (Fang et al. 2017).

#### Mitochondrial dysfunction and degradation in neurodegenerative diseases

Mitochondrial dysfunction is a defining feature of idiopathic neurodegenerative disorders, such as Parkinson’s disease, amyotrophic lateral sclerosis, Alzheimer’s disease, and Huntington’s disease.

To achieve neuronal activities, neurons require a significant energy supply and proper mitochondrial function. ROS-induced oxidative stress causes mitochondrial damage, which is implicated in the aging process and the incidence of various neurodegenerative diseases (Johri and Beal 2012a; Spina et al. 2025). Mitochondrial dysfunction further affects intercellular communication and signaling pathways, calcium signaling, apoptosis, and lipid biosynthesis, all of which are involved in the development of neurodegenerative diseases (Keogh et al. 2015).

Optimal mitochondrial function is regulated by several mechanisms, among which mitophagy, a specialized form of autophagy that removes superfluous or dysfunctional mitochondria, plays a key role. In other words, mitophagy is a crucial mechanism for mitochondrial quantity and quality control (Chen et al. 2020).

Mitochondrial dysfunction is a defining feature of idiopathic neurodegenerative disorders, such as Parkinson’s disease, amyotrophic lateral sclerosis, Alzheimer’s disease, and Huntington’s disease (Antico et al. 2025). Familial variants of Parkinson’s disease and amyotrophic lateral sclerosis frequently involve mutations in genes associated with mitophagy deficits.

PINK1, also known as PTEN-induced kinase 1, is a serine/threonine-protein kinase that is incorporated into mitochondria and plays a crucial role in protecting cells from mitochondrial dysfunction. Under healthy conditions, PINK1 is rapidly degraded; however, damaged mitochondria accumulate PINK1 on the outer membrane. Damaged mitochondria with accumulated PINK1 consequently recruit Parkin, a ubiquitin ligase, from the cytoplasm, where it is localized. Parkin then attaches ubiquitin tags to various mitochondrial proteins, marking them for degradation (Narendra et al. 2024). These two proteins, PINK1 and Parkin, are implicated in the development of Parkinson’s disease.

It is increasingly evident that processes associated with PINK1 and PARKIN can influence neurodegeneration and neuroinflammation. This modulation occurs through the elimination of dysfunctional mitochondria, the regulation of mtDNA release, or the promotion of neuroprotective and anti-inflammatory phenotypes (Quinn et al. 2020a, b).

Enhancing the mitophagy pathway may represent a novel therapeutic approach for targeting the underlying pathogenic causes of familial forms of Parkinson’s disease and amyotrophic lateral sclerosis, with the potential to maintain neuroprotection and disease modification. Molecular and preclinical model-based evidence now supports the targeting of mitophagy using small-molecule-based approaches for selective mitophagy enhancement. Several promising activators are currently entering phase I clinical trials for the first time (Antico et al. 2025).

### Cellular senescence

Cellular senescence is associated with many neurodegenerative diseases. Cellular senescence is a widespread phenomenon that plays significant roles in tissue remodeling, such as in wound healing and embryonic development (Hayflick 1965). This process is characterized by cell cycle arrest and the secretion of inflammatory cytokines. Extended periods of senescence may be detrimental, contributing to the onset of cancer, neurodegenerative disorders, and age-associated disorders (Huang et al. 2022). The most common senescence markers, however, with limited applications, are cyclin-dependent kinase inhibitor (CKI) p21, a tumor suppressor protein p16, and the senescence-associated beta-galactosidase SA-β-gal (SABG).

Cellular senescence is a double-edged sword, underscoring the need to distinguish between physiological and pathological senescence. While senescence plays a role in the onset of age-related chronic disorders, dysfunction of the immune system, and cancer progression, it also serves as a crucial mechanism that safeguards against tumor formation. This distinction is vital for the development of pharmacological interventions.

Cells exhibiting elevated levels of DNA damage enter a state of senescence and cease to proliferate. Owing to the high energy demand associated with increased ROS formation in neurons, brain tissue may accumulate DNA damage, which typically increases with age and is accompanied by a decline in DNA repair capacity (Maynard et al. 2015).

Neurons, as post-mitotic cells, exhibit increased sensitivity to DNA lesions and vulnerability to DNA repair mechanisms. Neurons that have a continuously activated DNA damage response (DDR) pathway display numerous characteristics indicative of cellular senescence and are thus termed senescence-like neurons (Fielder et al. 2017).

DNA damage can trigger a senescence-like state in mature postmitotic neurons in vivo. Approximately 60% of Purkinje neurons and 30% of cortical, hippocampal, and peripheral mouse neurons presented a significant level of DNA damage, activation of p38MAP kinase, increased ROS production, interleukin IL-6 production, and senescence-associated β-galactosidase activity (SABG). (Jurk et al. 2012). Thus, senescence-like neurons could be a source of oxidative and inflammatory stress, contributing to the aging of the brain.

Autophagy is associated with cellular senescence; however, whether autophagy has a positive or negative impact on senescence has not been sufficiently proven (Narita et al. 2011). Processes that may link these two phenomena include oxidative stress, mitochondrial dysfunction, inflammation, telomere shortening, the chronic DNA damage response (DDR) and other phenomena (Bhatia-Dey et al. 2016).

### Deregulated nutrient sensing and altered metabolism

Caloric restriction (CR) refers to a dietary approach that entails a reduction in calorie consumption while avoiding malnutrition (Redman et al. 2011). Interestingly, metabolic dysfunction is common in individuals with neurological conditions and may be associated with reduced NAD^+^ levels, mitochondrial impairment, and oxidative stress.

Animal studies have demonstrated the ability to prolong lifespan and possibly postpone age-related illnesses. Although human studies are still in progress, CR appears to hold potential for enhancing numerous health indicators, including neuroprotective benefits (Fontana et al. 2010). The major pathways and molecules involved in nutrient sensing are insulin-like growth factor 1 (IGF1), mechanistic target of rapamycin (mTOR), AMP-activated protein kinase (AMPK), and sirtuins (López-Otín et al. 2013).

### Epigenetic modifications

The epigenetic alterations associated with aging include DNA methylation, histone modifications, the regulation of noncoding RNA (ncRNA), RNA modifications, and chromatin remodeling. Each of these factors plays a role in the regulation of the aging process, thereby contributing to diseases related to aging (Wang et al. 2022). In the regulation of aging, key phenomena include interactions between epigenetic changes and other elements, such as genetic predispositions and the microbiome. Additionally, the role of environmental factors and physiological and psychological conditions in driving epigenetic modifications during aging is not well understood. Moreover, given that aging is a gradual process that progresses over many years in humans, monitoring epigenetic alterations throughout this entire continuum is essential to gain clearer insight into the specific contributions of epigenetic regulation at each phase of aging. To target epigenetic alterations, a range of therapeutic approaches have been formulated. Geroprotective medications aimed at longevity-associated histone acetylation, including NAD^+^ precursors and STACs, metformin, rapamycin, and other pharmaceuticals, have demonstrated beneficial effects in mitigating age-related pathologies and modulating epigenetic changes associated with aging in preclinical investigations (Howitz et al. 2003; Yin et al. 2022; Zhu et al. 2021).

## Alzheimer’s disease

Alzheimer’s disease (first identified by German doctor Alois Alzheimer in 1906) is the most common form of dementia, with approximately 10 million new cases each year (Masters et al. 2015). Alzheimer’s disease accounts for 55–75% of all dementia cases (Seshadri et al. 2007). The sporadic form is the most common form, accounting for ~ 95% of cases, with an average onset at age 80. The disease typically lasts approximately 9 years, although symptoms are usually preceded by a preclinical stage that can last for more than two decades. The familial form of Alzheimer’s disease (FAD) is rare, occurs in much younger subjects aged ~ 40 years, and is caused by inherited genetic mutations in several genes (Weinstock et al. 2025). Both sporadic and familial forms share various clinical and pathological characteristics, including representative biomarkers.

Alzheimer’s disease is characterized by a gradual decline in cognitive health, which can be associated with a notable decrease in brain volume among AD patients compared with healthy individuals (Mattson et al. 2004). This atrophy is a consequence of synaptic degeneration and neuronal death, particularly within the hippocampus, which are crucial for memory and spatial orientation.

The major risk factors for Alzheimer’s disease are advanced age, genetic predispositions, peripheral arterial disease, brain injuries, and environmental neurotoxins (lead, mercury, aluminum, industrial organophosphates, and other factors).

Alzheimer’s disease is characterized as a gradually progressing condition caused by the accumulation of insoluble amyloid-β (Aβ) and tau proteins. Amyloid-β forms plaques outside cells and in blood vessel walls, whereas tau proteins form neurofibrillary tangles (Breijyeh and Karaman 2020). Amyloid-β is produced when the amyloid precursor protein (APP) undergoes proteolytic cleavage by β-secretase (BACE1) and γ-secretase. Usually, amyloid-β accumulation occurs before neurofibrillary tangles and neuritic changes, mainly starting in the frontal and temporal lobes, the hippocampus, and the limbic system. Less often, it begins in the parietal and occipital lobes, with the hippocampus remaining relatively unaffected (Masters et al. 2015).

### Biomarkers and clinical diagnosis of Alzheimer’s disease

Diagnosing Alzheimer’s disease involves recognizing specific symptoms and ruling out other possible causes of dementia. A comprehensive medical exam typically includes neurological tests, blood tests, and brain imaging, such as CT, MRI, or PET/CT scans (Gunes et al. 2022).

MRI plays a vital role in diagnosis by revealing structural brain changes, particularly atrophy in areas such as the hippocampus and temporal lobes. It helps evaluate brain volume loss and identify other factors related to dementia (Ortner et al. 2019). Functional magnetic resonance imaging (fMRI) can track brain activity and connectivity. Additionally, positron emission tomography (PET) enables the quantification and visualization of tau protein tangles in the brain, thereby indicating the extent of tau pathology. Fluorodeoxyglucose positron emission tomography (FDG PET) evaluates brain glucose metabolism and reveals that hypometabolism (reduced glucose uptake) is associated with AD.

MRI has been successfully used to monitor amyloid-related imaging abnormalities (ARIs) (Greenberg et al. 2025). ARIA refers to the most impactful adverse effect associated with anti-amyloid-β immunotherapy. MRI modifications include ARIA-E (edema and effusion in the brain parenchyma or sulci) and ARIA-H (cortical superficial siderosis, cerebral microhemorrhages, and other adverse effects). The majority of ARIAs are mild, but approximately 5% of them are serious and require hospitalization. The development and testing of new approaches to predict and treat ARIA is a key strategy for the use of safe anti-amyloid-β immunotherapy.

Cerebrospinal fluid (CSF) biomarkers also reveal brain pathology. The main CSF markers for Alzheimer’s disease are amyloid-beta 1–42 (Aβ42), total tau (t-tau), and phosphorylated tau (p-tau) (Blennow et al. 2017). Decreased concentrations of Aβ42 in CSF are a defining characteristic of AD, given that it constitutes a significant element of the amyloid plaques present in the brain. The Aβ42-to-Aβ40 ratio in CSF is more sensitive than Aβ42 alone. Increased concentrations of both total tau (t-tau) and phosphorylated tau (p-tau) in CSF are likewise indicative of AD, manifesting increased neurodegeneration and the development of neurofibrillary tangles.

One of the key blood–brain biomarkers is phosphorylated tau (p-tau) (Grande et al. 2025). P-tau is a protein with attached phosphate groups, representing a significant characteristic of the AD brain. Two forms of p-tau, p-tau181 and p-tau217, have demonstrated potential as blood-based biomarkers for Alzheimer’s disease (AD), possibly aiding in distinguishing AD from other neurodegenerative disorders. A recently published analysis of plasma %p-tau217 (the ratio of phosphorylated tau217 to nonphosphorylated tau), conducted using mass spectrometry, demonstrated clinical equivalence to the Food and Drug Administration (FDA)-approved cerebrospinal fluid tests currently used to identify Alzheimer’s disease pathology (Barthélemy et al. 2024).

The evaluation of oxidative stress markers in the brain can clarify the role of oxidative damage in AD. The most sensitive markers of oxidative stress include oxidized proteins, lipids, and DNA, as well as dysregulated metabolism of redox-active iron and copper and nonredox zinc.

MicroRNAs (miRNAs) are small, single-stranded noncoding RNAs that play crucial roles in neurological activity by mediating synaptic plasticity, apoptosis, and other functions. An increasing number of studies have demonstrated that the dysregulation of microRNAs (miRNAs) found in blood and cerebrospinal fluid (CSF) may contribute to the pathogenesis of Alzheimer’s disease (AD) and could serve as candidate biomarkers (Sun et al. 2021a, b).

The patterns of gene expression exhibit considerable variation across various types of brain cells, such as neurons and glial cells, as well as among subtypes within these classifications (Mathys et al. 2019). Examining gene expression patterns may provide valuable insights into the progression of this disease and identify possible drug targets.

### Oxidative stress-related pathways in Alzheimer’s disease

AD is a multifactorial disease that involves several different etiopathogenic mechanisms. Various hypotheses underlying AD have been proposed. These include amyloid-β, dysregulation of redox and nonredox metal ions, cholinergic neuronal damage, inflammation, and other pathologies. All of these pathologies share, to some extent, oxidative stress as a common denominator (Bai et al. 2022). The brain, on the one hand, uses a high amount of oxygen and, therefore, is vulnerable to oxidative stress; on the other hand, it is not sufficiently equipped with antioxidant defense systems. While low-to-medium ROS level-mediated mild oxidative stress can induce beneficial effects in neurons, termed the adaptive response, which is characterized by increased neuroprotection, neuroplasticity, and suppressed neurodegeneration, high levels of ROS cause detrimental effects on neurons (Bai et al. 2022). The adaptive response to mild oxidative stress is termed neurohormesis, a specific form of hormesis (Trovato Salinaro et al. 2018).

The mechanism of neuroprotection can be explained by the binding of NF-κB elements to the Mn-SOD (SOD2) gene, which in turn leads to the induction of Mn-SOD mRNA (Sompol et al. 2006; Calabrese et al. 2010). In addition, S-nitrosylation confers protection against oxidative stress. This mechanism involves the incorporation of a nitrosyl group into the reactive thiol group of a cysteine, forming S-nitrosothiol (SNO), a multifaceted posttranslational modification that plays a crucial role in the signal transmission mediated by NO^•^ (Fernando et al. 2019; Calabrese et al. 2007).

### Amyloid-β peptide

Amyloid-β is a peptide ranging from 38 to 43 amino acid residues in length. It is formed from the type I transmembrane amyloid precursor protein (APP), which is enzymatically cleaved by β- and γ-secretases (Butterfield et al. 2007). The cleavage process is known as amyloidogenic pathway. The exact physiological role of APP is not known; however, its role in synaptic plasticity, memory, and brain development has been established (Cheignon et al. 2018; Jakob-Roetne et al. 2009). The site of APP cleavage by γ-secretase determines the lengths of the formed peptides, ranging from Aβ1–38 to Aβ1–43. Nevertheless, the most populated form generated in the brain is Aβ1–40, with Aβ1–42 being produced to a lesser degree. A decade ago, a new APP pathway, resulting in the generation of proteolytic higher-molecular-mass carboxy-terminal fragments (termed CTF-η) in the hippocampus, was reported (Willem et al. 2015). The cleavage process of APP by η-secretase occurs at amino acids 504–505.

Presenilin-1 (PS-1) and presenilin-2 (PS-2) are proteins encoded by the PSEN1 and PSEN2 genes, respectively. PS-1 and PS-2 constitute the catalytic domain of the γ-secretase complex, which is responsible for cleaving APP (Wiseman et al. 2015). Mutations in PSEN1/PSEN2 have been found to cause early-onset AD. Apolipoprotein E is involved in the clearance of amyloid-β, and its APOE gene (APOEε4 allele) represents the major risk factor for late-onset AD.

Amyloid-β plaque (senile plaques or neuritic plaques) formation in the context of Alzheimer’s disease represents the key component of the amyloid cascade hypothesis proposed in 1991 (Selkoe et al. 1991; Hardy et al. 1992). The scientific community has attempted to employ this hypothesis to clarify the pathogenesis of AD. The central tenet of this hypothesis is the abnormal extracellular accumulation and aggregation of amyloid-β into β-sheet structures. The mechanism of this process involves the misfolding and aggregation of Aβ peptides, resulting in the formation of amyloid plaques, which may contribute to neurotoxicity.

Amyloid-β peptides in the form of monomers aggregate (or self-assemble) to form larger structures, first forming soluble oligomers possessing neurotoxic properties. Oligomers can cross cell membranes and trigger a series of events resulting in cell death (Glabe et al. 2006). Oligomers can further assemble to form protofibrils, highly neurotoxic aggregates, and insoluble fibrils. The aggregated fibrils then form amyloid plaques. All these amyloid-β aggregates, especially fibrils, can disturb neuronal function and cause neuronal damage and synaptic dysfunction manifested by impaired learning and memory functions. The accumulation of amyloid-β can trigger the hyperphosphorylation of tau, leading to the formation of neurofibrillary tangles (NFTs), which are closely linked to cognitive decline and neuronal loss (Moloney et al. 2021).

### Cu, Zn and Fe binding to the amyloid-β peptide

X-ray absorption spectroscopy (XAS), scanning transmission X-ray microscopy (STXM), micro-PIXE analysis, and X-ray fluorescence microscopy revealed that amyloid plaques contain redox-active copper and iron and redox-inactive zinc (Rajendran et al. 2009; Cheignon et al. 2018). The results confirmed increased metal concentrations within the amyloid plaques compared with those in the surrounding tissue: the concentration of iron doubled (85 ppm compared with 42 ppm), the concentration of copper nearly tripled (16 ppm compared with 6 ppm), and the concentration of zinc increased 2.5-fold (87 ppm compared with 34 ppm).

The homeostasis of redox-active Cu and Fe, along with nonredox Zn, is influenced by a complex interaction of factors, including redox signaling and antioxidant defenses. The dual nature of copper involves, on the one hand, essential catalytic roles supported by its regulation through (i) cuproenzymes that require Cu as a cofactor and (ii) Cu transporters that manage its cellular trafficking; on the other hand, it participates in redox cycling reactions that produce reactive oxygen species such as hydroxyl radicals via the Fenton reaction. This redox-related toxicity is linked to “free” or “labile” copper, which is not bound to ceruloplasmin and can catalyze harmful reactions (Sensi et al. 2018). Amyloid precursor protein (APP) is involved in metal metabolism. Copper enhances APP transcription, and APP facilitates iron efflux from neurons (Schulz et al. 2011).

Zinc and copper share + 2 oxidation states, have similar ionic radii, and therefore tend to form isostructural compounds with structurally similar ligand arrangements. Zinc in the cytosol is maintained at picomolar to nanomolar concentrations and serves as a second messenger in cellular signaling. Zinc levels are controlled by families of zinc transporter proteins (ZnTs) and ZIP (Zrt- and Irt-like) proteins (Chen et al. 2024). Eukaryotic cells store zinc either (i) in the lumen of intracellular organelles such as mitochondria, the endoplasmic reticulum, and lysosomes or (ii) bound to metal-binding proteins−metallothioneins. Pathological conditions, including neurodegeneration, are manifested by an increase in the concentration of metals in neurons via transsynaptic influx.

Dietary iron is present in the form of heme iron, which is derived from animal sources and is absorbed more effectively than nonheme iron, which is found in plant-based foods and dietary supplements. Before absorption, nonheme iron is converted from its ferric (Fe^3+^) state to the ferrous (Fe^2+^) state by the ferrireductase Dcytb, which is located on the brush border of enterocytes (Liu et al. 2018). Fe^2+^ enters mucosal epithelial cells via a divalent metal ion transporter (DMT1), which is located primarily in the small intestinal epithelial cell membrane. DMT1 is expressed in the cerebellum, hippocampus, and substantia nigra and in nonneuronal cells such as microglia, astrocytes, and Schwann cells.

### Structures of copper, zinc, and iron-binding sites on amyloid-beta

Copper, in general and specifically in the context of amyloid-beta, can exist in two oxidation states: cupric (Cu(II), Cu^2+^) and cuprous (Cu(I), Cu^+^). Cu(I) has a d^10^ electron configuration, contains no unpaired electrons, is diamagnetic, and favors two-coordinate linear, three-coordinate trigonal planar, and four-coordinate tetrahedral arrangements around the metal ion (Valko et al. 2005). Cu(I) coordinates to amyloid-beta mainly through its histidine residues, specifically His6, His13, and His14 (Hureau et al. 2009; Atrián-Blasco et al. 2018). While some studies suggest the involvement of all three histidines (a triad consisting of His6, His13, and His14) in cupric, Cu(II) binding, the dyad (a pair of His13 and His14 residues) is considered a primary binding site for Cu(I). Therefore, amyloid-beta mainly relies on two histidine residues (the dyad of His13 and His14) to bind Cu(I). The affinity constant has been estimated to range from 10^7^ M^− 1^ to 10^10^ M^− 1^, indicating a strong interaction between Cu(I) and amyloid-beta (Alies et al. 2012a, b).

Cu(II) contains 9d electrons, is paramagnetic, and is characterized by coordination plasticity. Cu(II) binding to proteins is typically a pH-dependent process. Pulsed EPR spectroscopy revealed two major Cu(II) binding sites, I and II, at physiological pH. Most abundantly, primary binding site I is described by Cu(II) coordinated to the N-terminus of amyloid-β, two nitrogens from the imidazole rings of His6 and either His13 or His14 and carbonyls (-C = O) originating from the Asp1‒Ala2 bond (Dorlet et al. 2009; Drew et al. 2009, 2011; Cheignon et al. 2018).

**Fig. 2:**
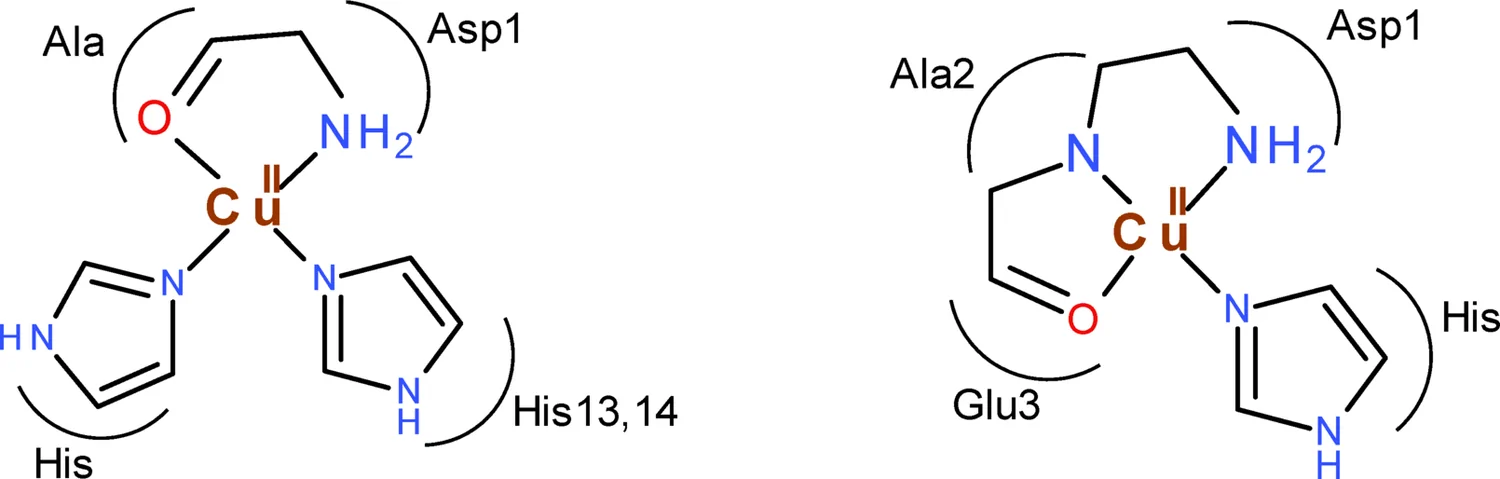
Structure of the amyloid-beta-Cu-binding sites

Binding site II is at physiological pH in equilibrium with site I and is characterized by Cu(II) coordinating to the N-terminal amine (-NH_2_) of Asp1 and then to the -N of the peptide bond connecting Asp1-Ala2 and a carbonyl group (-C = O) of Ala2-Glu3 (Hureau 2018). Distorted square-planar coordination around Cu(II) is completed by -N from the imidazole group of one of the His residues, forming a CuN_3_O core. The resulting geometrical arrangement around Cu(II) is driven by the Jahn–Teller effect (Fig. 2). We cannot exclude the coordination of the apical oxygen to the carboxylic group, resulting in a distorted square-pyramidal arrangement around the Cu(II) ion. The reported binding constants are in the range of 10^7^ M^− 1^ to 10^10^ M^− 1^ (Alies et al. 2012a, b).

Cu(I) generally binds to amyloid-β in a linear, two-coordinate geometry. The imidazole nitrogen atoms of His13 and His14 are the primary ligands for Cu(I). Some studies suggest the presence of a bis-His(Aβ)-Cu(I) complex (specifically the His13-His14 dyad), whereas others propose that His6 may also participate in Cu(I) binding.

Zinc exists exclusively in the stable Zn(II) oxidation state. Both electron paramagnetic resonance (EPR) spectroscopic techniques, continuous wave (cw) and pulsed methods, which are applied in Cu(II) studies, are among the most sensitive techniques for characterizing the coordination environment around metal ions. The limitation of this technique is that the metal ion must be paramagnetic (containing unpaired electron(s)), which is not the case for zinc. In agreement with this, the description of the Zn(II) binding site on amyloid-β is not satisfactory.

NMR spectroscopy was employed to study the zinc-binding domain of the amyloid-β peptide in solution at pH 6.5 (Zirah et al. 2006; Miller et al. 2010; Alies et al. 2016). NMR data of the Zn(II)-amyloid-β complex revealed a 1:1 stoichiometry and the involvement of imidazole nitrogens originating from His6 and either His13 or His14 residues and Glu11 carboxylate ligands in tetrahedral coordination around the Zn(II) cation (Tougu and Palumaa 2012). Affinity constants evaluated by isothermal calorimetry and competition studies resulted in an affinity constant of ~ 10^5^ M^− 1^, which is consistent with a likely interaction under in vivo conditions (Noël et al. 2014; Gaggelli et al. 2008).

Ferric ions (Fe(III) at physiological pH form Fe(III)-oxy and Fe(III)-hydroxy species and precipitate (Valko et al. 2005). The Fe(II)-amyloid-β complex was studied via NMR spectroscopy (Bousejra-ElGarah et al. 2011). The results revealed the involvement of the terminal amine (-NH_2_) and carboxylate (-COOH) groups of Asp1, the Asp1-Ala2 and His6-Asp7 carbonyl (-C = O) groups of peptide bonds, the nitrogen of the imidazole ring of His6, and the imidazole nitrogen, originating either from His13 or His14 (Bousejra-ElGarah et al. 2011; Azimi and Rauk 2012).

### Amyloid-β-Cu/Fe-mediated ROS production

The detection of amyloid-β, the earliest biomarker of Alzheimer’s disease, is accompanied by several direct and indirect hallmarks of increased oxidative stress (Nunomura et al. 2001a, b). These include (i) disturbed redox active metal (Cu, Fe) and redox inert metal (Zn) homeostasis; (ii) interactions of dysregulated metal ions with amyloid-β; (iii) increased levels of lipid peroxidation end products and the formation of breakdown products such as 4-hydroxynonenal, 4-hydroxyhexenal and malondialdehyde, which are known for their reactivity with proteins and neurons, disrupting their function and contributing to neurotoxicity; (iv) oxidized protein side chains, predominantly sulfur-containing side chains (cysteine, methionine) and aromatic side chains (tyrosine, phenylalanine), leading to protein aggregation and dysfunction; (v) DNA oxidation adducts such as 8-oxo-Gua, which cause mutations and genomic instability, mediate cell death and disease progression; and (vi) increased activity and expression of myeloperoxidase and (vii) mitochondrial dysfunction, particularly oxidative damage, which results in less energy and decreased activity of cytochrome c oxidase in the brain in AD (Nakamura et al. 2007).

The binding modes of copper, iron, and zinc with amyloid-β have been described above and are supported by numerous in vivo studies, which have confirmed the occurrence of oxidative damage to neurons (Lovell et al. 1998; Smith et al. 1997). Redox metals can undergo reduction in the presence of biological reductants such as ascorbic acid and glutathione, shifting metals from higher to lower oxidation states and maintaining redox cycling between two states—a process characterized by a cyclical loss and gain of electrons. Redox-active copper and iron, which are coordinated to amyloid-β and interfere with the presence of reductants such as ascorbic acid, can produce reactive oxygen species (ROS), as evidenced by increased oxidative stress biomarkers.

The standard electron reduction potentials for Cu²⁺/Cu and Cu²⁺/Cu^+^ are + 0.34 V and + 0.15 V, respectively, whereas for iron, Fe³⁺/Fe²⁺ and Fe²⁺/Fe are + 0.77 V and − 0.44 V, respectively. These values indicate that ferric ions are more easily reduced to ferrous ions than cupric ions are to cuprous ions because the reduction potential of the iron couple is greater than that of copper (+ 0.77 V versus + 0.15 V) (Nakamura et al. 2007). Despite the thermodynamically favorable conditions for reducing ferric ions to ferrous ions rather than to cuprous ions, the reduction of ferric ions under physiological conditions faces limitations. Ferric species (Fe(III) are soluble at lower pH values, but at physiological pH values, they tend to precipitate as Fe(III)-oxy species. Consequently, in the presence of reductants such as ascorbic acid, both precipitation and reduction of Fe(III) species occur (Gulcin and Alwasel 2025). For this reason, in the context of amyloid-β-mediated ROS production, the role of copper(II)/copper(I) has been the most extensively studied. The interaction of iron with amyloid-β is limited to the Fe(II)-amyloid-β complex, which has been structurally characterized.

Copper forms stable, well-defined complexes with amyloid-β in both oxidation states, + 1 (cuprous) and + 2 (cupric) (Nakamura et al. 2007). In terms of ROS formation, the Cu(II)-amyloid-β complex is more toxic to neurons than is amyloid-β alone; however, it is less toxic than free Cu(II) ions are (toxicity in the order of Cu(II) > Cu(II)-amyloid-β > amyloid-β) (Zou et al. 2002; Perry et al. 2003). This can be explained by the shielding effect of amyloid-β, which consists of the saturation of copper(II) binding sites by the donor atoms of amyloid-β. This can be explained by the observed trend, which shows that the more binding sites of the copper ions are blocked (copper has a maximum of 6 binding sites), the less catalytic activity the copper ions exhibit (Valko et al. 2005). Notably, the amount of free Cu(II) ions in biological systems is very limited and is restricted to less than picomolar concentrations. (Tapiero et al. 2003).

In reactions with ROS or redox-active metals, ascorbic acid can behave either as a prooxidant or an antioxidant (Shen et al. 2021). Ascorbic acid can be readily oxidized by one- or two-electron transfer, resulting in the termination of radical chain reactions. Ascorbic acid is oxidized to ascorbyl radical (Asc^•−^), which is considered a terminal radical of radical reactions and is a measure of oxidative stress in an organism (Kasparová et al. 2005).

The application of EPR spin trapping using α-Phenyl-tert-butylnitrone (PBN) spin trap confirmed the one-electron oxidation of ascorbic acid in the presence of free Cu(II) ions and Cu(II)-amyloid-β (Nakamura et al. 2007). Hydrogen peroxide (H_2_O_2_) is a dichotomy, acting as both a beneficial signaling molecule and a damaging oxidative agent in biological systems. The formation of H_2_O_2_ via a two-electron reduction process via free Cu(II) and Cu(II)-amyloid-β in the presence of biological reductants such as ascorbate has been reported (Huang et al. 1999a, b; Murray et al. 2005).

A recent electrochemistry study combined with the use of Cu, Zn-SOD (SOD1) revealed that the formation of H_2_O_2_ is preceded by the formation of transient superoxide radical anions (O_2_^•−^) (Reybier et al. 2016). These findings confirm that the Cu-amyloid-β-mediated formation of O_2_^•−^ contributes to oxidative stress and neurodegeneration (Balland et al. 2010). The Cu-amyloid-β complex can catalyze the decomposition of H_2_O_2_ via the Fenton reaction, resulting in the formation of hydroxyl radicals (^•^OH) and causing severe neurodegeneration. The mechanism of ROS formation catalyzed by the Cu-amyloid-β complex in the presence of O_2_ and a biological reductant (ascorbate) is outlined below (Valko et al. 2005):

$$\:\mathrm{A}\mathrm{m}\mathrm{y}\mathrm{l}\mathrm{o}\mathrm{i}\mathrm{d}-{\upbeta\:}-\mathrm{C}\mathrm{u}\left(\mathrm{I}\mathrm{I}\right)+\:{\mathrm{A}\mathrm{s}\mathrm{c}\mathrm{H}}^{-}\leftrightarrow\:\mathrm{A}\mathrm{m}\mathrm{y}\mathrm{l}\mathrm{o}\mathrm{i}\mathrm{d}-{\upbeta\:}-\mathrm{C}\mathrm{u}\left(\mathrm{I}\right)+\:{\mathrm{A}\mathrm{s}\mathrm{c}}^{\cdot-}+\:{\mathrm{H}}^{+}\:$$

$$\:\mathrm{A}\mathrm{m}\mathrm{y}\mathrm{l}\mathrm{o}\mathrm{i}\mathrm{d}-{\upbeta\:}-\mathrm{C}\mathrm{u}\left(\mathrm{I}\mathrm{I}\right)+\:{\mathrm{A}\mathrm{s}\mathrm{c}}^{\cdot-}\leftrightarrow\:\mathrm{A}\mathrm{m}\mathrm{y}\mathrm{l}\mathrm{o}\mathrm{i}\mathrm{d}-{\upbeta\:}-\mathrm{C}\mathrm{u}\left(\mathrm{I}\right)+\mathrm{A}\mathrm{s}\mathrm{c}$$

$$ \begin{gathered} {\mathrm{Amyloid}} - {{\beta }} - {\mathrm{Cu}}\left( {\mathrm{I}} \right) + {\mathrm{H}}_{2} {\mathrm{O}}_{2} \leftrightarrow {\mathrm{Amyloid}} - {{\beta }} \hfill \\ - {\mathrm{Cu}}\left( {{\mathrm{II}}} \right) + ~^{ \cdot } {\mathrm{OH}} + {\mathrm{OH}}^{ - } ~~\left( {Fenton\;~reaction} \right) \hfill \\ \end{gathered} $$

$$\:\mathrm{A}\mathrm{m}\mathrm{y}\mathrm{l}\mathrm{o}\mathrm{i}\mathrm{d}-{\upbeta\:}-\mathrm{C}\mathrm{u}\left(\mathrm{I}\right)+{\mathrm{O}}_{2}\leftrightarrow\:\mathrm{A}\mathrm{m}\mathrm{y}\mathrm{l}\mathrm{o}\mathrm{i}\mathrm{d}-{\upbeta\:}-\mathrm{C}\mathrm{u}\left(\mathrm{I}\mathrm{I}\right)+{\mathrm{O}}_{2}^{\cdot-}\:$$

$$\:{2\mathrm{O}}_{2}^{\cdot-}+\:2{\mathrm{H}}^{+}\:\to\:\:{\mathrm{H}}_{2}{\mathrm{O}}_{2}+{\mathrm{O}}_{2}\:\:$$

In the Fenton reaction, the amyloid-β-Cu(I) complex catalyzes the heterolytic decomposition of hydrogen peroxide, resulting in the formation of ^•^OH, which causes severe neurotoxicity.

As shown by the set of chemical reactions, copper(II) bound to amyloid-β alternates between cupric (II) and cuprous (I) oxidation states. The coordination environments of Cu(I) and Cu(II) differ significantly, ranging from linear with two nitrogen bonds (for cuprous Cu(I) complexes) to distorted square pyramidal with three nitrogen atoms and one oxygen atom in a distorted equatorial plane, supplemented by a carboxylate oxygen (-COO^−^). This forms a square pyramidal structure in cupric(II) complexes. Because the geometries around the Cu(I) and Cu(II) ions are markedly different (linear versus square pyramidal), the cyclic oxidation and reduction processes involve overcoming a substantial reorganization (activation) energy barrier. It has been suggested that the electron transfer reaction between Cu(I) and Cu(II) amyloid-β complexes occurs via a low-population, redox-favorable state characterized by low reorganization energy due to the considerable structural similarity between the Cu(II) and Cu(I) coordination environments (Balland et al. 2010; Furlan et al. 2012; La Penna et al. 2013). It is estimated that the population of this transient, redox-favorable state accounts for approximately 1/1000 of all complexes in solution (Butterfield et al. 2001).

Methionine sulfoxide reductase A (MsrA) is an antioxidant enzyme that restores oxidatively damaged proteins and free amino acids by converting methionine-S-sulfoxide (MetSO) back into methionine. This enzyme is essential for cellular defense against oxidative stress, a factor associated with aging and neurodegenerative diseases (Moskovitz et al. 2021). An increased level of oxidized amyloid-β proteins depletes methionine sulfoxide reductase A, which in turn fosters the formation of methionine sulfoxide (MetSO) in proteins (Moskovitz et al. 2016). Further studies are necessary to evaluate the interactions among amyloid-β-[MetSO], neuronal cell death, and mitochondria.

### Amyloid-β aggregation

Amyloid-beta (Aβ) aggregation is a key pathology in Alzheimer’s disease and involves a conformational change from a random coil or alpha-helix to a beta-strand structure, which then undergoes conformational transitions into beta-sheets. These beta-sheets tend to aggregate, forming oligomers, protofibrils, and ultimately amyloid plaques that are associated with the toxic effects of protein aggregates (Borghesani et al. 2018). Factors such as the amyloid-β concentration, the presence of redox and nonredox metals, pH, temperature, and other molecules significantly influence the aggregation process. While higher concentrations of amyloid-β peptides promote aggregation, temperature and pH notably affect the kinetics and stability of the aggregates. Lipids may also promote aggregation.

The process of aggregation begins with primary nucleation, which involves the direct self-assembly of monomeric structures into an initial fibril structure, represented by small nuclei that serve as seeds for further fibril growth. This process is slow and requires overcoming a high energy barrier (Arutyunyan et al. 2025). While homogeneous nucleation can occur in the bulk solution, heterogeneous nucleation takes place at the interface. Nuclei can grow by adding more amyloid-β monomers to their ends, a process known as elongation. Elongation occurs more rapidly than primary nucleation. The effects of Cu(II) and Zn(II) on amyloid-β aggregation were studied using full-length amyloid-β peptides (1–40/42). However, the results are ambiguous, mainly because of the complex character of the aggregation process, especially the nucleation phase, which depends on several variables, such as the presence of charged species (ionic strength), pH, the metal ratio, and other parameters (Faller et al. 2013; Viles 2012; Stewart et al. 2017). Stoichiometric amounts of copper(II) and zinc(II) influence amyloid-β aggregation differently; copper forms small protofibrils, whereas zinc leads to the formation of undefined aggregates.

**Fig. 3:**
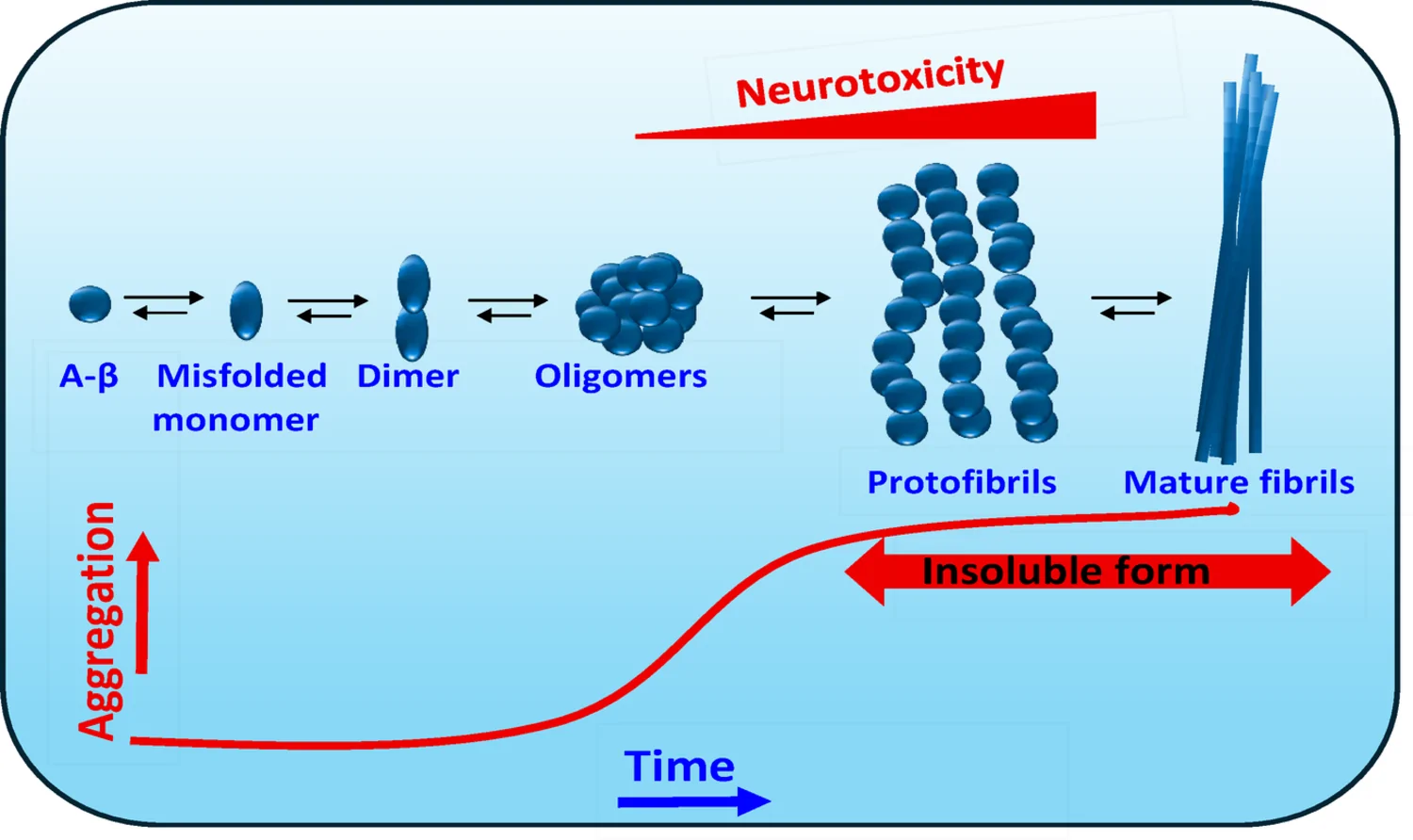
Conversion of amyloid β (Aβ) monomers to oligomers, protofibrils and fibrils

Monomer-dependent secondary nucleation is a crucial step in amyloid-β aggregation and is also relevant to pathological proteins involved in neurodegenerative diseases (Törnquist et al. 2018). The kinetics of secondary nucleation are influenced by hydrophobic, electrostatic, and van der Waals interactions between amyloid proteins and their environment. Its thermodynamics differ significantly from those of primary nucleation and elongation. Secondary nucleation and elongation occur at different sites on a fibril; they occur along the sides of fibrils, whereas elongation occurs at their ends. Therefore, in primary nucleation, elongation involves the addition of monomers to existing fibrils, whereas secondary nucleation creates new fibril ends that then contribute to elongation (Fig. 3).

### Cholinergic hypothesis in AD

The cholinergic hypothesis in Alzheimer’s disease was proposed in 1976 (Davies et al. 1976). The primary chemical reaction involves the synthesis of acetylcholine (ACh) by the enzyme choline acetyltransferase (ChAT) from acetyl-CoA and choline. Acetylcholine is an important neurotransmitter for memory, learning, and cognitive functions. Following its release into the synaptic cleft, acetylcholine binds to postsynaptic receptors (nicotinic or muscarinic) to transmit signals, after which it is rapidly broken down by the enzyme acetylcholinesterase (AChE) into choline and acetate. The degradation of acetylcholine is a crucial step for proper neurotransmission. The cholinergic hypothesis of AD posits that deficient acetylcholine levels result from the degeneration of cholinergic neurons. The main brain areas with reduced acetylcholine levels are the neocortex, hippocampus, and basal forebrain; the striatum and amygdala may also be affected.

The cholinergic hypothesis is closely linked to oxidative stress, which overwhelms the antioxidant defense system and causes neuronal damage, with lipid peroxidation being a key process in this damage. Lipid peroxidation involves a series of ROS-mediated oxidative reactions that degrade lipids, producing malondialdehyde and 4-hydroxynonenal, both of which are toxic to cholinergic neurons. Neurotoxicity manifests as disrupted memory and decreased cognitive function (Ittner et al. 2018). The degeneration of cholinergic neurons, driven by oxidative stress, leads to a decrease in acetylcholine levels. Additionally, there is a notable reduction in the expression of choline acetyltransferase, an enzyme essential for acetylcholine synthesis, which worsens synaptic dysfunction.

A “two-hit hypothesis” proposed that, in addition to oxidative stress in AD, other factors, such as infections or genetic predispositions, act synergistically with cholinergic deficits to drive the progression of Alzheimer’s disease (Zhu et al. 2004a, b).

Acetylcholinesterase inhibitors (AChEIs) can mitigate cognitive deficits in patients with Alzheimer’s disease by preventing the breakdown of acetylcholine. Current AD therapy is based on the application of acetylcholine inhibitors, which increase cholinergic transmission but exhibit mild therapeutic effects (Gajendra et al. 2024). Acetylcholine inhibitors have been in use for more than three decades since the FDA’s approval of tacrine 30 years ago; however, they are no longer used because of hepatotoxicity. Currently used inhibitors are donepezil, rivastigmine, and galantamine (Pratap et al. 2017).

### Acetylcholinesterase and amyloid-β interaction

Acetylcholinesterase is a globular G4 tetramer that consists of 537 amino acids, has a distinct ellipsoidal shape, and is one of the most efficient enzymes known in nature (Walczak-Nowicka et al. 2021). The catalytic site of acetylcholinesterase is composed of two subsites: the esteratic site, containing the catalytic triad serine203, histidine447, and glutamate334 (CAT) (where acetylcholine is hydrolyzed), and the peripheral anionic site (PAS), located at the entrance of the gorge and involved in the movement of acetylcholine to the active site.

The synaptic asymmetric form of AChE can directly interact with amyloid-β and promote amyloid-β fibril formation (De Ferrari et al. 2001). The AChE-amyloid-β complex has more neurotoxic effects than does aggregated amyloid-β alone, and AChE has been shown to be involved in the neurodegenerative changes observed in AD brains through increased Aβ deposition. Amyloid-β preferentially interacts with AChE through hydrophobic interactions, inducing small structural changes in the enzyme. Both the peripheral anionic site (PAS) and the N-terminal region of AChE may play a role in amyloid formation.

In terms of the AChE structure, as Alzheimer’s disease progresses, a transition from the globular G4 tetramer to the G1 monomer structure in the cerebrospinal fluid and cortex has been observed, and the compactness of amyloid-β deposits in the cerebral cortex correlates with increased G1 monomers (Jean et al. 2019). As AD progresses, the G4 form is reduced or lost in favor of the G1 monomer in the cortex and cerebrospinal fluid (CSF) (Jean et al. 2019). The increase in the G1 form correlates with the density of amyloid deposits in the cerebral cortex.

### Neurofibrillary degeneration

Microtubules are structural and functional components of axons, and tau proteins influence their dynamics and spatial organization (Barbier et al. 2019; Khan et al. 2020a, b). The tau protein has six isoforms and belongs to the microtubule-associated protein family, whose functions are regulated mainly by phosphorylation (Tabeshmehr et al. 2023). The tau protein helps stabilize microtubules, which form the internal skeleton of neurons, acting as a crucial transport system within the cell. Each isoform has four parts: (i) the N-terminal domain is predominantly negatively charged and contains two negatively charged inserts, N1 and N2; (ii) a positively charged proline-rich domain; (iii) the microtubule-binding repeat domain (MTBR), crucial for stabilizing microtubules and their abnormal aggregation in neurodegenerative diseases; and (iv) the C-terminal domain involved in microtubule polymerization (Rawat et al. 2022). Under physiological conditions, the tau protein is soluble and flexible, with multiple regions of varying net charge, which creates multiple possibilities for interactions predominantly with charged molecular targets in the cytosol (Mandelkow et al. 2012).

The serine and threonine residues in the proline-rich domain of tau proteins are primary targets for protein kinases and play crucial roles in cell signaling. Their phosphorylation by various kinases, such as Fyn kinase, glycogen synthase kinase-3β (GSK3β), and cyclin-dependent kinase-5 (CDK5), can alter the binding affinity of tau for the microtubule-binding domain. Several phosphorylation sites linked to Alzheimer’s disease are located within the proline-rich region, highlighting the role of phosphorylation in microtubule assembly and the dysfunction observed in Alzheimer’s disease. Abnormally phosphorylated tau in Alzheimer’s disease detaches from microtubules and forms neurofibrillary tangles (Crews et al. 2010). This leads to disruptions in synaptic transmission, axonal transport, premature cell death, cognitive decline, and overall neuronal dysfunction.

Tau has more than 80 potential phosphorylation sites, which occur at serine, threonine, and tyrosine residues. The increased phosphorylation of tau under pathological conditions reduces its affinity for microtubules, resulting in cytoskeleton destabilization (Guo et al. 2017). Hyperphosphorylation of tau is related to amyloid-β, mitochondrial dysfunction, oxidative stress caused by redox metals, and increased levels of kinases such as CDK5 and GSK3β (Huang et al. 2019). The core cause of hyperphosphorylation is an imbalance between kinases that add phosphate groups to tau (the phosphorylation process) and phosphatases that remove them (dephosphorylation), favoring phosphorylation. Additionally, advanced glycation end products (AGEs)—molecules formed from the nonenzymatic reaction of reducing sugars with proteins, lipids, and nucleic acids—are known to promote tau hyperphosphorylation (Li et al. 2012). AGEs can induce tau hyperphosphorylation through RAGE-mediated activation of GSK-3. Targeting the RAGE/GSK-3 pathway with a RAGE antibody has shown promise in improving histopathological changes and memory decline associated with Alzheimer’s disease.

The link between amyloid-β and tau protein in the development of Alzheimer’s disease has been associated with tau hyperphosphorylation via endolysosomal leakage (Gao et al. 2025). These findings indicate that amyloid-β accumulation and aggregation in neurons may cause endolysosomal leakage and the release of asparaginyl endopeptidase, a cysteine protease, which then triggers tau hyperphosphorylation. Inhibition of this enzyme’s activity resulted in suppressed tau hyperphosphorylation.

Tau oligomers are composed mainly of monomeric or dimeric subunits of the tau protein. They may undergo conformational changes, resulting in the formation of stacked beta-sheet strands and ultimately in the formation of insoluble paired helical filaments, which can evolve into large neurofibrillary tangles (NFTs) (Cárdenas-Aguayo Mdel et al. 2014). The process of the formation of insoluble filaments, which accumulate as neurofibrillary tangles, is driven by the hyperphosphorylation of tau.

Oxidative stress activates mitogen-activated protein kinases (MAPKs), including extracellular signal-regulated kinase (ERK) and p38 MAPK, which are ubiquitous serine/threonine glycogen synthase kinase-3 (GSK3) that play a central role in regulating glucose metabolism and insulin signaling and have the potential to phosphorylate tau and form neurofilaments (Zhu et al. 2000, 2001; Reynolds et al. 2000; Butterfield and Halliwell 2019). In vitro experiments confirmed that oxidized fatty acids generated by oxidative stress induce tau polymerization (Gamblin et al. 2000) and that tau aggregation is mediated by the cytosolic adaptive response induced by mitochondrial stress (Samluk et al. 2022; Stamer et al. 2002).

The overexpression of tau has been shown to disrupt mitochondrial function in both cellular and animal models (Esteras et al. 2017a, b; Zhu et al. 2004a, b; Rodríguez-Martín et al. 2016). These findings suggest that mitochondrial dysfunction may be linked to tau protein accumulation. Tau is involved in transporting mitochondria along the neuronal axis and interacting with mitochondrial proteins, which negatively impacts mitochondrial bioenergetics and dynamics (Tracy et al. 2022). Pathological tau reduces complex I activity, resulting in decreased ATP production, loss of mitochondrial membrane potential (ΔΨm), and increased mitochondrial fission and fragmentation.

While many studies have explored the link between oxidative stress and tau pathogenesis, the exact role of oxidative stress in tau pathology remains debated (Bartolome et al. 2022). Oxidative stress can be both a cause and a result of various diseases, playing a complex, bidirectional role in human health (Reddy 2023). The complexity and ambiguity of oxidative stress-related diseases highlight the lack of proven causation (Juránek and Bezek 2005). Researchers have gathered data on tau pathology and examined whether oxidative stress acts as a trigger for tau pathology or if it simply results from the abnormal buildup of tau protein. Multiple studies suggest that oxidative stress initiates tau pathogenesis, whereas others propose that it is a consequence or byproduct of tau protein aggregation.

One line of evidence indicates that posttranslational modifications such as phosphorylation, glycation, methylation, oxidation, nitration, and other mechanisms are crucial in tau pathogenesis (Wang et al. 2020; Nunomura et al. 2001a, b; Dias-Santagata et al. 2007). These structural modifications of tau, which occur mainly in cysteine and methionine residues, are directly linked to the accumulation of ROS; consequently, increased oxidative stress is responsible for tau pathogenesis and occurs before its aggregation (Melov et al. 2007a, b). Unlike the previous model, some studies have suggested that abnormal extracellular tau aggregation, mainly in an insoluble form, can trigger increased ROS production and lead to oxidative stress (Esteras et al. 2021). These tau aggregates activate NADPH oxidase, which further increases oxidative stress and ultimately results in neuronal death (Esteras et al. 2021). The interaction of fragmented tau protein with cupric ions promotes its reduction to cuprous ions and increases oxidative stress via copper redox cycling, leading to the production of hydrogen peroxide, hydroxyl radicals, and other damaging ROS. Consistent with these findings, pathological tau not only exacerbates oxidative stress but also increases neuronal vulnerability to oxidant species. The indirect influence of oxidative stress on tau pathogenesis is mediated by mitochondrial dysfunction of the enzyme Mn-SOD (SOD2), leading to increased tau phosphorylation.

Taken together, the exact interplay between oxidative stress and tau pathogenesis is unclear and requires further work. The positive relationship between increased oxidative stress and tau pathogenesis can be explained by the posttranslational modification of tau protein by kinases and phosphatases. Mitochondrial dysfunction, which is typical for individuals with Alzheimer’s disease and results in excessive production of ROS, has been documented in individuals with tau pathologies (Wang et al. 2020; Esteras et al. 2017a, b). Increased oxidative stress is a significant factor in the initiation of abnormal tau phosphorylation, which subsequently leads to the disruption of tau normal function and contributes to tau pathogenesis (Melov et al. 2007a, b). Conversely, the buildup of abnormal tau protein forms may lead to mitochondrial injury and, consequently, the production of ROS-induced oxidative stress. It has been proposed that a positive feedback loop exists, wherein oxidative stress generated during the initial phases of the disease triggers tau pathology, which in turn exacerbates mitochondrial dysfunction and further oxidative stress, ultimately resulting in neuronal cell death (Ibanez-Salazar et al. 2017).

### Neuroinflammation

Multiple studies have confirmed the pathological activation of both the innate and adaptive immune responses in Alzheimer’s disease. The specific immune response of the brain involves neuroinflammation, which is characterized by the release of inflammatory molecules by specialized brain cells, such as microglia and astrocytes. Genome-related studies have shown a direct link between genetic predisposition to inflammation and the development of the disease (Gatz et al. 2006). Inherited genetic factors contribute to disease progression by an average of 66% (Ridge et al. 2016).

Microglia are a type of glial cell that functions as an immune effector in the central nervous system and produces various immune mediators, such as chemokines, cytokines, inflammasomes, and ROS (Heneka et al. 2025). Microglia are involved in both the silent and symptomatic stages of the disease. Topographical studies, which examine the relationships between anatomical structures and brain functions related to clinical symptoms, have revealed a topographical relationship between microglia and amyloid-β and p-tau but not between amyloid-β and p-tau. This highlights the importance of activated microglia, which express the protein marker CD68, which is associated with phagocytic activity, and macrophage scavenger receptor A (CD 204), a receptor family member on macrophages. Therefore, activated microglia, which are typical in the early stage of AD, influence the morphology of amyloid-β and the progression of the disease.

Amyloid-β (Aβ) fibrils are known to activate surrounding microglia, which in turn release immune mediators, including reactive oxygen species (ROS), chemokines, cytokines, and nitric oxide (^•^NO), as representative RNS. Microglia can phagocytose amyloid-beta plaques, a process characterized by engulfment and attempts to digest them (Gabandé-Rodríguez et al. 2020). However, this microglial clearance mechanism is inefficient, leading to the accumulation of these cells and exacerbating neuroinflammation and neurodegeneration. One of the most significant risk factors for late-onset Alzheimer’s disease, which influences phagocytosis, amyloid-beta accumulation, and neuroinflammation, is complement receptor 1 (CR 1) (Andrews et al. 2023).

Microglia, along with other macrophages, exhibit significant epigenetic diversity and flexibility across various tissues (Troutman et al. 2021). The significant phenotypic flexibility of microglia exposed to various environmental factors (such as pathogens, neuronal damage, stress, and aging) results in alterations in chromatin architecture. These stimuli lead to histone posttranslational modifications (PTMs), including acetylation and methylation, and DNA methylation, thereby transforming chromatin from an open (euchromatin) state to a condensed (heterochromatin) state, thereby activating or repressing gene expression programs, especially those related to inflammatory responses and phagocytosis. Different stimuli can modify histone changes, including acetylation, methylation, and alterations in chromatin structure, which can make the chromatin either more open (euchromatin) or condensed (heterochromatin). The expression and proteostasis of microglia are likely regulated primarily by microRNAs (miRNAs), whose changes in biopsies from patients may predict disease progression (Ansari et al. 2019). While epigenetic alterations can persist, sometimes over several generations, precisely targeting epigenetic mechanisms may reverse these effects.

In addition to microglia, neuroinflammation in Alzheimer’s disease is driven by astrocytes, which are star-shaped and highly abundant cells that are essential for nervous system development and many other functions (Verkhratsky and Nedergaard 2018). The activity of astrocytes has been linked to the inflammatory response of microglia, pathological proteins such as amyloid-β and tau, and various disease indicators (Kozlova et al. 2025). Reactive astrocytes are activated in response to toxic elements, such as amyloid-beta plaques. This activation involves cellular hypertrophy, increased expression of cytoskeletal proteins such as glial fibrillary acidic protein (GFAP) and vimentin, along with many components of cholesterol synthesis and other molecular and functional changes (Hol and Pekny 2015). Although reactive astrocytes can clear waste and secrete neurotrophic factors, they are also linked to harmful effects, including the disruption of synaptic function and the promotion of a proinflammatory environment (Verkhratsky et al. 2023). Reactive astrocytes suppress phagocytosis and thus glutamate uptake and may release neurotoxins. They tend to surround amyloid plaques and increase GABA synthesis, which diminishes neuronal excitability, impairs cognition, and elevates H_2_O_2_ production (Liddelow et al. 2017). Elevated oxidative stress results from increased H_2_O_2_ and ROS, combined with a decrease in antioxidant defenses. Cytokines secreted by astrocytes drive the inflammatory aspects of this disease. Activated microglia can promote a subtype of proinflammatory reactive astrocytes, known as A1, by releasing IL-1α, TNF, and C1q, which are components of the complement system. A1 astrocytes have a reduced ability to support neuronal survival and engage in phagocytosis, leading to neuronal death (Liddelow et al. 2017). The interaction between neurotoxic astrocytes and neural synapses leads to synaptic disconnection, particularly in the hippocampus, resulting in memory impairment. Neuronal degeneration is further exacerbated by toxic saturated lipids secreted by reactive astrocytes (Guttenplan et al. 2021). Knocking out the crucial enzyme for the synthesis of saturated lipids significantly diminishes astrocyte-mediated toxicity in vitro. A diverse group of myeloid cells derived from the bone marrow, including neutrophils and monocytes, play a vital role in the innate immune system by combating infections and promoting inflammation. The accumulation of these cells in Alzheimer’s disease models aggravates disease pathology, and their transient depletion can restore cognitive function and reduce neuropathology (Zenaro et al. 2015). Neutrophils attach to and spread within brain venules, migrate into the parenchyma, and are often accompanied by extracellular fibers, such as neutrophil extracellular traps. They are characterized by high reactivity and the release of cytotoxic molecules such as IL-17, elastase—a serine protease enzyme, and myeloperoxidase—a key enzyme involved in inflammatory and neurodegenerative processes (Gellhaar et al. 2017). Abnormal neutrophil activity is correlated with faster cognitive decline (Dong et al. 2018). In Alzheimer’s disease (AD), damage-associated molecular patterns (DAMPs), such as misfolded proteins (amyloid-beta and tau), heat shock proteins (HSPs), and mitochondrial components (ATP and cytochrome c), activate immune mediators, including proinflammatory cytokines (IL-1β, IL-6, and TNF-α), chemokines, and growth factors (Heneka et al. 2015). These mediators, which are released following the activation of pattern recognition receptors (PRRs), induce a chronic neuroinflammatory response that contributes to Alzheimer’s disease pathology. The release of proinflammatory cytokines, such as IL-1β, involves the formation of gasdermin D-formed pores. IL-1β can promote the phosphorylation of tau, leading to the formation of neurofibrillary tangles. Additionally, neurons activated during the immune response hinder the production of neurotrophic factors vital for normal nervous system function, which is partly compensated for by the release of other factors, such as TNF-α, IL-6, ROS, and RNS—in particular, nitric oxide (^•^NO), which causes axonal and neuronal damage.

### Approved medications to relieve symptoms and treat Alzheimer’s disease

The majority of drugs approved by the FDA are most effective for individuals in the early to middle stages of Alzheimer’s disease.

**Cholinesterase inhibitors.** Cognitive and behavioral symptoms can be controlled to some extent by the application of cholinesterase inhibitors, including galantamine, benzgalantamine, rivastigmine, and donepezil (Sharma 2019). The mechanism of action of cholinesterase inhibitors involves the suppression of the breakdown of acetylcholine, a neurotransmitter crucial for memory and cognitive function. Galantamine prevents the breakdown of acetylcholine and stimulates nicotinic acetylcholine receptors that respond to the neurotransmitter acetylcholine. While donepezil prevents the breakdown of acetylcholine, rivastigmine prevents the breakdown of both acetylcholine and butyrylcholine (Rodda et al. 2009). In the advanced stage of Alzheimer’s disease, the brain produces limited amounts of acetylcholine, and inhibitors lose their effectiveness.

**NMDA antagonist.** The moderate to severe stage of the disease is treated with an N-methyl-D-aspartate (NMDA) antagonist (memantine), which, in practical life, helps people maintain, to some extent, their daily basic activities (Kabir et al. 2019). This medication positively regulates glutamate, the most abundant excitatory neurotransmitter in the brain, which is responsible for memory, learning, and other cognitive functions. Excess glutamate may cause neuronal cell death, and an NMDA antagonist blocks the toxic effects resulting from excess glutamate. Because NMDA agonists and cholinesterase inhibitors act on different targets, they can be used simultaneously. The combined use of memantine and donepezil for moderate to severe Alzheimer’s disease has been demonstrated.

**Immunotherapy.** Immunotherapy-based drugs target the protein amyloid-beta and reduce amyloid plaques, a major hallmark of the disease (Rafii and Aisen 2025). These drugs are effective in the early stages of the disease, when they have been shown to slow cognitive decline, which correlates with reduced amyloid levels in the brain. This group of drugs involves donanemab and lecanemab, and their prescription usually precedes analysis of cerebrospinal fluid or a PET scan to confirm the presence of amyloid deposits. Since side effects may include, among other events, bleeding into the brain, patients should undergo regular MRI examinations.

### Antioxidants and potential antioxidant therapy in Alzheimer’s disease

As a logical consequence of increased markers of oxidative stress in Alzheimer’s disease, there is evidence indicating a reduced antioxidant capacity within the brain, cerebrospinal fluid (CSF), and blood. Optimizing the dose and timing of natural antioxidants may be a widely adopted strategy for preventing or delaying the onset of Alzheimer’s disease. The combination of natural antioxidants with available pharmacotherapies may represent a synergistic application with enhanced overall efficacy in combatting symptoms of Alzheimer’s disease (Jomova et al. 2024).

### Glutathione-related enzymes

One of the most efficient low-molecular-weight antioxidants in the brain is reduced monomeric glutathione (GSH). GSH interacts with a variety of ROS to form oxidized glutathione disulfide (GS-SG). This reaction is catalyzed by glutathione peroxidase (GPx) (Jomova et al. 2024).

$$2\mathrm{G}\mathrm{S}\mathrm{H}\:+\:\:{\mathrm{H}}_{2}{\mathrm{O}}_{2}\:\:\underrightarrow{\mathrm{G}\mathrm{P}\mathrm{x}}\:\:\:\:\mathrm{G}\mathrm{S}-\mathrm{S}\mathrm{G}+2{\mathrm{H}}_{2}\mathrm{O}$$.

Oxidized GS-SG can be reduced back to its reduced form, GSH, by the enzyme glutathione reductase (GR)

$$\:\mathrm{G}\mathrm{S}-\mathrm{S}\mathrm{G}\:+\:\mathrm{N}\mathrm{A}\mathrm{D}\mathrm{P}\mathrm{H}+\:{\mathrm{H}}^{+}\:\underrightarrow{\mathrm{G}\mathrm{R}}\:\:\:2\mathrm{G}\mathrm{S}\mathrm{H}+{\mathrm{N}\mathrm{A}\mathrm{D}\mathrm{P}}^{+}$$

Increased oxidative stress is usually accompanied by a decreased ratio of reduced-to-oxidized glutathione (GSH/GS-SG) (Persson et al. 2014).

As discussed above, the GSH/GSSG ratio within cells is controlled by two primary enzymes, namely, GPx and GR. An increased GSSG/GSH ratio was observed in the hippocampus of patients with mild cognitive impairment and in lymphocytes from Alzheimer’s disease patients (Sultana et al. 2008).

Proton magnetic resonance spectroscopy (¹H-MRS), in conjunction with psychiatric and neuropsychological assessments (including memory, learning, and executive functions), and medical evaluations were utilized to assess the level of glutathione in the anterior and posterior cingulate of patients with mild cognitive impairment (Duffy et al. 2014). Paradoxically, the MRI data revealed increased levels of reduced glutathione (GSH) in the cingulate cortex in patients compared with healthy controls. Increased levels of GSH have been associated with poorer neuropsychological performance, predominantly executive functions. This finding was attributed to a compensatory neuroprotective response to oxidative stress, making the glutathione level a suitable marker for cognitive decline (Persson et al. 2014).

In agreement with the findings of this study, the levels and protein activity of glutathione peroxidase (GPx) and the levels of glutathione reductase (GR) in the hippocampus of patients with mild cognitive impairment compared with controls were evaluated (Sultana et al. 2008). The results revealed no significant alterations in GPx activity, whereas a significant increase in the expression of GR with no change in its reducing activity toward GSSG was observed. The observed data were used to hypothesize that oxidative stress could be an initial factor in the transition from mild cognitive impairment to Alzheimer’s disease.

Selenium is an essential trace mineral that plays a crucial role in mitigating oxidative stress by serving as a vital element in antioxidant enzymes, especially glutathione peroxidase (GPx), which is incorporated into the enzyme as a selenocysteine amino acid. A group of 1389 subjects (aged 60–71) were examined during a nine-year longitudinal study to determine whether decreasing selenium levels are associated with cognitive decline (Akbaraly et al. 2007). The outcome of this trial was that the increased probability of cognitive decline is associated with decreased levels of plasma selenium. The higher the level of selenium is, the lower the degree of cognitive decline.

Thioredoxin (Trx) is a type of small redox protein, and thioredoxin reductase (TR) is the enzyme that helps reduce oxidized Trx, playing protective roles in reducing the degree of cytotoxicity caused by ROS. The levels of Trx protein and the activity of thioredoxin reductase (TR) in the brains of individuals with Alzheimer’s disease were compared to those in the brains of control subjects (Lovell et al. 2000). This research also examined the potential protective effects of thioredoxin and thioredoxin reductase against amyloid-β peptide toxicity in neuronal cultures. A reduction in thioredoxin levels was observed in the hippocampus/parahippocampal gyrus of AD patients (68% of controls) and in the amygdala (59% of controls), two key regions heavily affected by oxidative stress and neurodegeneration. Notably, thioredoxin reductase activity was elevated across all analyzed AD brain regions, with the cerebellum 65% above the control and the amygdala 38% above the control. Although the cerebellum in the AD brain revealed a significantly increased level of thioredoxin reductase compared with that in the control brain, the absolute values of thioredoxin reductase in the AD cerebellum and control samples were lower than those in any other brain region. This likely reflects an upregulation of the antioxidant system in response to increased oxidative stress.

### Superoxide dismutase

SOD together with GPx represents a unique tandem in the defense of an organism against oxidative stress. Numerous trials have reported a connection between SOD activity and cognitive performance; however, the findings are inconsistent (Berr et al. 2004; Sánchez-Rodríguez et al. 2006).

It has been proposed that the SOD/GPx ratio is an indicator of antioxidant capacity and that the equilibrium between these two enzymes is an important determinant of the oxidative stress level associated with cognitive decline and Alzheimer’s disease (Berr et al. 2004). The activities of two key antioxidant enzymes, Cu, Zn-SOD (SOD1), and GPx, were measured in red blood cells from subjects with cognitive decline. The results revealed that cognitive decline was linked to reduced activity of Gpx and increased activity of the enzyme Cu, Zn-SOD. The fluctuations in the Cu, Zn-SOD/GPx ratio indicated that the balance between these two enzymes is associated with cognitive decline. The observed data led to the hypothesis that oxidative stress may serve as an initial factor in the progression from mild cognitive impairment to Alzheimer’s disease.

In a prospective study involving adults aged 62–72 years, higher SOD activity was linked to diminished cognitive performance (Berr et al. 2004), whereas this correlation was not found in older participants in another study (Sánchez-Rodríguez et al. 2006).

A study in which the median age of the total participants was 81 (52.4% women, 47.6% men) was designed to examine the association between the activity of SOD in plasma and cognitive decline in the studied group (Sun et al. 2019). The results revealed that the association between plasma SOD activity and the risk of cognitive decline exhibited a nonlinear dependence; the risk increased with increasing SOD activity at lower levels but stabilized at relatively elevated values.

This study focused on mechanisms that explain the increased risk of cognitive decline linked to increased SOD activity in plasma. SOD expression levels are typically useful markers for examining brain functions (Sun et al. 2019). Overexpressed Cu, Zn-SOD (SOD1) can potentially impair cognitive functions by overproducing hydrogen peroxide, which can then alter the redox environment, disrupt physiological signaling, and inhibit the function of the glutamate-gated ion channel N-methyl-D-aspartate (NMDAR) receptor, a key player in synaptic transmission, learning, and memory (Kumar and Foster 2013).

### Catalase

Among all antioxidant enzymes, catalase has the highest turnover rate and is capable of converting millions of H_2_O_2_ molecules into H_2_O and O_2_ each second (Jomova et al. 2024). In patients with Alzheimer’s disease, catalase is prone to nonenzymatic glycation, leading to enzyme inactivation, accelerated amyloid-β aggregation, mitochondrial dysfunction, increased oxidative stress, and neuroinflammation (Alhumaydhi et al. 2025). Additionally, glycated catalase is more susceptible to degradation, further weakening the antioxidant network.

Catalase may have both a direct and indirect link with the pathology of Alzheimer’s disease. The direct link can be explained by the proposal that amyloid-β directly binds to catalase, which was confirmed by decreased enzyme activity, accumulated hydrogen peroxide, and increased signs of oxidative stress in neuroblastoma and hippocampal neurons (Mattson et al. 1995; Varadarajan et al. 2000). Independent of this mechanism, amyloid-β interacts with Cu^2+^ ions, and this complex can result in the production of H_2_O_2_ (Huang et al. 1999a, b). Thus, these two independent mechanisms lead to deactivation of catalase activity.

In type 1 and 2 diabetic mouse models, the link between catalase activity and diabetes mellitus has been confirmed (Nandi et al. 2019). This is evidenced by a 60-fold increase in catalase activity, which significantly reduces symptoms of diabetic cardiomyopathy and improves cardiomyocyte function. (Ye et al. 2004).

Myo-inositol is a sugar with many health-beneficial properties, including roles in insulin signaling and glucose uptake (Chhetri 2019). Myo-inositol plays a significant role in synapse formation and brain development, and its nonphysiological levels are linked to neurological and psychiatric conditions. Myo-inositol may act as a specific inhibitory ligand for catalase, thereby inhibiting catalase during the presymptomatic phases of Alzheimer’s disease (Ali et al. 2022). Thus, another piece of the mosaic of the impaired antioxidant defense system is associated with elevated levels of myo-inositol in the brain. Myo-inositol reduces turnover and increases the Michaelis‒Menten constant of catalase, resulting in increased H_2_O_2_ levels, which subsequently increase neuronal cell death. In addition, myo-inositol distorts the disk-shaped heme group of catalase, thereby disrupting its structure and stability. Thus, targeting myo-inositol levels may represent a promising therapeutic intervention.

### Vitamin E

In addition to alterations in the levels of antioxidant enzymes, Alzheimer’s disease and cognitive impairment are characterized by alterations in the levels of low-molecular-weight antioxidants. Vitamin E (α-tocopherol) is an effective antioxidant that acts in a lipid environment, providing protection against lipid peroxidation and preserving the integrity of cell membranes (Jomova et al. 2024). Vitamin E encompasses various fat-soluble compounds found in plants, which can be categorized into tocopherols and tocotrienols. Each class comprises four α-, β-, γ-, and δ- homologs (Jiang et al. 2014). Vitamin E (α-tocopherol, α-TOH) reacts with ROS primarily through a hydrogen atom transfer reaction, resulting in a nonradical product and a vitamin E radical (α-tocopheroxyl, α-TO^•^) (Gugliandolo et al. 2017). This radical can subsequently interact with another radical to form a stable product, engage with lipids, or react with a reducing agent such as vitamin C or ubiquinol to restore vitamin E. Vitamin E has a unique role in the central nervous system. Its concentration varies in different brain regions, and the half-life in the brain is shorter than that in other organs (Ulatowski et al. 2014).

The level of vitamin E has been found to be lower in the plasma and CSF of patients with early-stage AD and in the plasma of patients with mild cognitive impairment than in that of controls (Baldeiras et al. 2008). Interestingly, high levels of vitamin E have been reported in a group of elderly people with a reduced risk of AD (Mangialasche et al. 2010). Deficiency of vitamin E is responsible for increased lipid peroxidation, which causes degeneration of neurons, particularly in the cerebellum, as confirmed by high levels of protein nitrosylation. Cellular atrophy and dendritic branching of neurons have been linked to cognitive impairments, which can be suppressed by vitamin E supplementation.

Studies employing rat embryonic hippocampal neurons have proposed that vitamin E can mitigate amyloid-β-mediated oxidative stress (Yatin et al. 2000), as evidenced by inhibited protein oxidation, reduced ROS generation, and reduced neurotoxicity. Another study revealed elevated levels of 4-hydroxy 2-nonenal (HNE), a toxic aldehyde product of the lipid peroxidation process, and thiobarbituric acid reactive substances (TBARS), a bioproduct of lipid peroxidation (Tamagno et al. 2003). These harmful products of the lipid peroxidation process are responsible for caspase-3 activation, cytochrome c release, the cleavage of enzymes responsible for maintaining genomic stability, and poly-ADP ribose polymerase (PARP), which are important mechanisms leading to apoptosis. α-tocopherol showed the ability to counteract these effects.

Vitamin E not only acts as an antioxidant but also may affect the expression of genes associated with Alzheimer’s disease. Rats fed a vitamin E-deficient diet presented diminished hippocampal expression of nerve growth factor (NGF), the best-characterized member of the neurotrophin family of proteins (Rota et al. 2005). In addition, genes associated with the dopaminergic system were negatively affected by vitamin E deficiency. Specifically, APP binding protein member 1 was suppressed, and the Na-dependent dopamine transporter and the D3 dopamine receptor were downregulated.

An Alzheimer’s disease-like phenotype transgenic mouse model (Tg2567) was used to examine the effect of vitamin E administered either prior to or following the deposition of amyloid-β plaques (Sung et al. 2004). One cohort of mice received vitamin E from 5 months to 12 months of age, whereas the other cohort started receiving vitamin E at 14 months of age, which continued until 20 months of age. A sensitive and specific marker of in vivo lipid peroxidation, 8,12-iso-iPF2α-VI, was also evaluated. The level of this marker was significantly lower in both studied groups of mice than in the placebo group. Compared with the placebo group, the vitamin E group presented notable decreases in amyloid-β levels and accumulation, whereas the group supplied at a later age did not exhibit significant decreases in either marker. These results suggest that the timing of vitamin E therapy is crucial and yields the most significant benefit in the early stages of disease progression.

Vitamin E has beneficial effects not only on amyloid-β but also on protein tau hyperphosphorylation, another important hallmark of Alzheimer’s disease. Transgenic mice that overexpress human tau isoforms and are fed α-tocopherol exhibit suppressed or delayed development of tau pathology, which is associated with attenuated motor weakness (Nakashima et al. 2004).

Oxidative stress-mediated dysregulation of signaling pathways leading to tau hyperphosphorylation was studied in cultured neurons. The incubation of neurons with amyloid-β peptide triggered the activation of p38 MAPK, which led to tau hyperphosphorylation (Giraldo et al. 2014). Preincubation of neurons with a water-soluble analog of vitamin E, Trolox, prevented amyloid-β-induced tau phosphorylation. This study supports the idea that antioxidants prevent the oxidative stress-induced hyperphosphorylation of the protein tau in Alzheimer’s disease.

The use of vitamin E has been the subject of several clinical trials (Lloret et al. 2019). In a double-blind study, patients with mild cognitive impairment received daily vitamin E 2000 IU, donepezil 10 mg, or placebo for 3 years (Petersen et al. 2005). The results confirmed that no notable differences were observed in the progression rate to Alzheimer’s disease between the vitamin E and placebo groups. Thus, vitamin E did not provide any advantages for patients experiencing mild cognitive impairment.

A double-blind, 5-year placebo-controlled, parallel-group, randomized clinical trial involving 613 patients with mild to moderate Alzheimer’s disease was conducted (Dysken et al. 2014). Patients received either vitamin E (2000 IU/day) or memantine (20 mg/day), a combination of both substances, or a placebo. Compared with a placebo, a daily dosage of 2000 IU of vitamin E led to a reduction in the rate of functional decline. No notable differences were observed between the groups treated with memantine alone and those receiving memantine in conjunction with vitamin E. These results indicate that alpha tocopherol alone may provide advantages for individuals with mild to moderate AD by decelerating functional decline.

### Vitamin C

Together with vitamin E, ascorbic acid (vitamin C) is another highly effective antioxidant that functions primarily in the plasma. Vitamin C (ascorbic acid, ascorbate anion, AscH^−^) is a water-soluble vitamin with several important properties. It serves as a powerful antioxidant and is vital for the synthesis of collagen, L-carnitine, and certain neurotransmitters; vitamin C is also involved in the absorption of iron and protein metabolism (Jomova et al. 2024; Covarrubias-Pinto et al. 2015). Ascorbic acid acts as a cofactor in neurotransmitter synthesis and affects synaptic neurotransmission; therefore, it can be considered a neuromodulator of neural transmission (Todd and Bauer 1988; Majewska et al. 1990). Ascorbic acid is involved in the catecholamine cascade and participates in the conversion of dopamine to noradrenaline. In addition, it modulates the activity of glutamate receptors and ionic channels, such as calcium channels and GABA receptors (Fan et al. 1999; Calero et al. 2011).

Reports indicate that vitamin C levels are decreased in the plasma of both Alzheimer’s disease patients and mild cognitive impairment patients (Rinaldi et al. 2003). This reduction has been linked to increased oxidative stress, which is typical of neurodegenerative diseases.

Ascorbic acid is present in the reduced form of the ascorbate anion (AscH^−^) under physiological conditions. The favorable standard redox potential of vitamin C (+ 0.08 V) indicates a strong reducing ability, which is capable of donating electrons and acting as a powerful antioxidant in reactions with ROS (R^•^).

$$\:{\mathrm{A}\mathrm{s}\mathrm{c}\mathrm{H}}^{-}+\:{\mathrm{R}}^\cdot{}\to\:{\mathrm{A}\mathrm{s}\mathrm{c}}^{\cdot-}+\mathrm{R}\mathrm{H}$$

Vitamin C (AscH^−^) can regenerate oxidized vitamin E (α-Toc-O^•^) back to its reduced form (α-Toc-OH) because it has a higher standard redox potential than vitamin C (+ 0.37 V), which means that vitamin E is a weaker reducing agent.

$$\:{\mathrm{A}\mathrm{s}\mathrm{c}\mathrm{H}}^{-}+\:{{\upalpha\:}-\mathrm{T}\mathrm{o}\mathrm{c}-\mathrm{O}}^\cdot{}\to\:{\mathrm{A}\mathrm{s}\mathrm{c}}^{\cdot-}+{\upalpha\:}-\mathrm{T}\mathrm{o}\mathrm{c}-\mathrm{O}\mathrm{H}$$

The effect of vitamin C on the formation of amyloid-β plaques was studied in a mouse model of Alzheimer’s disease (Murakami et al. 2011). The reduced formation of most neurotoxic forms of amyloid oligomers was associated with reduced levels of oxidative damage and a decreased ratio of amyloid-42 to amyloid-40, a sensitive marker of disease progression. However, amyloid-β plaque deposition was not suppressed. In addition, the phosphorylation of tau at Ser396 was restored. Thus, ascorbic acid can provide some protection against Alzheimer’s disease pathologies (Monacelli et al. 2017).

Colchicine is a plant-based alkaloid that can induce oxidative stress-related neuroinflammation and neurodegeneration in Charles-Foster rats (Sil et al. 2016). Neuroinflammation, memory impairment, and the peripheral immune response in the hippocampus induced by colchicine were studied. The administration of 200 and 400 mg of vitamin C per kilogram of body weight prevented neurodegeneration and neuroinflammation and resulted in the recovery of memory impairments. The treatment dose of 600 mg of vitamin C did not show evidence of recovery; instead, it mediated an increase in memory impairments, neuroinflammation, and neurodegeneration. Thus, colchicine-induced ROS formation plays an important role in neurodegeneration and memory impairment, accompanied by peripheral immune responses. Another important observation was that lower doses of vitamin C provide antioxidant protection; however, higher doses exhibit prooxidant behavior.

The effects of vitamin C as a modulator of BBB integrity and mitochondrial morphology have been studied (Kook et al. 2014). Many rodents produce their own vitamin C from glucose through the L-gulonolactone oxidase (Gulo) enzyme. An Alzheimer’s disease mouse model with Gulo knockout (KO) mice, which are unable to synthesize their own vitamin C, was supplemented with either low (0.66 g/l) or high (3.3 g/l) levels of vitamin C. Increased supplementation with vitamin C reduced the amyloid plaque burden in the hippocampus and cortex of Gulo knockout mice. This is manifested by the amelioration of BBB disruption and mitochondrial dysfunction.

The protective role of vitamin C against amyloid-β-induced apoptosis in SH-SY5Y neuroblastoma cells has been studied (Huang et al. 2006). Overnight culture of neuroblastoma cells with amyloid-β induced apoptosis, as assessed by phosphatidylserine externalization, DNA fragmentation, and caspase-3 activity in cell lysates. Pretreatment with vitamin C not only substantially prevented apoptosis but also decreased the rate of amyloid-β formation, suggesting that intracellular ascorbate may prevent amyloid-β-induced toxicity.

Acetylcholinesterase (AChE) has been established as the most effective therapeutic target for alleviating symptoms of Alzheimer’s disease (AD). The effect of ascorbic acid on the cholinergic system has been studied (Dhingra et al. 2006; Travica et al. 2019). The cholinesterase-inhibiting activity of ascorbic acid and other substances was compared with that of the standard, long-lasting, irreversible acetylcholinesterase-inhibiting drug metrifonate (trichlorfon). Ascorbic acid (60 mg/kg) and metrifonate (50 mg/kg) were intraperitoneally administered to young male Swiss albino mice for 3 successive days. Compared with the control condition, ascorbic acid significantly suppressed acetylcholinesterase activity.

The levels of vitamin C and other low-molecular-weight antioxidants in the blood were measured in 74 individuals with mild cognitive impairment compared with 158 sex-matched controls (von Arnim et al. 2012). The vitamin C (and β-carotene) levels were significantly lower than those in the control individuals. These data were adjusted for several factors, such as body mass index, smoking habits, and supplement intake. No associations were detected for the blood levels of vitamin E, lycopene, or coenzyme Q10. This study revealed a link between vitamin C (and β-carotene) levels and dementia.

A cohort of 4504 adults over 60 years old with no cognitive impairment was evaluated (1988–1994) for serum vitamin C levels and followed until 2019 for mortality (Appiah et al. 2024). The results, adjusted for several factors, revealed that higher serum vitamin C levels are associated with slower progression of Alzheimer’s disease; however, vitamin C levels above the reference range are linked to an increased risk of AD mortality.

A cross-sectional study of 74 mildly demented individuals compared with 158 matched controls was selected to evaluate the levels of vitamin C, vitamin E, carotenoids, and coenzyme Q10 (von Arnim et al. 2012). The blood levels of vitamin C and beta-carotene were significantly decreased in the investigated groups of individuals, even after adjustment for many characteristics. No correlations were found for vitamin E, lycopene, or coenzyme Q10.

A positive trial included 5395 participants aged 55 years or older who were free of dementia, had reliable dietary assessments, and were monitored for 6 years (Engelhart et al. 2002). After the follow-up period, 197 participants (3.6%) were diagnosed with dementia, of whom 146 (2.7%) were diagnosed with Alzheimer’s disease. After adjusting for many factors, such as carotid plaque occurrence, education, alcohol intake, smoking, and the use of antioxidant supplements, the intake of vitamin C (121.6 mg per day) and vitamin E (13.8 mg per day) was associated with a decreased risk of Alzheimer’s disease. Since oxidative reactions in dementia and Alzheimer’s disease involve the development of atheromatous plaques in the arteries, increased intake of antioxidants, including vitamin C, could decrease the risk of dementia by reducing the risk of atherosclerosis.

Cerebrovascular pathology, particularly endothelial dysfunction, is a primary driver of vascular dementia, a form of cognitive decline caused by damaged blood vessels in the brain (Bomboi et al. 2010). Recent research has revealed an association between cerebrovascular pathologies and Alzheimer’s disease (Snowdon et al. 1997). A study involving 1015 participants revealed that the occurrence of silent strokes was more than double the risk of developing Alzheimer’s disease (Vermeer et al. 2003). Individuals with elevated blood pressure presented a greater risk for vascular dementia and AD. This finding suggests that the stiffness of arteries and atherosclerosis represent a significant predisposition for the development of AD.

Endothelial dysfunction and breakdown of the blood‒brain barrier (BBB) are observed during ischemia‒reperfusion events, with diminished bioactivity of nitric oxide (NO^•^) proposed as a critical factor in tissue damage during reperfusion (Tsao et al. 1990). The nitric oxide synthesized by the endothelium is recognized for its ability to inhibit various pathogenic processes associated with atherosclerosis. Several mechanisms involving vitamin C have been proposed to mitigate endothelial dysfunction via the generation or metabolism of nitric oxide. Vitamin C inhibits endothelial dysfunction by preventing the oxidation of low-density lipoprotein (LDL), which is known to increase the permeability of the vascular endothelium (Kaneko et al. 1993). In addition, vitamin C may promote the release of nitric oxide from S-nitrosothiols present in the plasma and may reduce nitrite (NO_2_^−^) into NO^•^, potentially preserving its levels in tissues or plasma (Millar 1995). Vitamin C is also a cofactor in the regulation of endothelial NO synthase, leading to increased NO^•^ production.

### Flavonoids

Flavonoids are plant-derived polyphenols that naturally occur in fruits, vegetables, honey, tea, and wine. Flavonoids exhibit various beneficial health effects related to their anti-inflammatory, antimicrobial, antiviral, antioxidant, and anticancer properties (Rebas 2025). Unlike other low-molecular-weight antioxidants, flavonoids, in addition to their antioxidant effects, have the ability to influence signaling pathways. Flavonoids possess a 15-carbon skeleton characterized by two benzene rings (designated A and B) interconnected by a three-carbon bridge, along with an oxygen-containing heterocyclic C ring (the C6-C3-C6 backbone) (Jomova et al. 2025). Notable structural features include the presence of hydroxyl (-OH) groups, a 2,3-double bond within the C ring for numerous subclasses, and the positioning of the B ring relative to the C ring, which determines the classification of the flavonoid into distinct subgroups.

Flavonoids have the capacity to modify the mechanism of action of many neurotransmitters; however, their bioavailability is rather low, mainly due to poor absorption, extensive metabolism, and rapid excretion (Rebas et al. 2020). Flavonoids can cross the blood–brain barrier (BBB), which is essential for their therapeutic benefits in neurological disorders. The relatively low toxicity of flavonoids makes them safer alternatives to synthetic drugs for treating neurodegenerative diseases. However, the accumulation of these bioactive compounds in the brain may induce certain toxic reactions, including prooxidant behavior, especially in the presence of redox-active metals. Owing to their low toxicity, flavonoids are considered safer alternatives to the currently used synthetic medications for treating neurodegenerative disease, which may have many adverse side effects.

Dysfunction of autophagy, which is responsible for the degradation of damaged cellular components, has been associated with neurodegenerative disorders, such as Alzheimer’s disease, Parkinson’s disease, and amyotrophic lateral sclerosis, which are characterized by the formation of toxic aggregates of misfolded proteins (Minocha et al. 2022; Guo et al. 2018; Nixon and Rubinsztein 2024). Conversely, persistent autophagy activation in the later stages of neurodegenerative disease can lead to neuronal cell death. Flavonoids show promising therapeutic potential for affecting autophagy signaling in neurodegenerative disorders.

Genistein (5,7-dihydroxy-3-(4-hydroxyphenyl)-4 H-1-benzopyran-4-one) is a naturally occurring phytoestrogen found mainly in soybeans. High doses of genistein (150 mg/kg/day) activate the autophagy mechanism in a streptozotocin-induced rat model of the sporadic form of Alzheimer’s disease (Pierzynowska et al. 2019). The dose used led to complete degradation of the β-amyloid and hyperphosphorylated tau proteins in the brain. Experiments using cell cultures have shown that these effects require stimulated autophagy. Therefore, genistein interferes with the autophagy-dependent process, which helps alleviate Alzheimer’s disease symptoms.

Quercetin (2-(3,4-dihydroxyphenyl)-3,5,7-trihydroxy-4 H-1-benzopyran-4-one) is one of the most studied flavonoids. Quercetin has been shown to inhibit the aggregation of amyloid-β and related paralysis by activating macroautophagy and proteasomal degradation pathways (Zaplatic et al. 2019). The inhibitory activity of quercetin against amyloid-β is related to its ability to inhibit BACE1-induced APP cleavage at the β-site, preventing amyloid-β formation (Suganthy et al. 2016).

In addition to amyloid plaques and tau tangles, two key hallmarks of Alzheimer’s disease, a notable decrease in acetylcholine (ACh) levels, along with the degeneration of cholinergic neurons, represents a crucial neurochemical characteristic that plays a significant role in the memory and cognitive decline associated with the disease (Minocha et al. 2022). Flavonoids show acetylcholine inhibitory activity, which makes them promising compounds for the development of multitargeted drugs against Alzheimer’s disease. This activity of flavonoids leads to an increase in acetylcholine and facilitates the transmission of impulses at synaptic junctions (Bachman et al. 1992; Uriarte-Pueyo and Calvo 2011). The number of flavonoids with acetylcholinesterase inhibitory activity sharply increased with time. In 2006, only 16 flavonoids were reported to have acetylcholinesterase inhibitory activity; 5 years later, in 2011, 128 flavonoids were reported (Barbosa Filho et al. 2006). Some reports have proposed that both the existence of a 4´-OMe group and a 7-O-sugar moiety are necessary for the inhibition of AChE, and other reports have indicated the importance of other substituents at different positions (Khan et al. 2009). In general, flavanones, which contain a saturated C ring, demonstrate a somewhat lower level of AChE inhibitory activity than flavones, which contain a double bond between carbons C-2 and C-3. This suggests that the presence of a double bond at C2‒C3 carbons may also be crucial. The most profound inhibitory activity of a series of flavonoids against AChE has been reported for quercetin (76%), followed by genistein (66%), luteolin-7-O-rutinoside (55%), and silibinin (51%) (Orhan et al. 2007).

Flavonoids mitigate neuroinflammation through the inhibitory activity of various proinflammatory molecules, such as nuclear factor kappa-light chain enhancer of activated B cells (NF-κB), activator protein-1 (AP-1), interleukin-1beta (IL-1β), tumor necrosis factor-alpha (TNF-α), IL-6, and IL-8 (Al-Khayri et al. 2022). Flavonoids can also inhibit proinflammatory enzymes such as lipoxygenase (LOX), cyclooxygenase-2 (COX2), and inducible nitric oxide synthase (iNOS). Flavonols, including quercetin, kaempferol, morin, and myricetin, have been identified as more effective lipoxygenase inhibitors than flavones (Serafini et al. 2010). Conversely, flavanones such as naringenin demonstrated no efficacy. In macrophages treated with lipopolysaccharide (LPS) or cytokines, quercetin, apigenin, and luteolin were shown to inhibit nitric oxide (•NO) production (Kim et al. 1998).

Animal studies have confirmed that the neuroprotective effects of flavonoids are associated with improved cognitive function in patients with neurodegenerative diseases. The impact and mechanism of baicalein on synaptic function in an Alzheimer’s disease model (APP/PS1 mice) were examined (Gu et al. 2016). Using behavioral, electrophysiological, and biochemical methods, researchers have shown that baicalein reduces the impairment of hippocampal long-term potentiation (a process in which synapses become more effective after high-frequency electrical stimulation) caused by amyloid-β oligomers through the PI3K signaling pathway. Additionally, prolonged oral treatment with baicalein suppressed 12/15-lipoxygenase (LOX) and glycogen synthase kinase 3 beta (GSK3β) activity, which was linked to decreased levels of β-site APP cleaving enzyme 1 (BACE1) and amyloid-β production, prevented tau protein phosphorylation, and restored NMDAR-dependent long-term potentiation. All these beneficial effects were confirmed by improved memory performance in the transgenic mice.

Flavonoids are potentially effective substances for addressing dysregulated metal homeostasis, which is one of the hallmarks of Alzheimer’s disease (Chib et al. 2025). These molecules contain, in addition to a keto group (ring C), several hydroxyl groups (rings A, B, and C) capable of chelating redox-active copper and iron, as well as nonredox zinc (Jomova et al. 2022). Flavonoid ligands consist of small, nonpolarizable atoms such as oxygen and nitrogen and are therefore considered hard bases. They possess a strong affinity for hard acids, such as copper, iron, and zinc ions, which are characterized by low polarizability and carry a charge. Metal chelation depends on the pK values of the flavonoid functional groups; lower pK values enhance their ability to interact with metal ions. The involvement of the 5-hydroxy group and 4-keto group in metal ion interactions can lead to the formation of stable five- or six-membered rings (Simunkova et al. 2021). The complexation and oxidation of quercetin and luteolin with specific metal cations—namely, Cr(III), Mn(II), Fe(III), Co(II), Ni(II), Cu(II), Zn(II), and Al(III)—were studied in aqueous solutions at pH values of 4 and 7.4 (Malacaria et al. 2023). Techniques such as UV‒Vis spectroscopy, RP-UHPLC-PDA-MS, ESI-MS, and ^1^H NMR provided insights into the complexing capacity, stoichiometry, and preferred metal binding sites of these materials. At a physiological pH (7.4), both luteolin and quercetin formed complexes with all the tested metal ions, whereas at a nonphysiological pH of 4, the flavonoids formed complexes with only trivalent metal cations, specifically Cr(III), Fe(III), and Al(III). Targeting flavonoids to combat redox-metal-mediated neurotoxicity through chelation results in a significant reduction in catalytic activity in the Fenton reaction, which results in the damage of hydroxyl radicals.

Several clinical trials evaluating the role of flavonoids in alleviating symptoms of Alzheimer’s disease are currently underway (Kaur et al. 2022). A randomized, double-blind, personalized clinical trial (NCT03978052) with 150 subjects of both sexes is currently being conducted to evaluate subjective cognitive decline following epigallocatechin gallate (EGCG) and multimodal interventions (de la Torre and de Salut Mar 2024).

Clinical studies have evaluated the health effects of supplementation with the natural polyphenol resveratrol (Liu et al. 2025). In a multicenter, phase 2, double-blind, placebo-controlled trial, 179 participants were randomized to receive a placebo or resveratrol for 52 weeks. A subanalysis, conducted on cerebrospinal fluid (CSF) biomarkers, such as neuronal damage, inflammation, and microglial activity, within a specific group of patients who received either a placebo (*n* = 21) or resveratrol (*n* = 30) was reported. Biomarkers associated with neuronal damage, such as neuron-specific enolase and hyperphosphorylated neurofilaments, were reduced. A ubiquitous lysosomal aspartic protease, cathepsin D, which plays a role in proteolytic degradation, cell invasion, and apoptosis, was also reduced in the group supplemented with resveratrol. Conversely, angiogenin, an RNAse-A-family protein that promotes angiogenesis, has been found to be increased in neurodegenerative diseases. The administration of resveratrol led to a significant reduction in the expression of a marker of AD progression, soluble triggering receptor expressed on myeloid cells 2 (TREM2) in the CSF. A decrease in inflammation and damage to various tissues, including matrix metalloprotease (MMP9), was observed.

Matrix metalloproteinase 9 (MMP9) plays a key role in various brain pathologies, including neurodegenerative disorders and neuroinflammation. Through the release of ROS and cytokines, MPP9 influences BBB permeability and other functions. MMP9 can destroy gap junctions in the neurovascular unit, increase CNS permeability, and cause inflammation. Individuals with mild to moderate Alzheimer’s disease (*N* = 119) were orally administered 1 g of the sirtuin 1 (SIRT1) activator resveratrol twice a day over a period of 52 weeks (Moussa et al. 2017). One of the most important findings was the significant decrease in the level of MMP9 in the CSF following resveratrol treatment.

Another study indicated that CSF MMP9 levels are considerably lower in Alzheimer’s disease patients with reduced amyloid-β42 and amyloid-β40 levels, along with elevated total tau and phosphorylated tau (p-tau) levels, than in healthy control subjects (Mroczko et al. 2014). Such differing evidence suggests that further comprehensive research is necessary to reveal the therapeutic effects of resveratrol in clinical settings. The low bioavailability of resveratrol should also be considered (Phukan et al. 2023).

### Carotenoids

Carotenoids are pigments responsible for the yellow, red, and orange colors of various plants, fruits, and vegetables (Jomova and Valko 2013). These natural compounds serve as antioxidants, predominantly by scavenging ROS, including singlet oxygen (^1^O_2_). Some carotenoids, known as provitamin A carotenoids, can be converted in the intestine by an enzyme beta-carotene monooxygenase type 1 (BCMO1) into vitamin A (Flieger et al. 2024). There are more than 1000 naturally occurring carotenoids classified into two main groups: xanthophylls and carotenes. Carotenes have a hydrocarbon structure, and xanthophylls contain specific functional groups, such as -COOH, -C = O, and -OH.

The most prevalent carotenoids in nature include β-carotene, lutein, and lycopene (Crupi et al. 2023). β-Carotene is a red‒orange pigment and the most commonly found carotene. Lutein is a xanthophyll carotenoid present in plants. It serves as an antioxidant crucial for maintaining eye health by protecting against light-induced damage. Lutein is associated with potential contributions to cognitive function and brain development. Zeaxanthin is one of the most common carotenoid xanthophylls found in leafy greens, orange fruits, and vegetables. Like lutein, zeaxanthin also plays a key role in eye health. Lycopene is a red‒orange pigment found in tomatoes and watermelon that functions as a potent antioxidant. Fucoxanthin is an antioxidant and anti-inflammatory pigment from brown seaweed with potential benefits for chronic diseases, including neurodegeneration. Astaxanthin is a red‒orange pigment within xanthophylls characterized by efficient antioxidant properties.

Carotenoids can interfere with several processes associated with the pathogenesis of Alzheimer’s disease, including neurotoxicity, amyloid-β aggregation, disrupted lipid metabolism, neuroinflammation, oxidative stress, and mitochondrial dysfunction (Kabir et al. 2022a, b). Carotenoids can positively modulate antiamyloid-β aggregation mechanisms, amyloid oligomer-mediated signaling, anti-neuroinflammatory effects, hypocholesterolemic mechanisms that reduce blood cholesterol levels, the activation of antioxidant enzymes, and ROS quenching.

Rats subjected to a high-fat diet were given hippocampal infusions of amyloid-β for 14 days to induce memory deficits resembling Alzheimer’s disease (Kim et al. 2024). This group of rats displayed increased deposition of amyloid-β, diminished hippocampal insulin signaling (due to suppressed phosphorylation of protein kinase B), increased neuroinflammation, acetylcholine activity, and memory deficits compared with those in the normal group. Compared with the positive control, lutein and zeaxanthin effectively mitigated memory impairment. Resveratrol inhibited acetylcholinesterase activity, lipid peroxidation, and proinflammatory cytokines (TNF-α and IL-1β), improved hippocampal insulin signaling, and increased the mRNA expression of brain-derived neurotrophic factor (BDNF) and ciliary neurotrophic factor. On the basis of the results of this study, supplementation with lutein and zeaxanthin at doses ranging from 1.5 to 2.0 g/day may have significant implications for preventing the progression of Alzheimer’s disease by mitigating neuroinflammation and sustaining systemic and central glucose homeostasis. Nonetheless, additional clinical trials are necessary to assess their efficacy and safety in human populations.

The protective effects of zeaxanthin on amyloid-β in rats have been studied (Li et al. 2022). Zeaxanthin prevents alterations in the ultrastructure of cerebrovascular tissue and has protective effects on regulating the redox state, inhibiting endothelin-1 levels, and sustaining amyloid-β homeostasis in both the plasma and cerebrovascular systems. Additionally, the dysregulated expression of receptor for advanced glycation end products (RAGE), a signaling protein receptor-related protein 1 (LRP-1), and the potent proinflammatory cytokine interleukin-1 beta (IL-1β), which are all induced by amyloid-β, was mitigated through pretreatment with zeaxanthin. Thus, the cerebrovascular protective effects of zeaxanthin could be linked to a decrease in amyloid-β levels in both the brain and blood circulation, achieved through the activation of the clearance mechanisms of amyloid-β within specific circuit pathways.

Male Wistar rats receive amyloid-β (1–42) via intracerebroventricular injection, which leads to significantly increased oxidative stress, mitochondrial damage, and elevated activity of TNF-α, IL-6, and caspase-3, accompanied by notable memory retention deficits (Prakash et al. 2014). Lycopene administered at doses of 2.5 and 5 mg/kg for three weeks offered protective effects against amyloid-β-induced cognitive impairment, as evidenced by improved memory retention, reduced neuroinflammation, and decreased mitochondrial damage. Thus, animal studies suggest that lycopene may partially reverse amyloidogenesis.

Several other carotenoids, including crocetin, crocin, astaxanthin, and fucoxanthin, are also being examined experimentally for their potential benefits in combating Alzheimer’s disease (Kabir et al. 2022a, b). Fucoxanthin, a brown xanthophyll carotenoid from algae, has various health-promoting properties. Amyloid-β oligomers have been reported to suppress the prosurvival phosphoinositide 3-kinase (PI 3 K)/Akt signaling pathway and activate the proapoptotic ERK pathway in human neuroblastoma SH-SY5Y cells (Lin et al. 2017). Fucoxanthin effectively prevents amyloid-β oligomer-induced neuronal apoptosis and inhibits intracellular ROS formation in a concentration-dependent manner. It also significantly reduces alterations in the PI3K/Akt and ERK pathways caused by amyloid-β oligomers. The neuroprotective effects of fucoxanthin against amyloid-β oligomer-induced neurotoxicity were confirmed by its complete elimination with the PI3K inhibitors LY 294,002 and wortmannin.

Crocetin, a natural carotenoid dicarboxylic acid, has various pharmaceutical effects. It has been shown that crocetin can colocalize with amyloid-β via a process mediated by human serum albumin (HSA) (Ahn et al. 2011). Crocetin inhibits the formation of amyloid-β fibrils, destabilizes existing fibrils, stabilizes amyloid-β oligomers, and prevents their transformation into fibrillar structures. Therefore, crocetin has potential for pharmaceutical applications against Alzheimer’s disease. Pretreatment with crocetin activated extracellular signal-regulated ½ phosphorylation (ERK1/2), which confirmed that crocetin exerts neuroprotective effects against amyloid-β-induced damage in a mouse hippocampal neuronal cell line (HT22).

HT22 cells are subjected to amyloid-β(1–42) for 24 h (Kong et al. 2014). As expected, a significant decrease in cell viability, increased ROS formation, a decrease in the mitochondrial membrane potential, and phosphorylation of extracellular signal-regulated kinase (ERK) were observed. HT22 cells preincubated with crocetin for 24 h followed by amyloid-β (1–42) treatment presented a notable increase in cell viability, reduced ROS formation, and an increase in the mitochondrial membrane potential.

The role of carotenoids in clinical trials of neurodegenerative diseases has been studied only rarely (Flieger et al. 2024). Astaxanthin is a natural, red‒orange pigment from the carotenoid family. The polar nature of astaxanthin makes it an efficient antioxidant that can span biological membranes. Twenty-four healthy young human volunteers (12 men and 12 women, with a mean age of 24.2 ± 0.6 years) and 38 healthy seniors (20 men and 18 women, with a mean age of 56.2 ± 1.0 years) underwent quantitative analysis of amyloid-β40 and amyloid-β42 in human red blood cells via ELISA (Kiko et al. 2012). The results revealed that amyloid-β levels are significantly higher in red blood cells than in plasma and that these levels in erythrocytes increase gradually with age. A significant positive correlation was observed between amyloid-β concentrations and reactive lipid byproducts—phospholipid hydroperoxides (PL-OOHs). Experiments with the carotenoid astaxanthin demonstrated a decrease in amyloid-β concentrations in red blood cells after supplementation.

Within the Rush Memory and Aging Project, 927 participants who were free from Alzheimer’s disease consumed energy-adjusted dietary carotenoids, including β-carotene, α-carotene, lutein-zeaxanthin, lycopene, and β-cryptoxanthin, for a mean of 7 years (Yuan et al. 2021). The neuropathological changes in Alzheimer’s disease in the brain were evaluated through postmortem autopsies conducted on 508 deceased individuals. The consumption of total carotenoids was linked to a significantly reduced risk of Alzheimer’s disease after adjusting for factors such as age, sex, education, ApoE-ε4 status, engagement in cognitively stimulating activities, and levels of physical activity. For specific carotenoids, both lutein-zeaxanthin and lycopene exhibited inverse relationships with overall brain pathology, whereas lutein-zeaxanthin additionally showed inverse correlations with the Alzheimer’s disease diagnostic score.

A total of 44 individuals (average age 72 years) were supplemented for a period of 12 months with either lutein + zeaxanthin (12 mg/d) or placebo (Lindbergh et al. 2018). Neurological functions were evaluated via functional magnetic resonance imaging (fMRI), a noninvasive technique designed for mapping brain activity. Both carotenoids, lutein and zeaxanthin, seemed to mitigate cognitive decline in the verbal learning task. The left dorsolateral prefrontal cortex (important for executive functions) and anterior cingulate cortex (important for integrating cognitive and emotional information) exhibited significant interactions during the learning phase, as confirmed by BOLD fMRI. fMRI BOLD refers to blood oxygen level-dependent functional MRI, a method utilized to assess brain activity through the detection of variations in blood oxygen levels. Thus, supplementation with lutein and zeaxanthin seems to positively influence neurocognitive function by improving cerebral perfusion.

Three experimental trials investigating the impact of supplemental xanthophylls plus omega-3 fatty acids on disease progression in patients with Alzheimer’s disease were conducted (Nolan et al. 2018). Trial 1 consisted of 12 individuals with Alzheimer’s disease (4 mild and 8 moderate) who were supplemented with lutein: meso-zeaxanthin: zeaxanthin at a dosage of 10:10:2 mg/day. Trial 2 consisted of 13 patients with Alzheimer’s disease (2 mild, 10 moderate, and 1 severe) who were supplemented with lutein: meso-zeaxanthin: zeaxanthin 10:10:2 mg/day plus 1 g/day of fish oil. Control Trial 3 consisted of 15 control subjects free of Alzheimer’s disease supplemented with lutein: meso-zeaxanthin: zeaxanthin at a dosage of 10:10:2 mg/day (formula 1) for 6 months. The blood xanthophyll concentration (evaluated by HPLC) was significantly greater for the formulation in Trial 2 than for that in Formulation 1. The progression of Alzheimer’s disease was suppressed in this group, as manifested by improved memory and emotional status. It can be speculated that fish oil probably improves the bioavailability of carotenoids, resulting in positive outcomes for patients with Alzheimer’s disease.

### Ambiguities, challenges, and future directions in antioxidant therapy for Alzheimer’s disease

Ambiguities surrounding antioxidant therapy for Alzheimer’s disease stem from factors such as poor bioavailability, structural changes, interactions with other molecules such as metals, limited transport to the brain, paradoxical pro-oxidant effects at high doses and increased partial pressure of oxygen, and ineffective, nontargeted strategies. Despite strong evidence linking oxidative stress to Alzheimer’s disease, numerous trials have yielded unsatisfactory results. Several challenges related to antioxidant therapy that need further research include the following:

1. Limited bioavailability and delivery: Many antioxidants struggle to reach effective concentrations in the brain, or their levels may be inadequate for protection.
2. Pro-oxidant effects: Some antioxidants, such as vitamins E and C, can switch from an antioxidant role to a pro-oxidant role at high concentrations in the presence of redox-active metals, leading to unexpected outcomes.
3. Lack of specificity: In general, antioxidants, including mitochondria, may not effectively target the specific areas most affected by oxidative stress in Alzheimer’s disease patients.
4. Inadequate dose or duration: Research may be hampered by treatment periods that are too short or doses that are too low to combat the severity of the condition.

Since oxidative stress is recognized as a factor in Alzheimer’s disease progression, antioxidants could be promising adjunct treatments. While dietary antioxidants might help lower risk, and some, such as vitamin E, have shown limited benefits, their use as sole treatments remains uncertain and requires further study. Future approaches may combine antioxidants with advanced delivery systems, such as nanoparticles, to increase their effectiveness, particularly by targeting specific cellular pathways, including those involving mitochondria.

If antioxidants help prevent oxidative damage and slow the progression of AD, they are not considered a cure. Early intervention and a4 holistic approach—including medication, lifestyle changes, and mental stimulation—are generally recommended.

## Parkinson’s disease 

Parkinson’s disease (PD) is one of the most common neurodegenerative movement disorders, with a projected global prevalence of over 25 million by 2050 (Su et al. 2025). The main motor symptoms in patients with Parkinson’s disease include tremor (rhythmic shaking), bradykinesia (slowed movement), postural instability (difficulty maintaining balance), and rigidity (muscle stiffness). Nonmotor features include sleep disorders such as insomnia, a reduced sense of smell, gastrointestinal problems, mood and cognitive changes, voice alterations, drug-induced psychosis, fatigue, urinary issues, and other symptoms. Whereas sensory deficits, gastrointestinal problems (constipation), and mood disorders may manifest years or even decades before the onset of motor symptoms, other symptoms, such as cognitive decline, tend to emerge in the later stages (Dionísio et al. 2021).

The major hallmarks of this severe disorder, as evidenced by neuroimaging and postmortem analysis, include axonal deterioration at an early stage, followed by the loss of energizing dopaminergic neurons located in the substantia nigra pars compacta (SNpc). Additionally, intracellular protein aggregates, primarily composed of abnormally aggregated α-synuclein (α-syn), are observed within neuronal cell bodies and neurites, referred to as Lewy bodies and neurites, respectively, alongside persistent neuroinflammation. Lewy bodies are a hallmark of brain disorders called Lewy body dementias, which include dementia characterized by Lewy bodies and Parkinson’s dementia, characterized by decreased thinking skills.

There are currently no effective therapies that can reverse or slow the progression of the disease, despite efforts to develop various treatment alternatives capable of alleviating the clinical manifestations of the disease. The key therapy for dopamine replacement is based on the use of the dopamine precursor levodopa (L-3,4-dihydroxyphenylalanine) (Poewe et al. 2017).

Symptoms typical of Parkinson’s disease correlate with the advancement of α-syn brain pathology and associated neuronal dysfunction, which has been classified into several stages (Braak et al. 2003). The first two stages are characterized by the formation of α-syn aggregation in the forebrain olfactory bulb and the dorsal motor nucleus of the vagus nerve (DMV), which is located in the medulla oblongata of the brainstem. These stages are likely related to gastrointestinal problems and a reduced sense of smell. The third and fourth stages are characterized by a significant increase in the concentration of aggregated α-syn, whose pathology dominates in the fifth stage and affects the hippocampus, amygdala, and mesocortex, the transitional zone between the allocortex and isocortex.

A key factor in the degeneration of these neurons and neuronal damage is ROS-induced oxidative stress caused by disrupted cellular redox processes (Trist et al. 2019). Oxidative stress damages vital biomolecules, including DNA, proteins, and lipids, which impairs neuronal function and structural integrity (Dionísio et al. 2021). Oxidative stress-mediated damage is a prominent biochemical abnormality during the early stages of the disease, even before significant neuronal loss occurs (Ferrer et al. 2011). Therefore, excessive ROS production is likely a primary driver of dopaminergic neuron degeneration rather than just a secondary effect of neurodegeneration. A deeper understanding of the complex role that oxidative stress plays in PD development could reveal new targets for therapy and early diagnosis.

## Etiology of Parkinson’s disease

### Genetics

The familial form of Parkinson’s disease (10–15% of cases) is associated with mutations in several genes, with key examples being the SNCA gene encoding the α-synuclein protein and the LRRK2 (leucine-rich repeat kinase 2, also known as dardarin) protein for autosomal-dominant forms (Tysnes and Storstein 2017). The PRK (PARK2) gene encoding parkin, the PINK1 (PARK6) gene encoding PTEN-induced kinase 1 (PINK1), the PARK7 gene encoding a protein encoding DJ-1, and ATP13A2 (ATPase cation transporting 13A2), the gene encoding a probable cation-transporting ATPase 13A2, are related to autosomal recessive forms of Parkinson’s disease, in which a person must inherit two copies of a mutated gene (one from each parent) to develop the condition. The sporadic form of Parkinson’s disease accounts for approximately 80–90% of cases and is caused by a combination of genetic susceptibility and environmental factors.

### Lifestyle habits – physical activity, smoking, caffeine

Epidemiological studies suggest that exercise may lower the risk of developing Parkinson’s disease (Bhalsing et al. 2018). Physical activity has the potential to improve both motor and nonmotor symptoms and offers neuroprotective and neurorestorative benefits. The incorporation of physical activity into the treatment plan should consider several patient-specific factors, such as the risk of physical instability, depression, and cognitive dysfunction. While most studies indicate that physical activity benefits motor and nonmotor symptoms in Parkinson’s disease patients, many questions remain (Amara and Memon 2018). These include which type of exercise is most effective in reducing disease risk or alleviating symptoms, as well as how often exercise should be performed to achieve optimal benefits (Kouli et al. 2018).

Cigarette smoking has been extensively studied in the context of Parkinson’s disease. A comprehensive meta-analysis across 20 countries revealed an inverse relationship between smoking and Parkinson’s disease (Hernán et al. 2001, 2022). Additionally, two other meta-analyses reported similar inverse correlations between smoking and PD. The possible mechanism underlying these findings involves nicotine stimulation of dopamine release and the activation of nicotinic acetylcholine receptors (nAChRs) on dopaminergic neurons, which have neuroprotective effects (Ritz et al. 2007; Breckenridge et al. 2016).

The influence of caffeine on Parkinson’s disease (PD) onset has also been explored in several studies. Animal research indicates that caffeine acts as an antagonist of the adenosine A2A receptor, which may confer protective effects against PD (Ross et al. 2000; Chen et al. 2001). Two prospective epidemiological studies (Hernán et al. 2022; Ascherio et al. 2001) have shown a reduced risk of developing Parkinson’s disease among coffee drinkers compared with nondrinkers. Like smoking, the definitive role of caffeine in preventing Parkinson’s disease has not yet been fully established. The inverse relationship between coffee intake and PD incidence appears to be stronger in men than in women. Since estrogen inhibits caffeine metabolism, the effect of caffeine in postmenopausal women should be adjusted considering hormone replacement therapy.

### Environmental factors in PD

Structural similarities between the neurotoxic pesticides paraquat and rotenone prompted epidemiological studies to identify a link between the incidence of Parkinson’s disease and the incidence of these toxins in rural areas and occupational exposure to these toxins (Dauer et al. 2003). The involvement of toxins in Parkinson’s disease has not been confirmed and remains a subject of debate. While these neurotoxins are known to inhibit mitochondrial complex I and lead to ATP depletion and oxidative stress, which can ultimately cause apoptosis and neuroinflammation, their permeability across the blood‒brain barrier is limited, which casts doubts on their importance in the etiology of Parkinson’s disease (Nandipati and Litvan 2016).

### Iron accumulation and redox reactions in the substantia nigra

Iron is essential for proper functioning of the nervous system (Zeng et al. 2024). Iron concentrations are twice as high in the postmortem substantia nigra pars compacta than in age-matched controls (Genoud et al. 2017). In vivo magnetic resonance imaging (MRI) of the proton transverse relaxation times in the SNc confirmed that iron deposition in early Parkinson’s disease patients was spatially restricted to the most vulnerable region, the ventral substantia nigra (vSNc) (Martin et al. 2008).

**Fig. 4:**
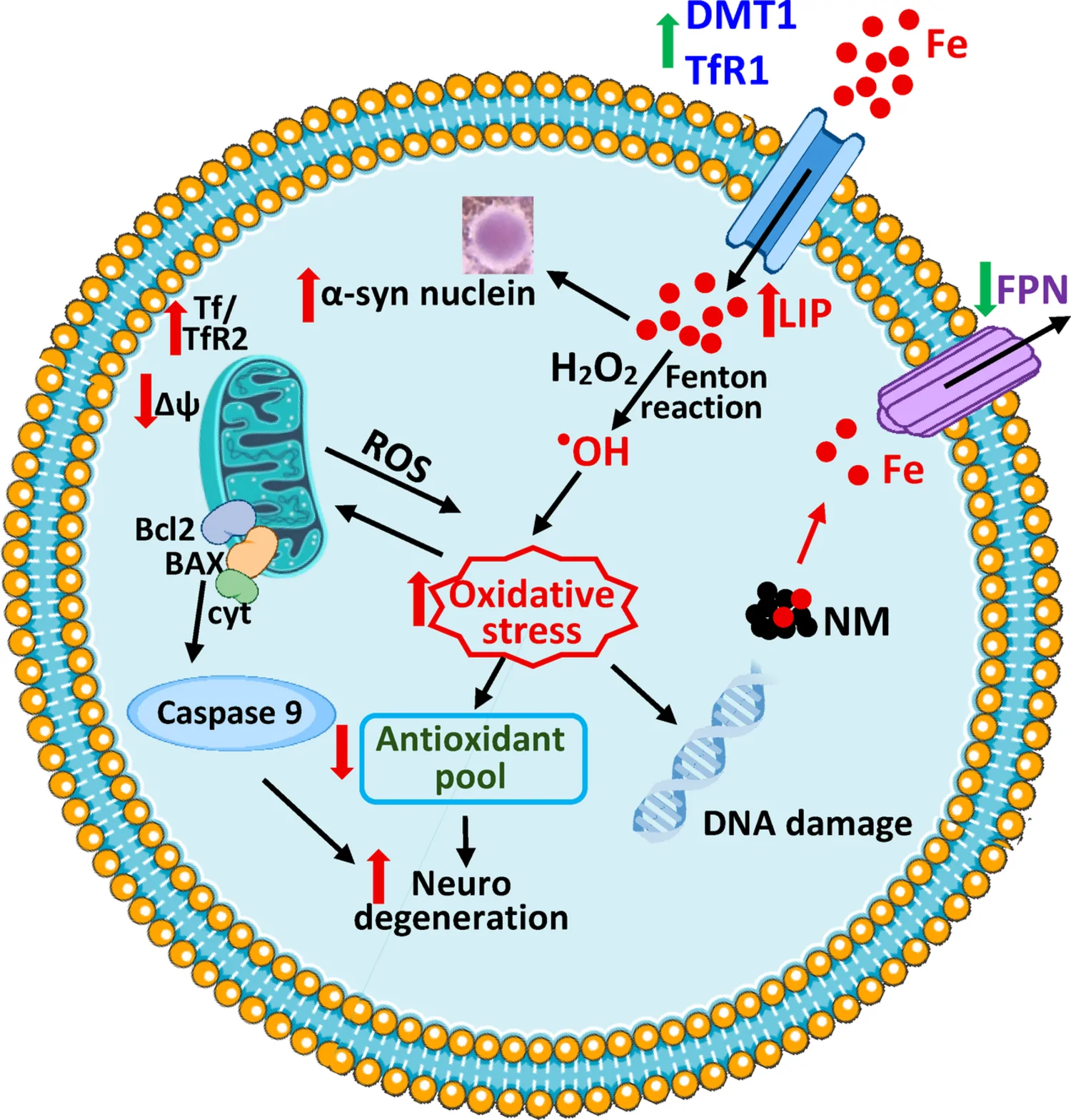
Iron and oxidative stress in Parkinson’s disease

Iron accumulation in the Parkinson’s disease brain is believed to result from a complex interplay among iron homeostasis, neuroinflammation, mitochondrial dysfunction, and other mechanisms (Fig. 4). Some attempts to correlate increased iron consumption during adolescence with the probability of developing Parkinson’s disease at advanced ages have been found to be ambiguous (Logroscino et al. 2008).

Iron homeostasis is achieved by the concerted action of several proteins, such as transferrin (TRF), divalent metal ion transporter 1 (DMT1), iron-regulatory protein (IRP), and ceruloplasmin. A minimal decrease in brain iron levels in individuals with iron deficiency anemia indicates that the blood‒brain barrier may also be involved in cerebral iron homeostasis (Youdim et al. 1989). Dysregulated iron homeostasis, characterized by disturbed transport, circulation, and storage, may result in abnormal iron deposition in the substantia nigra. In addition, dysregulated iron homeostasis may be linked to the age-related dysfunction of ferritin observed in *Caenorhabditis elegans*, where the reactive ferrous ion Fe^2+^ fails to be effectively oxidized into the more chemically stable ferric ion Fe^3+^ for storage (James et al. 2015). Neurons in the substantia nigra have an unusual capacity to store iron; therefore, the role of microglia and astrocytes in the accumulation of iron in this brain region deserves further study.

Mutations in genes related to iron metabolism are often associated with the development of this condition (Hinarejos et al. 2020). The typical characteristic of this condition is the gradual worsening of extrapyramidal symptoms and neurological signs, characterized by considerable clinical and genetic variability.

### Dopamine and redox changes in dopaminergic neurons in the substantia nigra

The substantia nigra is a midbrain structure within the basal ganglia circuitry that is morphologically divided into two regions: the pars compacta (SNpc) and the pars reticulata (SNpr). The SNpc is composed primarily of dopaminergic neurons, which produce the monoamine neurotransmitter dopamine, and is essential for motor control (Sonne et al. 2025). The SNpr is rich in GABAergic neurons, which are the main projection neurons in this structure and produce the inhibitory neurotransmitter gamma-aminobutyric acid (GABA).

Like many other neurodegenerative diseases, age is the main risk factor for Parkinson’s disease. However, the substantia nigra pars compacta, an essential brain region in Parkinson’s disease, is especially susceptible to oxidative damage (Trist et al. 2019). Even in seemingly healthy individuals, postmortem brain studies have shown that this region exhibits some level of age-related cellular damage, which is more pronounced than in other brain areas. This damage is described as “mild” pathological damage, indicating that it exceeds normal aging and is often linked to oxidative stress, potentially serving as a precursor to neurodegenerative diseases (James et al. 2015). The selective vulnerability of certain brain regions, such as the nigral subregion, may be related to mitochondrial dysfunction, dysregulation of nonredox calcium, redox-active metal ions such as iron and copper, and reduced expression of antioxidant enzymes.

A significant shift in Parkinson’s research occurred in 1982, when a group of young people developed irreversible parkinsonism after the injection of synthetic heroin containing lipophilic molecules 1-methyl-4-phenyl-1,2,3,6-tetrahydropyridine (MPTP), which is known to be a potent neurotoxin that targets neurons in the substantia nigra (Ballard et al. 1985; Langston et al. 1983). MPTP crosses the blood‒brain barrier, is metabolized by monoamine oxidase B into 1-methyl-4-phenyl-2,3-dihydropyridinium (MPDP+), and is further oxidized to the toxic metabolite 1-methyl-4-phenylpyridinium (MPP+) (Schildknecht et al. 2015). MPP+ accumulates within dopaminergic neurons, resulting in a dysfunctional electron transport chain (ETC) that manifests as suppressed ATP production; increased levels of ROS, including the superoxide radical anion (O_2_^•−^); and disruption of the membrane potential. Neurodegeneration caused by ROS in dopaminergic neurons can be mitigated by ROS scavengers and inhibitors of inducible nitric oxide synthase (iNOS) (Flanagan et al. 2018).

In addition to mitochondria, other significant sources of ROS, such as the enzymes NADPH oxidases, cytochrome P450, and lipoxygenases, and activated inflammatory glial cells during neuroinflammation, which may significantly contribute to Parkinson’s disease, have been identified (Bardaweel et al. 2018; Schildknecht et al. 2017).

Dopaminergic (DAergic) neurons located in the substantia nigra pars compacta (SNpc) may experience greater oxidative stress than other neurons in the brain because of the presence of dopamine (Dionísio et al. 2021). Dopaminergic neurons release dopamine, and this process is closely linked to oxidative stress because dopamine breakdown can generate ROS. This process interferes with the accumulation of the free iron pool in the substantia nigra and striatum of patients with Parkinson’s disease (Zeng et al. 2024). The metabolism of dopamine by monoamine oxidase produces hydrogen peroxide, which can undergo Fenton-type reactions with ferrous iron (Fe(II) to generate ROS, such as hydroxyl radicals.

Dopamine can oxidize to form electron-deficient dopamine quinone (DAQ), either spontaneously in the presence of redox-active metals or through enzymatic processes (Hauser et al. 2013; Hastings et al. 1996). The dopamine quinone features a partial positive charge primarily situated at the fifth carbon atom of the catechol ring, making it susceptible to nucleophilic attack by thiolate ions found in cysteine residues of both reduced glutathione (GSH) and proteins (Graham et al. 1978). The major product resulting from the interaction between cysteine thiolate and dopamine quinone is 5-cysteinyl-DA, which has been identified in the human SNpc and is significantly elevated in the brains of individuals with Parkinson’s disease (Spencer et al. 1998). Modification of cysteine residues via irreversible covalent interactions between DAQ and cysteine may have a deteriorating effect. The covalent interaction between dopamine and reduced glutathione (GSH) depletes the levels of GSH available in redox reactions, compromising cellular viability (Rabinovic and Hastings 1998).

Dopamine toxicity has been studied in animal models. Rats that received an intramuscular injection of dopamine experienced selective lesions whose extent correlated with the levels of 5-cysteinyl-dopamine and 5-cysteinyl-DOPAC, where DOPAC is 3,4-dihydroxyphenylacetic acid. Under physiological conditions, dopamine is stored within acidic vesicles where it is protected from oxidation and interaction with glutathione and protein thiols. Altered sequestration of dopamine can trigger toxic reactions, as documented in mice that express very low levels of vesicular monoamine transporter 2 (VMAT2) and exhibit a loss of neurons in the substantia nigra pars compacta manifested by motor and nonmotor symptoms, which are typical of Parkinson’s disease (Caudle et al. 2007; Taylor et al. 2011). Another animal model confirmed the capacity of poorly sequestered dopamine to oxidize into reactive dopamine quinones (Chen et al. 2008a, b).

**Fig. 5:**
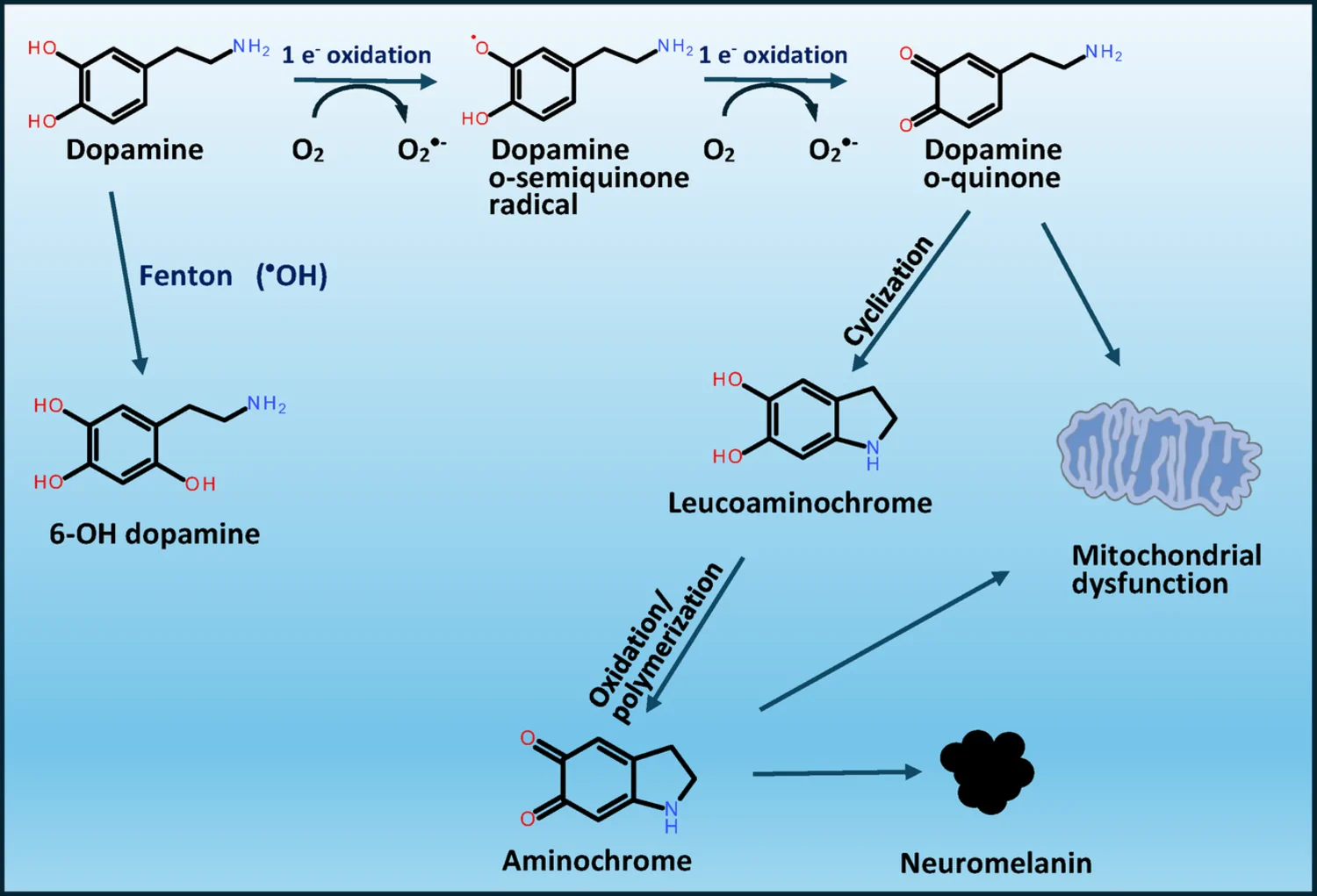
Oxidation of dopamine and Parkinson’s disease

Prooxidant interactions between iron and dopamine are particularly important in the aging substantia nigra pars compacta (SNc) because of the preferential buildup of labile iron in this specific brain region (Hare and Double 2016). Under acidic conditions in neurotransmitter vesicles, dopamine remains stable; however, in the cytosol or extracellular space, where the pH is relatively high, dopamine may rapidly autoxidize into the transient dopamine-semiquinone radical (Simunková et al. 2023) (Fig. 5). This reaction produces superoxide radical anion (O_2_^•−^), which can enter several redox reactions, such as the dismutation reaction by superoxide dismutase (SOD), forming hydrogen peroxide (H_2_O_2_). Iron present in the reduced ferrous state (Fe^2+^) can catalyze the heterolytic decomposition of hydrogen peroxide, resulting in the formation of reactive hydroxyl radicals (^•^OH) (Jomova et al. 2023).

Dopamine can be oxidized to 6-hydroxydopamine (6-OHDA) through a multistep process involving iron ions, hydrogen peroxide, or other oxidants (Jiang et al. 2025). The initial step involves the oxidation of dopamine to its ortho-quinone, which is then further oxidized to 6-OH-DA p-quinone by ROS, particularly in the presence of oxidizing hydrogen peroxide or hydroxyl radicals (Segura-Aguilar et al. 2014). The formation of toxic dopamine-quinone species is relevant to neurodegenerative diseases such as Parkinson’s disease. Dopamine-semiquinone radicals can undergo a series of reactions to form aminochrome, which induces mitochondrial dysfunction by inhibiting complex I, decreasing ATP production, and increasing the production of superoxide radicals (Zhou et al. 2023). Superoxide radicals can reduce ferric ions (Fe^3+^) to ferrous ions (Fe^2+^) and catalyze the Fenton reaction to form highly damaging hydroxyl radicals (Jomova et al. 2023). ROS formation has been confirmed by numerous markers of oxidative damage not only in the substantia nigra but also in the cerebrospinal fluid (CSF) and blood of patients suffering from Parkinson’s disease.

### Mitochondrial dysfunction in PD

Mitochondrial dysfunction is a key hallmark of both idiopathic and genetic Parkinson’s disease (Henrich et al. 2023). Studying familial PD (PARK) genes has greatly advanced our understanding of the causes of PD. Although many PARK genes have been identified, some are directly linked to impaired mitochondrial function and integrity. The most common mutations in autosomal recessive early-onset Parkinson’s disease (AR-EOPD) occur in genes encoding PINK1 (PARK6) and Parkin (PARK2). Both PINK1 and Parkin play vital roles in activating signaling pathways that respond to mitochondrial damage (Ge et al. 2020), such as triggering autophagy and regulating mitochondrial biogenesis, among other processes (Lazarou et al. 2015; Deng et al. 2008). PINK1 is responsible for phosphorylating subunits of NADPH dehydrogenase (complex I), a key enzyme in ATP production. PINK1 knockout mice not only exhibit defective complex I activity but also exhibit altered calcium buffering and impaired mitochondrial membrane potential (Morais et al. 2009; Akundi et al. 2011). Recent studies have linked the redox properties of Parkin to its antioxidant function in midbrain neurons (Tokarew et al. 2021). Mutations in the DJ-1 (PARK7) gene are another cause of mitochondrial-related parkinsonism, which often presents with nonmotor symptoms such as psychosis and cognitive decline (Bonifati et al. 2003).

ATP production from nutrient oxidation involves ROS generation through the electron transport chain (Drose and Brandt 2008). Under normal conditions, approximately 2% of the electrons leak from complexes I and III, reacting with molecular oxygen to form superoxide radicals. To maintain redox balance, mitochondria contain two of the three main antioxidant superoxide dismutases. Mn-SOD (SOD2), found in the mitochondrial matrix and inner membrane, converts superoxide into hydrogen peroxide, which, in the presence of trace redox-active metals such as iron or copper, can produce hydroxyl radicals (^•^OH) via the Fenton reaction (Ruszkiewicz et al. 2015). The other antioxidant enzyme, Cu, Zn-SOD (SOD1), which is located in the mitochondrial intermembrane space, also converts superoxide into hydrogen peroxide and oxygen. Hydrogen peroxide, both a vital signaling molecule under physiological conditions and a damaging species under supraphysiological concentrations (> 100 nM), is converted to water and oxygen by catalase, the enzyme with the highest turnover of all antioxidant enzymes (Sies 2017). An additional strong antioxidant system is represented by glutathione peroxidases (GPxs), which act in mitochondria, including GPx1, GPx4, and GPx2 (Flohé et al. 2022). While GPx1 is found in the cytoplasm and mitochondria, GPx4 plays a major role in protecting mitochondrial function and oxidative phosphorylation, and GPx2 has been shown to preserve mitochondrial function, particularly in aged cells. GPx uses reduced glutathione (GSH) to convert hydrogen peroxide into water.

Altered physiological redox regulation in the healthy aging brain leads to increased mitochondrial ROS production in the substantia nigra (Trist et al. 2019). Genetic mutations, environmental toxins, and other factors typical of Parkinson’s disease significantly worsen the impairment of the electron transport chain and overall prooxidant state in the substantia nigra, as shown by oxidative damage markers (Schapira et al. 1990). In such a pathological state, all contributing factors disrupt the balance between high energy demand and high calcium buffering on one side and electron transport chain dysregulation on the other. Mitochondrial dysfunction is further compounded by environmental toxins (discussed above), which can cross lipid membranes and enter mitochondria, damaging complex I redox activity (Richardson et al. 2005). This results in reduced ATP synthesis and increased superoxide radical production. Neurotoxicity caused by toxins, including MPTP, has been demonstrated in transgenic mice with increased Cu, Zn-SOD activity, which are resistant to dopaminergic neuron damage after MPTP administration (Przedborski et al. 1992).

Dopaminergic neurons are prone to oxidative damage due to hypermetabolism; consequently, prolonged mitochondrial ROS overproduction causes significant harm to these neurons (Lucchesi et al. 2025; Flores-Ponce et al. 2024). Increased iron levels in the substantia nigra of Parkinson’s disease patients can catalyze ROS formation and promote the aggregation of α-synuclein. Mitochondrial complexes I and II contain multiple iron‒sulfur [Fe‒S] clusters essential for their function in the electron transport chain (Simola et al. 2007). Dopamine metabolism itself also produces ROS such as H_2_O_2_, O_2_^•−^, ^•^OH, and dopamine semiquinone radicals in the cytosol and synaptic cleft of neurons (Muñoz et al. 2016). Additionally, the importance of iron extends beyond this role, as disrupted iron homeostasis can impair mitochondrial function, which is linked to disorders such as Parkinson’s disease. Another contributor to mitochondrial dysfunction in Parkinson’s disease is the accumulation of point mutations and deletions in mitochondrial DNA (mtDNA) (Hauser et al. 2013). Mitochondrial DNA, which is circular and derived from bacteria, encodes 13 proteins, 2 rRNAs, and 22 tRNAs, for a total of 37 genes. These 13 proteins are crucial for cellular respiration, especially oxidative phosphorylation. Seven mitochondrial DNA genes—ND1, ND2, ND3, ND4, ND4L, ND5, and ND6—encode subunits of mitochondrial complex I, forming its hydrophobic core within the inner mitochondrial membrane. The remaining subunits of human complex I are encoded by nuclear DNA. Approximately one-quarter of humans carry heteroplasmic mtDNA, with levels associated with age and certain mutations increasing over time, whereas others decrease (Sondheimer et al. 2011). Defects in complex I in Parkinson’s disease are caused by point mutations or deletions in mitochondrial DNA. Histochemical and genetic analyses can identify cells with mtDNA defects; these cells lack cytochrome c oxidase staining but retain succinate dehydrogenase activity, which appears blue. This technique confirmed that neurons lacking cytochrome c oxidase are common in the substantia nigra pars compacta. COX-negative cells, indicating possible deletions in complex I-encoding regions of mtDNA, are frequently found in the brain and heart tissues of PolG mutator mice (Bender et al. 2006). Furthermore, qPCR studies have shown that these mice accumulate mtDNA deletions at a greater rate than do wild-type mice (Vermulst et al. 2008).

### Neuroinflammation in PD

Oxidative stress and inflammation are tightly interconnected, establishing a self-intensifying cycle in which each can initiate and worsen the other (Bej et al. 2024). ROS-mediated oxidative stress stimulates inflammatory responses. In contrast, inflammation triggers the release of inflammatory cells, which generate additional ROS, forming a feedback loop that drives tissue damage and disease progression.

The brain region most affected by neuroinflammation is the substantia nigra pars compacta, which is observed in both familial and sporadic Parkinson’s disease and is predominantly driven by microglia and, to a lesser extent, by astrocytes and oligodendrocytes (Perry 2012). Neuronal damage is predominantly caused by ROS-activated NADPH oxidase (NOX2). The pathological activation of glial cells and oxidative stress form a vicious cycle characterized by a self-perpetuating process in which neurodegeneration and neuroinflammation reinforce one another and drive the progression of the disease (He et al. 2020). The most affected mitochondria are damaged mitochondria, triggering neuroinflammation by releasing “damage-associated molecular patterns” (DAMPs), such as mitochondrial DNA, proteins, and ROS. DAMPs activate pattern recognition receptors (PRRs), triggering an immune response such as inflammation and cell death. DAMPs activate primarily microglia, leading to the release of inflammatory molecules that can cause a self-perpetuating cycle of damage and inflammation.

Hexokinase is a key enzyme in glycolysis and can be inhibited by the natural amino sugar N-acetylglucosamine (NAG) (Wolf et al. 2016). Recent research revealed that bacterial NAG can bind to hexokinase, triggering an immune response by causing the enzyme to dissociate from the mitochondrial outer membrane and release mtDNA, which in turn activates the NLRP3 inflammasome. This finding reveals a dual role for hexokinase, not only in metabolism but also as an innate immune sensor. NLRP3 activates the formation of the proinflammatory cytokine interleukin-1β (IL-1β) (He et al. 2020), which induces mitochondrial damage and ROS production, thus establishing a self-perpetuating toxic feedback loop.

An interesting link between angiotensin, iron homeostasis, and microglia in dopaminergic neurons in Parkinson’s disease has been reported (Garrido-Gil et al. 2013). Angiotensin, through type-1 receptors, influences iron homeostasis in both dopaminergic neurons and microglia, highlighting the significant role of glial cells in the effective regulation of iron homeostasis within dopaminergic neurons.

### Approved medications to relieve symptoms of Parkinson’s disease

The three main categories of medications for Parkinson’s disease include levodopa, dopamine agonists, and monoamine oxidase B inhibitors (MAO-B). All of these effects improve motor symptoms through increased dopamine levels in the brain. Levodopa is an effective medication that is converted in the brain to dopamine. Levodopa is frequently coadministered with competitive inhibitors, aromatic amino acid decarboxylase inhibitors (AAADIs), carbidopa, or benserazide to suppress the side effects of the therapy. Dopamine agonists such as pramipexole or ropinirole can mimic dopamine through stimulation of nerve receptors. MAO-B inhibitors, such as safinamide or rasagiline, preserve the level of dopamine by inhibiting the monoamine oxidase B protein, which breaks down dopamine.

Other supporting medications include catechol-O-methyltransferase (COMT) inhibitors, which are a class of medications that block the COMT enzyme to prolong the effects of levodopa by preventing its breakdown in the body to reach the brain. Anticholinergics are used to treat tremor and rigidity by blocking acetylcholine to repair a chemical imbalance in the brain. Unlike levodopa, dopamine agonists such as pramipexole or rotigotine mimic dopamine effects in the brain. Compared with levodopa, their effects take longer and may be used together to improve treatment efficacy.

Administered medications can be supplemented by deep-brain stimulation (DBS), which is a surgical intervention employed to manage specific symptoms associated with Parkinson’s disease. This effective treatment primarily targets the movement-related symptoms of Parkinson’s disease. In addition, MRI-guided focused ultrasound, commonly referred to as MRgFUS, is a noninvasive therapeutic approach that uses ultrasound to destroy specific regions of tissue and relieve symptoms.

## Oxidative stress-targeted treatment with antioxidants in Parkinson’s disease

### Superoxide dismutases (SODs)

Among the three SOD isoenzymes, Mn-SOD (SOD2) plays the most important role in Parkinson’s disease; however, its exact involvement in Parkinson’s disease is complex. Numerous studies have indicated that the total antioxidant activity of superoxide dismutase is lower in the bloodstream of patients with Parkinson’s disease than in that of healthy individuals. This observation implies dysregulation of antioxidant defense mechanisms. Some studies suggest that defects in SOD activity can be a consequence of the progression of Parkinson’s disease, whereas others point to specific gene polymorphisms in Cu, Zn-SOD (SOD1) and Mn-SOD (SOD2) that may increase the risk of developing PD. Research has also explored the therapeutic potential of SOD and SOD-mimetic compounds in protecting against PD-related neurotoxicity.

SOD activity in the blood and red blood cells of patients with early and advanced Parkinson’s disease was evaluated (Bostantjopoulou et al. 1997). Patients with advanced-stage disease exhibited a significant reduction in SOD activity in both whole blood and erythrocytes. No significant relationship between L-DOPA treatment and SOD activity was found. A decline in SOD activity occurs in Parkinson’s disease patients over time.

Proteomic analysis revealed that cultured rat primary microglia produce and release Cu, Zn-SOD (SOD1) (Polazzi et al. 2013). The neuroprotective role of released SOD1 has emerged from experiments in which primary cerebellar granule neurons (CGNs) were subjected to the dopaminergic toxin 6-hydroxydopamine (6-OHDA). The neuroprotection of cerebellar granule neurons provided by SOD1 was facilitated by increased pools of calcium from external sources. The neuroprotective effect of SOD1 was halted by the application of SOD1 inhibitors.

The activities of the cytosolic and mitochondrial isoforms of superoxide dismutase were studied in the motor cortex of eight patients with Parkinson’s disease and eight healthy control subjects (Radunović et al. 1997). Compared with that in the control group, mitochondrial SOD activity in the motor cortex was significantly increased. These findings suggest an altered antioxidant defense mechanism in upper motor neurons, confirming the role of oxidative stress in the pathogenesis of Parkinson’s disease.

Numerous indications currently underscore the significant role of superoxide radicals in causing neuronal toxicity. Consequently, a novel therapeutic avenue that warrants thorough evaluation is the application of SOD-mimetic molecules, which can selectively eliminate superoxide radicals through a catalytic mechanism (De Lazzari e al. 2018).

Various SOD mimetics have been prepared and tested, although only a limited number of these materials appear to possess therapeutically beneficial properties. SOD mimetics were originally developed to suppress inflammation and oxidative damage; in addition, mimetics have been explored for their capacity to inhibit chronic activation of microglia, which is one of the suggested contributors to disease progression in Parkinson’s disease. Porphyrin-based SOD mimetics and Mn(II)-cyclic polyamine-based SOD mimetics demonstrated highly encouraging characteristics that could be utilized to address Parkinson’s disease (Batinić-Haberle et al. 2010; Salvemini et al. 1999).

### Catalase

Catalase is a prominent antioxidant enzyme with the highest turnover of all antioxidant enzymes, safeguarding cells against the oxidative stress associated with Parkinson’s disease (Jomova et al. 2024). Studies suggest that individuals with PD may exhibit diminished catalase activity and reduced enzyme levels, leading to an accumulation of detrimental hydrogen peroxide and the progression of the disease. In contrast, enhancing catalase levels via therapeutic approaches is currently being investigated as a possible method to mitigate oxidative stress and potentially address Parkinson’s disease.

The mutant α-synuclein protein is recognized for its ability to inhibit both the expression and activity of catalase (Yakunin et al. 2014). The inhibition of catalase activity by α-synuclein likely occurs through the obstruction of peroxisome proliferator-activated receptor γ (PPARγ) transcriptional activity, which is responsible for regulating the expression of the CAT gene (Yakunin et al. 2014). These experimental findings suggest that the reduced catalase activity and elevated hydrogen peroxide production observed in Parkinson’s disease patients may be attributed to the indirect inhibition of catalase expression by the α-synuclein molecule (Nandi et al. 2019).

Glycation is a nonenzymatic process characterized by the covalent binding of reducing sugars to the amino groups arginine and lysine on a protein, resulting in the formation of glycated proteins with impaired function (Alhumaydhi et al. 2025). Glycated catalase undergoes structural changes that suppress its catalytic activity to convert hydrogen peroxide. The impaired function of glycated catalase is responsible for mitochondrial dysfunction and increased oxidative stress in the Parkinson’s disease brain, promoting disease progression (Mofidi Najjar et al. 2017). In addition, glycated catalase may interact with other proteins, such as α-synuclein, thereby affecting the formation of protein aggregates and Lewy bodies.

### Glutathione peroxidase

Glutathione peroxidase plays a key role in protecting cells from oxidative damage by reducing hydrogen peroxide with simultaneous oxidation of reduced glutathione (GSH) to its oxidized form (GSSG) (Bjørklund et al. 2021). The glutathione peroxidase-1 (GPX-1) isoenzyme is particularly important in Parkinson’s disease. One of the first studies of impaired GPx activity in 12 idiopathic patients with Parkinson’s disease using a spectrophotometric technique was reported in the mid-1980s (Kish et al. 1985). Since then, various studies have reported decreased GPx activity in the substantia nigra of patients with PD. The altered activity of GPx was accompanied by a decreased level of reduced glutathione (GSH). These findings may confirm the involvement of the GPX-glutathione system in neuronal loss.

The level of GPx was evaluated in Parkinson’s disease patients with PARK2 mutations (Vinish et al. 2011). A slight decrease in GPx activity relative to that of the controls was observed. The brain is rich in unsaturated fatty acids that are prone to lipid peroxidation, whose levels have been documented to be increased in the postmortem brains of Parkinson’s patients.

A study using immunochemistry methods has shown that GPx-1 is highly abundant in microglia and less abundant in neurons in the Parkinson’s disease brain (Power and Blumbergs 2009). Astrocytes contained only negligible amounts of GPx-1, and oligodendrocytes did not, as documented by immunofluorescence experiments. While unshaped Lewy bodies were circled with a layer of GPx-1 that coexisted with α-synuclein, aligned Lewy bodies exhibited discrete deposits of GPx-1 involved in their degradation. The results confirmed that the abnormal α-synuclein present in Lewy bodies produces hydrogen peroxide, and these neurons possess the ability to drive antioxidant enzymes such as GPx-1 to areas experiencing oxidative stress. Furthermore, these findings suggest that GPx-1-positive microglia play a role in neuroprotection in Parkinson’s disease, highlighting GPx-1 as an important antioxidant enzyme involved in neuronal protection.

Taken together, the literature data confirmed considerable variability in GPx levels reported among patients with Parkinson’s disease (Gökçe Çokal et al. 2017). Generally, GPx levels decrease, remain constant, or increase in serum. The increased GPx activity in Parkinson’s disease has been interpreted as a compensatory mechanism that protects against oxidative stress.

### Vitamin C

Vitamin C is an important low-molecular-weight antioxidant in the nervous system that helps reduce oxidative stress, decrease lipid peroxidation, and neutralize the damaging effects of ROS (Zhao et al. 2019a, b; Oudemans-van Straaten et al. 2014). Other nonoxidative stress processes involve the synthesis of collagen, cholesterol, carnitine, amino acids, and certain peptide hormones (Grosso et al. 2013). Reactions involving dopamine produce transient ROS, which may influence the abnormal protein deposits observed in Parkinson’s disease (Belluzzi et al. 2012).

Vitamin C is transported into cells, especially in the brain, by sodium-dependent vitamin C transporter 2 (SVCT2), and its oxidized form, dehydroascorbate, is transported by glucose transporter 1 (GLUT1) and glucose transporter 3 (GLUT3). Vitamin C can restore vitamin E from its oxidized form (tocopheroxyl radical Toc-O^•^) to its reduced form (Toc-OH) at the water‒lipid interface (see also below). At high concentrations, vitamin C, which acts as a prooxidant, can activate nuclear factor erythroid 2-related factor 2 (NRF2); however, the mechanism is complex. High doses may produce hydrogen peroxide, which can trigger the activation of the NRF2 pathway (Wagner et al. 2011). Some studies suggest that vitamin C promotes NRF2 translocation into the nucleus and activates downstream antioxidant genes (Smith et al. 2016). The synergistic effect with other antioxidants, such as flavonoids, enhances NRF2 activation and antioxidant gene expression on the basis of cumulative effects (Liu et al. 2022).

Vitamin C can also counteract glial cell activation, including that of microglia and astrocytes, and suppresses TLR-induced NF-kappaB activation, regulating NLRP3 expression in the TLR/NF-κB/NLRP3 pathway, which decreases the production of proinflammatory cytokines such as tumor necrosis factor-α (TNF-α) and interleukin-1β (IL-1β) (Liu et al. 2017).

In a large Swedish trial assessing dietary antioxidants and Parkinson’s disease risk, 43,865 men and women aged 18–94 years were followed from 1997 to 2016 (Hantikainen et al. 2021). Baseline dietary intake of vitamin C, vitamin E, and beta-carotene, as well as total nonenzymatic antioxidant capacity, was assessed using a validated food frequency questionnaire. The results confirmed an inverse relationship between dietary vitamin C and E intake and Parkinson’s disease risk. Beta-carotene showed no positive or negative correlation. This study provides Class III evidence supporting the beneficial effects of vitamins C and E in relation to the risk of Parkinson’s disease.

### Vitamin E

In a study using Swiss albino mice, haloperidol was injected to induce Parkinson’s disease (Iqbal et al. 2022). This study examined the beneficial effects of δ-tocopherol, a form of vitamin E and a tocopherol derivative, against haloperidol-induced Parkinson’s disease. δ-tocopherol was given orally at various doses ranging from 5 to 40 mg/kg. It increased dopamine levels, which improved symptoms such as catalepsy and locomotion. The 20 mg/kg dose provided the greatest increase in movement activity. Additionally, the expression of markers of oxidative stress decreased, whereas that of neurotransmitters improved, which was associated with decreased levels of NF-κB and α-synuclein. Treatment with δ-tocopherol also reduced inflammatory cytokine and α-synuclein mRNA expression in the animals. Overall, the vitamin E derivative showed therapeutic benefits against haloperidol-induced Parkinson’s disease.

Another study explored Trolox, a water-soluble antioxidant and vitamin E analog, in a mouse model of Parkinson’s disease induced by 1-methyl-4-phenyl-1,2,3,6-tetrahydropyridine (MPTP) (Atiq et al. 2023). Multiple biochemical tests have shown that MPTP increases the expression of α-synuclein and dopamine transporter proteins in the striatum and substantia nigra pars compacta, thereby worsening motor function. Trolox increased the expression of Nrf2 and HO-1 (heme oxygenase-1), thereby reducing oxidative stress. It also decreased neuroinflammation, as indicated by lower levels of phosphorylated NF-κB and the inflammatory cytokine TNF-α in the brains of Parkinson’s disease model mice. Together, these findings suggest that Trolox may protect dopamine neurons from MPTP-induced oxidative stress, inflammation, motor problems, and neurodegeneration.

A large cohort study in Sweden evaluated the role of antioxidants, including vitamin E, in 44,000 people aged 18–94 years from 1997 to 2016 (see also preceding paragraph) (Hantikainen et al. 2021). After adjusting for energy intake, 465 patients with Parkinson’s disease were identified after 17.6 years. The intake of vitamin E (and vitamin C) is inversely related to the risk of developing Parkinson’s disease.

A case‒control study with 100 Parkinson’s disease patients and 100 healthy controls also revealed that sufficient vitamin E intake was linked to a lower incidence of the disease, regardless of age or sex, although it did not affect disease severity (Schirinzi et al. 2019). In support of these results, the protective role of vitamin E was also tested in a *PINK1*^−/−^ mouse model of Parkinson’s disease. Long-term vitamin E and Trolox treatment fully restored synaptic plasticity in the corticostriatal pathway in these mice. Vitamin E helps counteract PINK1 gene deficiency and reverse mitochondrial dysfunction. Overall, both the clinical and experimental data suggest that vitamin E could reverse Parkinson’s disease symptoms.

### Flavonoids

Flavonoids can increase the antioxidant capacity of neuronal cells, helping to prevent neurodegeneration (Magalingam et al. 2015). For example, quercetin glycosides, rutin, and isoquercitrin can stimulate the production of antioxidant enzymes such as SOD, GPx, CAT, and glutathione in PC-12 cells, which are derived from a rat adrenal medulla tumor and respond to the neurotoxin 6-hydroxydopamine (6-OHDA), which is used in animal models to mimic Parkinson’s symptoms (Açar et al. 2023; Magalingam et al. 2015; Yang et al. 2025). Moreover, flavonoids such as quercetin, fisetin, and methylgallate have been shown to protect neuronal cells from oxidative stress by increasing intracellular glutathione (GSH) levels (Pietta 2000a, b).

Luteolin, a common flavonoid abundant in vegetables and fruits, dose-dependently reduces the decrease in [(3)H]dopamine uptake and the loss of tyrosine hydroxylase-immunoreactive neurons (TH-IR) caused by lipopolysaccharide (LPS) in primary mesencephalic neuron–glia cultures (Chen et al. 2008a, b). Luteolin also suppressed microglial activation and the production of proinflammatory cytokines such as tumor necrosis factor-alpha (TNF-α), RNS such as nitric oxide (NO^•^), and ROS such as superoxide in both mesencephalic neuron–glia- and microglia-enriched cultures. These findings suggest that the flavonoid luteolin may help protect dopaminergic neurons against LPS-induced damage.

Curcumin, a yellow compound produced by Curcuma longa plants, has neuroprotective effects on the neurotoxin 1-methyl-4-phenyl-1,2,3,6-tetrahydropyridine (MPTP), which damages dopaminergic neurons in a manner similar to Parkinson’s disease (Harish et al. 2010). Curcumin shields mitochondria from increased ROS levels and depletes reduced glutathione both in vitro and in vivo (Mythri et al. 2007). It also contains structural groups such as hydroxyl and methoxy groups that bind redox-active metals such as copper and iron, offering protection against oxidative damage. Furthermore, curcumin can activate the enzyme glutamate cysteine ligase (GCL), promoting in vivo synthesis of GSH through increased transcription of GCL genes (Dickinson et al. 2003).

Baicalein, a flavonoid from the herb Scutellaria baicalensis Georgi, shows anti-PD activity by reducing motor symptoms (Zhao et al. 2021). After two weeks of rotenone administration, a pesticide used to model Parkinson’s disease, mice exhibiting depression-like behaviors were divided into three groups and given either saline, baicalein, or the drug Madopar orally for four weeks. Behavioral, neuroinflammatory, neurotransmitter, and synaptic plasticity assessments revealed that four weeks of baicalein treatment reduced depression-like behaviors, maintained neurotransmission, decreased alpha-synuclein aggregation, and decreased neuroinflammation. Additionally, brain-derived neurotrophic factor (BDNF) binds to the TrkB receptor on neurons, triggering an intracellular signaling cascade that activates protein kinase A (PKA). PKA then phosphorylates CREB (p-CREB), which promotes gene transcription. This BDNF/TrkB/CREB pathway supports neuronal survival, neuroplasticity, and cognition. Therefore, baicalein might be useful in alleviating Parkinson’s disease-related symptoms.

In addition to treating hypertension, reserpine is also used to induce Parkinson’s disease (Goel and Chaudhary 2020). Reserpine causes tremor, stiffness, oxidative damage, and akinesia. In an experiment, adult Wistar rats received reserpine for five days (5 mg/kg/day, intraperitoneally). Brain tissues were analyzed for glutathione levels, lipid peroxidation, and behavioral changes. Daidzein, an isoflavone and natural phytoestrogen, was then given orally at doses of 50 and 100 mg/kg/day for five days. Compared with the control, daidzein significantly reversed reserpine-induced symptoms and improved brain tissue health. These results suggest that daidzein warrants further investigation as a potential adjunct therapy for Parkinson’s disease.

The powerful flavonoid quercetin has shown neuroprotective properties in vivo in a 6-hydroxydopamine (6-OHDA) rat model and in vitro in 6-OHDA-treated PC12 cells (Wang et al. 2021). In vitro, quercetin (20 µM) enhanced mitochondrial quality control, reduced oxidative stress, increased the protein levels of the markers PINK1 and Parkin, and decreased the protein level of alpha-synuclein in 6-OHDA-treated PC12 cells. In an animal study, rats given quercetin (10 mg/kg/day and 30 mg/kg/day) orally for 14 days presented reduced motor impairments, less neuronal death, decreased mitochondrial damage, and lower alpha-synuclein accumulation. The neuroprotective effects of quercetin decreased when either Pink1 or Parkin was silenced.

### Carotenoids

Carotenoids include more than 700 different structures and are among the most common and abundant lipid-soluble pigments in nature, especially those found in vegetables, fruits, and green leafy vegetables. They consist of a polyene 40-carbon chain skeleton derived from eight isoprene units (Krinsky 1998). A key structural feature of carotenoids, which are responsible for absorbing visible light and their color, includes a chain of nine conjugated double bonds.

Owing to their antioxidant and anti-inflammatory properties, carotenoids may help reduce the symptoms of Parkinson’s disease, although research findings are inconsistent (Jiao 2023). Some studies suggest that the frequent consumption of carotenoids could be linked to a slower progression of Parkinson’s symptoms. However, other studies have shown no correlation or even contradictory results regarding the role of carotenoids in the risk and progression of Parkinson’s disease.

Astaxanthin is a red, fat-soluble carotenoid pigment known for its potent antioxidant, anti-inflammatory, and antiapoptotic properties in Parkinson’s disease. Its effects on 6-hydroxydopamine (6-OHDA)-induced apoptosis in the human neuroblastoma cell line SH-SY5Y have been studied (Ikeda et al. 2008). Astaxanthin reduced apoptosis in SH-SY5Y cells in a dose-dependent manner and prevented typical signs of mitochondrial dysfunction, including a decrease in membrane potential and the cleavage of caspase 9, caspase 3, and poly(ADP‒ribose) polymerase. Western blot analyses revealed that astaxanthin effectively blocked the phosphorylation of p38 MAPK, which is responsible for mitochondrial issues and cell death, but did not affect c-jun NH(2)-terminal kinase 1/2 or extracellular signal-regulated kinase 1/2.

Treatment of human SH-SY5Y dopaminergic neuroblastoma cells with neurotoxins such as oxidized omega-3 fatty acid (DHA) derivatives such as DHA-hydroperoxide (DHA-OOH) and 6-hydroxydopamine (6-OHDA) significantly decreased cell viability, mainly through increased ROS. Pretreating the cells with 100 nM astaxanthin protected them against DHA-OOH and 6-OHDA, reducing mitochondrial dysfunction, apoptosis, and ROS production (Liu et al. 2009). Therefore, astaxanthin appears to be a promising carotenoid for combating neurotoxin-induced neurodegeneration related to oxidative stress.

Inflammation also plays a role in Parkinson’s disease development, as activated microglia produce peroxynitrite, which is toxic to dopaminergic neurons. Astaxanthin has been shown to protect these neurons in the substantia nigra pars compacta from damage caused by mitochondrial respiratory chain dysfunction, peroxynitrite toxicity, oxidative stress, and inflammation (McCarty et al. 2020).

Clinical trials with carotenoids have yielded mixed results. Some studies suggest that high intake of β-carotene is linked to a lower risk of developing Parkinson’s disease (Wu et al. 2022; Su et al. 2024). Other studies have indicated that increased intake of total carotenoids, lutein-zeaxanthin, and β-carotene may slow the progression of motor symptoms in Parkinson’s disease patients (Hughes et al. 2016).

A systematic review and meta-analysis of observational studies from 1966–March 2005, covering major databases, did not find any protective effects from β-carotene intake (Etminan et al. 2005).

The neuroprotective effects of lycopene in a mouse model of Parkinson’s disease induced by 1-methyl-4-phenyl-1,2,3,6-tetrahydropyridine (MPTP) have been studied (Prema et al. 2015). Orally administered lycopene at doses of 5, 10, and 20 mg/kg/day helped counteract the MPTP-triggered depletion of striatal dopamine and its metabolites in a dose-dependent way, also reducing Parkinson’s motor symptoms. Its antiapoptotic and antioxidant properties, confirmed by Western blot analysis, help reverse neurochemical deficits, oxidative stress, and cell death in Parkinson’s disease model mice. Lycopene appears to restore the neurochemical balance, reduce oxidative stress, and prevent cell death caused by the MPTP.

Rats given rotenone (3 mg/kg) intraperitoneally for 30 days showed significant 35% inhibition of NADH dehydrogenase in the striatum, which indicates that rotenone has an effect (Kaur et al. 2011). Compared with rotenone alone, oral administration of lycopene (10 mg/kg) for 30 days increased NADPH activity by 39%. Rotenone increased malondialdehyde (MDA) levels by 75% in the striatum, but lycopene reduced these levels by 24%. Additionally, rotenone reduced glutathione levels by 43%, but lycopene increased them by 75%. The activity of superoxide dismutase decreased by 69% with rotenone, yet lycopene restored it by 12% relative to that of the control. Furthermore, lycopene prevented the release of cytochrome c from the mitochondria. Overall, these results suggest that lycopene helps reduce the damage caused by rotenone in Parkinson’s disease models.

## Huntington disease

Huntington’s disease (HD) was first reported in 1872 (Huntington 1872). It is a rare neurodegenerative neuropsychiatric inherited genetic disorder caused by a mutation in the HTT gene on chromosome 4, characterized by an abnormal expansion of a CAG (cytosine, adenine, guanine) triplet repeat sequence (Jiménez-Jiménez et al. 2025). As the size of the CAG segment increases, an abnormally long huntingtin protein is produced. A descendant needs only one copy of the altered gene from one parent to have a 50% probability of inheriting this disease.

A combination of movement disorders, cognitive decline, and psychiatric disturbances characterize individuals affected by Huntington’s disease. The hallmark of involuntary movements affects the whole body, particularly the face. In addition, people suffering from HD have difficulties controlling voluntary movements and other motor issues, such as rigidity, problems with posture and balance, and difficulties with swallowing and speech (McColgan and Tabrizi 2018).

The production of abnormal huntingtin associated with neurodegeneration, a key histopathological hallmark of Huntington’s disease, predominantly affects part of the basal ganglia—the striatum—and the cerebral cortex—particularly the frontal lobe. While the pathogenesis of the mechanism by which Huntington’s disease develops is largely unknown, factors such as GABA dysfunction, neuroinflammation, oxidative stress, excitotoxicity, a dysregulated immune response, mitochondrial dysfunction, abnormal fragmentation of Huntingtin, the formation of toxic clumps, and a decrease in neurotrophic factors, particularly brain-derived neurotrophic factor (BDNF), are associated with this disease.

### Sources of oxidative stress in HD

Various studies have provided evidence that oxidative stress is a key hallmark in the pathophysiology of Huntington’s disease (Kumar et al. 2016). Although many biomarkers of oxidative stress described above have pathological value, it remains unclear whether oxidative stress is a primary event or a consequence of other primary events.

### Mitochondria

ROS-induced oxidative stress in Huntington’s disease results directly from altered redox signaling (Paul and Snyder 2019). A key factor contributing to redox imbalance is mitochondrial dysfunction. When dysregulated, mitochondria, the sites of oxidative phosphorylation (OXPHOS), lead to ROS-induced oxidative stress through multiple pathways. Studies report decreased activity in complexes II, III, and IV, causing a reduction in the electrical potential difference across the inner mitochondrial membrane, which impairs mitochondrial bioenergetics and ATP production via oxidative phosphorylation (Gu et al. 1996). The proximity of the electron transport chain to mitochondrial DNA makes the genome especially vulnerable to oxidative damage, which has been evidenced by increased levels of free radical adducts such as 8-OH-dG in DNA. Oxidative damage in Huntington’s disease primarily occurs in the parietal region, which is responsible for dementia, and progresses into the later stages of the disease (Polidori et al. 1999). Mutant huntingtin protein (mHtt) can disrupt mitochondrial function, leading to increased oxidative stress. For example, mHtt interacts with and impairs the expression of several transcription factors essential for mitochondrial health, such as peroxisome proliferator-activated receptor gamma coactivator 1-α (PGC 1-α), a major regulator of mitochondrial biogenesis and energy metabolism (St-Pierre et al. 2006). Additionally, mHtt affects the activity of cAMP response element-binding protein (CREB), a transcription factor activated by signaling pathways involving cyclic AMP (cAMP) and other proteins, thereby decreasing PGC-1α expression.

### Dyshomeostasis of transition metals in HD

Dyshomeostasis of both redox and nonredox metals is an important hallmark that interferes with other hallmarks of the majority of neurodegenerative diseases (Paul and Snyder 2019). An increased pool of iron accumulated in both neurons and glial cells has been detected in the basal ganglia of individuals with Huntington’s disease (Bartzokis et al. 2007; Bartzokis and Tishler 2000). Deferoxamine, an effective iron chelator, has been shown to provide a neuroprotective effect (Simmons et al. 2007; Chen et al. 2007). Iron is typically associated with copper, and its accumulation in Huntington’s disease, which contributes to neurodegeneration, is no exception (Dexter et al. 1992). Copper ions are characterized by the high plasticity of their coordination polyhedron. This feature is manifested by the coordination of copper to the nitrogen-based terminal region of the mutant mHtt, supporting its aggregation and hindering its clearance (Fox et al. 2011). As discussed above, partially owing to their low redox potentials, redox-active metal ions can readily change their oxidation states, thereby transferring electrons to form a variety of ROS, such as the superoxide radical anion. The catalytic action of iron and copper ions is documented by the Fenton reaction, which forms hydroxyl radicals and causes significant damage to neuronal cells.

### Altered levels of low-molecular-weight antioxidants in HD

### Glutathione

Glutathione (GSH) is a tripeptide (glutamate, cysteine, glycine) and one of the most efficient cellular antioxidants. Fourteen patients with Huntington’s disease and 39 controls participated in the evaluation of oxidative damage in blood by measuring biomarkers of oxidative damage, and among several antioxidants, they also reduced glutathione (Peña-Sánchez et al. 2015). The results confirmed a significant redox imbalance in patients with HD and reduced levels of glutathione associated with caudate atrophy, a hallmark of the neuroimaging characteristics of HD characterized by the shrinking of the caudate nucleus, a part of the brain’s basal ganglia.

In contrast to other postmortem cortex evaluations of decreased GSH in HD patients, other studies reported increased intracellular levels of both reduced (GSH) and oxidized (GSSG) glutathione in striatal cells derived from HD knock-in mice expressing mutant Htt versus wild-type cells (Ribeiro et al. 2012). A minor increase in total intracellular glutathione may be accounted for by a reduction in extracellular glutathione within mutant cells.

### Vitamin C

Vitamin C is a water-soluble, powerful antioxidant that is transported into and concentrated in the brain and neurons via sodium-dependent vitamin C transporter-2 (SVCT2), where it acts as a messenger affecting neuronal metabolism (Acuña et al. 2013). Considering the importance of vitamin C in the synthesis of dopamine and neurotransmission, vitamin C transport deficiency in the progression of Huntington’s disease pathology and associated behavioral deficits have been studied in human huntingtin protein-expressing YAC128 mice (Tienda et al. 2025). These findings revealed that although a moderate deficiency in vitamin C did not exacerbate early behavioral phenotypes in the YAC128 model, it did influence the regulation of the dopamine system at the molecular level. Thus, vitamin C may interfere with disease progression.

Synaptic activity is accompanied by the release of vitamin C into the extracellular space, where it is subsequently absorbed by neuronal cells by the sodium-dependent vitamin C transporter SVCT2 (Acuña et al. 2013). Intracellular ascorbic acid maintains redox balance and sustains the energy supply, which promotes the uptake of monocarboxylates such as pyruvate and lactate. A study employing the R6/2 mouse model of human Huntington’s disease (HD) has shown that mutant huntingtin mHtt impairs SVCT2 translocation. The results demonstrated that the homeostasis of vitamin C is altered even before the onset of overt HD-like behavioral symptoms in an HD mouse model. Given that neuronal intracellular vitamin C stimulates the uptake of lactate to optimize energy production, its impaired uptake by HD neurons could lead to metabolism-mediated neuronal death.

### Antioxidant enzymes and HD

The most efficient antioxidant shield in HD is provided by antioxidant enzymes such as superoxide dismutases (SOD1–3), catalase, glutathione peroxidases (GPx), peroxiredoxins (PRDXs), and other antioxidant enzymes (Jomova et al. 2024). Several studies have confirmed decreased activities of antioxidant enzymes in individuals with HD. Cu, Zn-SOD (SOD1) is an efficient scavenger of superoxide radicals, and glutathione peroxidase (GPx) effectively scavenges lipid hydroperoxides. The impact of pathological alterations in a group of 16 Huntington’s disease patients on the levels of SOD1 and GPx in peripheral tissues was evaluated (Chen et al. 2007). The levels of both enzymes in erythrocytes were reduced, and the levels of oxidative stress markers, including plasma malondialdehyde (MDA) and leukocyte 8-hydroxy-2’-deoxyguanosine (8-OHdG), were increased.

Mutated Huntingtin (mHtt) not only affects the activity of antioxidant enzymes but also interferes with transcription factors such as specificity protein 1 (SP1) in SP1- (activating transcription factor 4) (O’Connor et al. 2016). The pathophysiology of Huntington’s disease is characterized, among other characteristics, by the depletion of cystathionine γ-lyase (CSE), an enzyme that catalyzes the final step in cysteine biosynthesis. Supplementation with cysteine reversed alterations in tissues and animal models of Huntington’s disease (Paul et al. 2014a, b).

Nuclear factor erythroid 2-related factor (Nrf2) is a key antioxidant transcription factor involved in many beneficial processes and has been found to be dysregulated in many neurodegenerative disorders, including Huntington’s disease, which is not an exception (Dinkova-Kostova et al. 2018). The HECT domain and ankyrin repeat-containing E3 ubiquitin protein ligase 1 are encoded by HACE1 gene and promote the activity of Nrf2. This HACE1‒Nrf2 axis plays an important role in cellular responses to neuronal damage in Huntington’s disease as well as in cancer (Rotblat et al. 2014). The striatum of patients suffering from Huntington’s disease shows loss of HACE1. Restored HACE1 protects against oxidative stress and underscores the importance of Nrf2 as an emerging player in neurodegeneration.

### Calcium homeostasis in HD

Calcium (Ca^2+^) ions are essential for a variety of biological processes, such as bone formation, muscle contraction, and cell signaling, and serve as crucial universal second messengers that modulate a variety of cellular functions (Kolobkova et al. 2017). The concentrations of calcium ions oscillate between 100 and 300 nM and are tightly controlled by an array of channels, pumps, exchangers, and protein buffers.

Fragments of mHtt interfere with the expression of certain genes responsible for calcium homeostasis. Therefore, dysregulated calcium signaling in Huntington’s disease originates at the transcriptional level, where notable variations in the mRNA levels of genes encoding proteins that regulate intracellular calcium occur (Zuccato et al. 2010; Luthi-Carter et al. 2002).

Store-operated calcium entry (SOCE) refers to a mechanism for calcium influx involving the calcium sensors STIM1 and STIM2, along with the channel proteins Orai1, Orai2, and Orai3 (Czeredys 2020). The SOCE mechanism is activated when the calcium levels in the endoplasmic reticulum are decreased. Abnormal activation of SOCE results in dysregulated Ca^2+^ homeostasis, which is believed to contribute to the progression of Huntington’s disease (HD). Abnormal activation of SOCE is associated with the occurrence of mutant Huntingtin (mHTT) in the striatum of HD models, preceding the first symptoms of the disease.

MHtt may interact with many cellular targets, including mitochondrial membranes and Ca-binding proteins, and regulate the influx and efflux of calcium ions, which ultimately affects calcium signaling (Kolobkova et al. 2017). The interaction of mHtt with calcium-binding proteins increases intracellular calcium levels and disables proteins (Kreutz et al. 2012). Calmodulin is an intermediary protein that senses calcium levels and transmits signals to ion channels and calcium-sensitive enzymes, and its interaction with mHtt is an integral part of a large protein complex (Bao et al. 1996).

Calpain is a proteolytic enzyme belonging to the family of calcium dependent, nonlysosomal cysteine proteases. A model study of Huntington’s disease revealed that the application of calpastatin, an endogenous protein inhibitor of calpain, prevented the aggregation and toxicity of mHtt (Menzies et al. 2015). The results of this study can provide solid ground for the development of new approaches for HD therapy.

There are two types of glutamate receptors, ionotropic and metabotropic, and their activity is increased in Huntington’s disease. This is manifested by the increased transport of calcium into the cytosol via the excitatory N-methyl-D-aspartate (NMDA) receptor, which is correlated with the increased effect of mutant huntingtin (mHtt) (Fan et al. 2007 a). Variations in NMDAR activity have been associated not only with disrupted calcium homeostasis but also with mitochondrial membrane depolarization and the activation of caspases (Fan et al. 2007 b). Increased expression of NMDARs in different cell types is correlated with degeneration of the main type of neurons in the striatum, i.e., medium spiny neurons.

### Cysteine in HD

Cystathionine γ-lyase (CSE), cystathionine β-synthase (CBS), and 3-mercaptopyruvate sulfurtransferase (3-MST) are three important enzymes that use cysteine or its derivatives to generate hydrogen sulfide (H_2_S), a gasotransmitter produced in mammalian tissues that acts as a key signaling molecule in many biological processes, including neurological and cardiovascular functions (Kolluru et al. 2013). Dysregulated cysteine metabolism and altered CSE expression negatively affect the production of hydrogen sulfide. The activity of hydrogen sulfide in the brain includes enhancing certain receptors and playing an important role in the formation of memory (Sbodio et al. 2019).

Transcriptional activator specificity protein 1 (Sp1) is a critical activator of human cystathionine gamma-lyase expression. It has been proposed that inhibition of Sp1 by mHtt accounts for CSE depletion in Huntington’s disease and associated neurological deficits (Paul et al. 2014a, b). This finding was supported by the therapeutic effects of cysteine and *N*-acetylcysteine in mice with Huntington’s disease, showing that *N*-acetylcysteine supplementation may be beneficial in treating neurodegenerative diseases.

ATF4 is a transcription factor that regulates gene expression by binding to DNA. Under oxidative stress conditions, the expression of CSE is regulated by stress-responsive activating transcription factor 4 (ATF4); however, in Huntington’s disease cells, the activation of ATF4 is dysregulated, resulting in suppressed CSE expression and impaired cysteine biosynthesis (Sbodio et al. 2016).

Cystine is an oxidized form of cysteine, where two cysteine molecules are connected by a disulfide (-S-S-) bond to form cystine (Bonifácio et al. 2021). This oxidation can occur spontaneously at neutral pH or be accelerated by ionizing radiation, oxygen, or the presence of metal ions such as copper and iron. In the cystine/glutamate antiporter process, the transporter xCT facilitates the uptake of cystine, the oxidized form of cysteine, into cells, where it is converted to its monomeric, reduced cysteine form under reducing conditions (Li et al. 2010). In Huntington’s disease, the activity of xCT is reduced, resulting in deficient cystine metabolism.

### Conventional medical treatment of Huntington’s disease

### Movement disorders

There is no available treatment to reverse the progression of HD. Medications can alleviate certain symptoms related to movement and mental health issues (Frank 2014). The approved medications that regulate movement include tetrabenazine (Xenazine), deutetrabenazine (Austedo), and valbenazine (Ingrezza); however, they have no effect on disease progression. Potential side effects may include restlessness and a risk of triggering depression or other psychiatric disorders. Antipsychotic medications, such as haloperidol and fluphenazine, along with olanzapine (Zyprexa) and aripiprazole (Abilify, Aristada), are side effects of movement suppression. Side effects include muscle contractions and slowness of movement. They may also lead to restlessness and drowsiness.

### Mental health

Medications aimed at addressing mental health issues are antidepressants such as citalopram (Celexa), escitalopram (Lexapro), fluoxetine (Prozac), and sertraline (Zoloft). Possible side effects include nausea, diarrhea, drowsiness, and hypotension. Antipsychotic medications such as quetiapine (Seroquel) and olanzapine (Zyprexa) can mitigate violent outbursts, agitation, and other related symptoms. Nevertheless, these medications may induce various movement disorders as side effects. Mood-stabilizing medications can prevent the extreme emotional fluctuations associated with bipolar disorder. These include divalproex (Depakote), carbamazepine (Tegretol, Carbatrol, Epitol, among others), and lamotrigine (Lamictal). Supporting therapies include psychotherapy, speech therapy, physical therapy, and other therapies.

### Antioxidant therapy in Huntington’s disease

Rodent models of Huntington’s disease are commonly employed to evaluate pharmacological and other therapeutic interventions. Certain models exhibit altered expression levels of nitric oxide synthase (NOS) and superoxide dismutase (SOD), increased ROS levels, and increased lipid peroxidation (Bono-Yagüe et al. 2020). Animal models in which low-molecular-weight antioxidants are used to mitigate oxidative stress caused by mutant huntingtin (mHtt) are valuable preclinical tools that establish a foundation for prospective clinical trials in HD patients.

Supplementation in patients with Huntington’s disease has some positive effects on fighting oxidative stress, but the clinical results have been inconsistent (Johri and Beal 2012a, b). The idea of antioxidant protection against Huntington’s disease focuses on increasing the body’s natural defenses by using naturally occurring antioxidants such as flavonoids and developing specialized mitochondrion-targeted antioxidants (MTAXs) to neutralize ROS at their source. While preclinical studies and mouse models have shown benefits, more research is needed to identify the safest and most effective antioxidant strategies for human HD patients, especially in the early stages.

### Ascorbate, lipoic acid, melatonin

Ascorbic acid (vitamin C), a potent antioxidant present as an ascorbate anion (AscH^−^), can be oxidized by various ROS into ascorbyl radicals and eventually into dehydroascorbic acid (DHA). Ascorbic acid is vital for maintaining healthy neuronal metabolism. It is absorbed in the small intestine through two main mechanisms: active transport by sodium-dependent vitamin C transporters (SVCTs) at low doses and facilitated diffusion via glucose transporters (GLUTs) in the oxidized dehydroascorbic acid form (Castro et al. 2009). The efficiency of absorption depends on the dose, which generally decreases with excessive intake, as excess is eliminated by the kidneys.

The frequently used R6/2 mouse model of Huntington’s disease demonstrates a deficit in extracellular ascorbate in the striatum, which may play a role in the behavioral phenotype associated with Huntington’s disease (Rebec et al. 2003). The regular administration of ascorbate (300 mg/kg/day) restored the behavior-related release of ascorbate in the striatum and improved behavioral responses. Treatment with ascorbate significantly reduced the neurological motor symptoms of HD without impacting overall motor activity.

While the exact function of ascorbate in striatal activity remains ambiguous, a growing body of evidence suggests that it is crucial to observe the HD striatum for potential ascorbate imbalances. Similarly, the extracellular dynamics of ascorbate in the striatum of the R6/2 mouse model of Huntington’s disease have been investigated (Rebec et al. 2002). Striatal ascorbate levels during anesthesia and subsequent recovery were monitored via slow-scan voltammetry. HD mice displayed steady-state ascorbate levels during anesthesia. The gradual re-emergence of behavioral activation resulted in markedly different ascorbate responses: a steady increase in controls contrasted with a 25–50% reduction in HD mice, exhibiting a broad spectrum of motor impairments in agreement with a significant reduction in ascorbate in the striatal extracellular fluid. These findings are in agreement with insufficient antioxidant protection in the HD striatum; they suggest that the ascorbate deficit is restricted to times of behavioral activation.

Lipoic acid (α-lipoic acid) is a naturally occurring, vitamin-like antioxidant produced in the body and found in meats and vegetables. The neuroprotective effects of lipoic acid were tested using popular R6/2 and N171-82Q mouse models of Huntington’s disease (Andreassen et al. 2001). The results revealed significant improvements in survival in both mouse models, suggesting that lipoic acid can be beneficial for people with Huntington’s disease.

Melatonin, a hormone naturally produced by the brain’s pineal gland, regulates the sleep cycle and has antioxidant properties. Its effects on oxidative stress caused by 3-nitropropionic acid were studied in synaptosomes in an animal model of Huntington’s disease (Túnez et al. 2004). The study revealed increased levels of lipid peroxidation, protein carbonyls, and SOD activity caused by 3-nitropropionic acid. These harmful changes were reduced with melatonin pretreatment, likely due to its antioxidant effects.

Additionally, 3-nitropropionic acid (3-NP) causes biochemical disruptions involving nitric oxide pathways and affects the glutathione system. To protect against this, the antioxidant pigments lycopene and epigallocatechin-3-gallate (EGCG) were tested (Kumar and Kumar 2009). The study revealed that 3-nitropropionic acid reduced glutathione, total glutathione, and glutathione-S-transferase levels in the striatum, hippocampus, and cortex of the brain. Supplementing with lycopene (2.5, 5, or 10 mg/kg) and EGCG (10, 20, or 40 mg/kg) significantly improved memory and helped restore the glutathione defense system. Therefore, lycopene and EGCG can help manage the behavioral and biochemical changes caused by 3-NP, mainly through nitric oxide pathways (Essa et al. 2019).

### Polyphenols

Polyphenols are known for their ability to neutralize ROS and chelate redox metals, offering potential benefits against neurodegeneration. Modulation of the NRF2 pathway appears to be a promising target for ameliorating symptoms of HD, particularly through polyphenol-mediated enhancement of NRF2 activity. Animal models of Huntington’s disease have served as promising means for testing the beneficial effects of naturally occurring polyphenols (D’Egidio et al. 2025). Naringin is a glycosylated flavonoid responsible for bitter taste and is known for its powerful antioxidant, anti-inflammatory, and pharmacological properties. 3-Nitropropionic acid (3NP) inhibits mitochondrial complex II and induces striatal damage that resembles the pathology of Huntington’s disease. Cell lines lacking Nrf2 and Nrf2 knockout mice exhibit significantly heightened susceptibility to 3-NP, along with increased transcription regulated by the antioxidant response element (ARE) in astrocytes (Gopinath et al. 2012). The rats were administered 3-NP for 2 weeks to induce neurodegeneration, whereas naringin (80 mg/kg body weight/day) was given throughout the experimental period, specifically 1 h before exposure to 3-NP. Naringin, via the activation of Nrf2, influences the histological alterations induced by 3-NP, increases the ratio of reduced to oxidized glutathione, and promotes the expression of NAD(P)H: quinone oxidoreductase-1, heme oxygenase-1, glutathione S-transferase P1, and gamma-glutamylcysteine ligase mRNAs. In addition, naringin, through Nrf2, suppressed the expression of proinflammatory cytokines such as TNF-α, cyclooxygenase-2, and iNOS. These data suggest that Nrf2 is a crucial inducible factor in safeguarding against neurotoxicity.

MegaNatural grape seed polyphenolic extract, which is water soluble and free of resveratrol, contains 8% monomers, 75% oligomers, and 17% polymers (Wang et al. 2010). It has been shown to significantly increase survival rates in Drosophila models of Huntington’s disease. Its in vivo effectiveness was tested in an aggressive R6/2 mouse model of HD, in which the polyphenol extract slowed motor decline and extended lifespan.

Lycopene and epigallocatechin gallate (EGCG) are powerful neuroprotective antioxidants that often work synergistically. The effects of lycopene and EGCG on memory deficits and the disruption of the glutathione system in rats caused by the toxin 3-NP, which is used in animal models to mimic Huntington’s disease, were investigated (Kumar and Kumar 2009). 3-NP caused significant memory impairment and suppressed the synthesis of reduced glutathione and glutathione-S-transferase in the striatum, hippocampus, and cortex. Lycopene and EGCG improved memory and restored the glutathione system. Cosupplementation of lycopene with L-arginine increased its protective effect via nitric oxide pathways.

Rutin is a natural flavonoid pigment found in many fruits and vegetables, including buckwheat, apples, and citrus, and is known for its potency antioxidant and anti-inflammatory properties. The neuroprotective effect of rutin against the systemic administration of 3-NP in rats, which results in protein oxidation, reduced locomotor activity, and suppressed mitochondrial activity, has been studied (Suganya and Sumathi 2014). Rutin significantly improved behavioral changes and restored mitochondrial activity. Thus, rutin may represent a potentially suitable flavonoid for ameliorating pathologies associated with Huntington’s disease through its antioxidant properties.

Quercetin is a plant flavonol from the flavonoid group of polyphenols that acts as a potent antioxidant, and its beneficial effect on a 3-nitropropionic acid-induced rat model of Huntington’s disease was investigated (Sandhir and Mehrotra 2013). Quercetin has been shown to reduce lipid peroxidation, which in turn improves mitochondrial bioenergetics, prevents mitochondrial swelling, and restores the activities of the antioxidant enzymes SOD and catalase. The beneficial effect of quercetin was also documented by histopathological analysis, which revealed reduced astrogliosis in the striatum. The obtained results document the beneficial role of quercetin in the management of HD.

Hesperidin is a polyphenolic flavonoid found in the citrus family (Khan et al. 2020a, b). It has ROS-scavenging, anti-inflammatory, anticancer, and antihypertensive properties. The potential neuroprotective effects of hesperidin, particularly in conjunction with minocycline (a microglial inhibitor), against symptoms induced by quinolinic acid (QA) in rat models of Huntington’s disease (HD) have been studied (Kumar et al. 2013).

Myricetin is a naturally occurring flavonoid known for its significant antioxidant and anti-inflammatory properties and is typically present in vegetables, fruits, nuts, and tea. It has been documented that myricetin interacts with the abnormally long expansion cytosine-adenine-guanine (CAG) trinucleotide repeats in the HTT gene. This interaction prevents the translation of the mutant huntingtin protein, as well as the sequestration of muscleblind-like-1 splicing (MBNL1) factor (Khan et al. 2018). The mode of interaction between myricetin and RNA has been described as involving base stacking at the AA mismatch. Additionally, myricetin has been shown to diminish the proteotoxicity caused by polyglutamine aggregation. Thus, myricetin has been demonstrated to be a promising therapeutic candidate for Huntington’s disease and other polyQ-related disorders.

Fisetin is a natural flavonoid found in many common fruits and vegetables, as well as in some herbal teas, and is known for its antioxidant, anti-inflammatory, and senolytic properties. The hypothesis that fisetin activation of ERK could offer protection against Huntington’s disease (HD) was investigated (Maher et al. 2011). Three different models, PC12, R6/2, and mutant Httex1 of Huntington’s disease, were used. These findings suggest that fisetin is capable of mitigating the effects of mutant huntingtin across all these disease models. In addition to fisetin, the related polyphenol resveratrol, which similarly activates ERK, has protective effects on HD models.

### Clinical trials

As discussed above, phytochemicals, particularly natural antioxidants such as flavonoids, have been identified as promising agents for mitigating oxidative stress in numerous neurological investigations (Rebas 2025). In the context of neurological disorders such as Huntington’s disease (HD), both tissue and animal models have confirmed the beneficial effects of flavonoids in alleviating oxidative stress (Sandhir and Mehrotra 2013). A few clinical trials on the efficacy of flavonoids and low-molecular-weight antioxidants in relation to HD have yielded inconsistent outcomes and are briefly discussed (Mähler et al. 2013).

A one-year, double-blind, placebo-controlled clinical trial of α-tocopherol employing 73 patients suffering from Huntington’s disease was conducted (Peyser et al. 1995). The trial results revealed a significant beneficial effect of α-tocopherol on neurological symptoms, predominantly in patients in the early stage of the disease.

Coenzyme Q (CoQ), also known as ubiquinone, is a fat-soluble compound found in cell membranes, where it functions as a powerful antioxidant and electron transporter in the production of energy (ATP) within mitochondria. A multicenter, randomized, double-blind, placebo-controlled trial aimed to evaluate the hypothesis that prolonged administration of high-dose coenzyme Q10 in patients with early-stage HD may mitigate the progressive decline in functional ability associated with HD (McGarry et al. 2017). A total of 609 patients with early Huntington’s disease between 2008 and 2012 across the USA, Canada, and Australia were evaluated for changes in the total functional capacity score from baseline to month 60. The study prematurely ended in July 2014 and revealed no significant differences between the treatment groups. Thus, the observed findings do not support the use of CoQ as a suitable substance to delay the decline in patients with early-stage HD.

Idebenone is a synthetic derivative of ubiquinone, commonly referred to as coenzyme Q10, which serves as a crucial antioxidant within cells and is an integral part of the electron transport chain (ETC). A one-year, double-blind trial with 91 individuals who were diagnosed with mild to moderate clinical Huntington’s disease and who were receiving idebenone was conducted (Ranen et al. 1996). Patients were administered 30 mg of idebenone three times a day or a placebo. No adverse effects associated with idebenone were observed. The results of the trial revealed no treatment effect on the primary outcome measures of the Huntington’s Disease Activities of Daily Living Scale (ADL) and the Quantified Neurologic Examination (QNE). Considering that the neuropathology of Huntington’s disease (HD) more closely resembles the NMDA receptor-mediated degeneration of striatal neurons than the degeneration induced by kainate/AMPA receptor agonists, the lack of a therapeutic effect of idebenone in HD aligns with preclinical observations in animal models.

Ethyl eicosapentaenoic acid, also known as icosapent ethyl, is a highly refined prescription omega-3 fatty acid (EPA) that is utilized to markedly reduce extremely elevated triglyceride levels. The aim of this 6-month multicenter randomized, double-blind, placebo-controlled trial was to determine whether ethyl-eicosapentaenoic acid (1 g twice a day) can improve the motor functions of patients with Huntington’s disease at 41 research sites in the USA and Canada (Huntington 2008). At the 6-month mark, the change in the total motor score of 4 points in patients treated with ethyl eicosapentaenoic acid was comparable to that in patients who received a placebo. No significant differences were observed in the cognitive assessments. The results of the treatment with eicosapentaenoic acid for a continuous duration of 12 months were comparable to those received after active treatment for only 6 months. Taken together, these findings suggest that the administration of ethyl-eicosapentaenoic acid does not have any advantages for patients suffering from Huntington’s disease.

## Amyotrophic lateral sclerosis

Amyotrophic lateral sclerosis (ALS), also known as Lou Gehrig’s disease, is a progressive and ultimately fatal neurodegenerative disorder that affects motor neurons in both the brain and the spinal cord (Mead et al. 2023). This condition leads to a gradual deterioration of voluntary muscle control, resulting in symptoms such as weakness, paralysis, muscle atrophy, and ultimately respiratory failure. Disease is characterized by the death of nerve cells, which disrupts the transmission of signals to the muscles, thereby causing difficulties with everyday activities such as walking, speaking, and swallowing, as well as physiological processes such as breathing.

ALS has an incidence rate of approximately 6–9 cases per 100,000 individuals (Longinetti and Fang 2019). A family history of ALS is documented in 5 to 10% of those affected, typically following an autosomal dominant inheritance pattern. Nevertheless, comprehensive genetic testing has revealed a notable genetic nature in a greater number of ALS patients.

In addition to genetic factors, environmental factors may increase the risk of ALS (Hemerková and Vališ 2021). Given that increased levels of lead have been observed in the blood and bones of patients with ALS, industrial and occupational lead exposure represents a serious threat to ALS (Kamel et al. 2002). The release of lead from bones into the bloodstream reduces glutathione levels, which in turn increases oxidative damage. Astroglia usually show a buffering role for lead. Too high a concentration of lead results in morphological and functional alterations in glial cells, resulting in ROS production (Barbeito et al. 2010).

The risk of developing ALS and the factors that influence the severity of the disease (such as age at onset and rate of progression) seem to be genetically dependent only to some extent, as discussed below. Like other neurodegenerative disorders, ALS is a multifactorial disorder characterized by several contributing factors, with oxidative stress being a key pathological element that plays a substantial role in the ongoing degeneration of motor neurons (Cunha-Oliveira et al. 2020). Oxidative stress and related pathologies involve dysregulated mitochondrial function, disturbed protein homeostasis (proteostasis), DNA damage, neuroinflammation, excitotoxicity, environmental factors, and other pathologies.

### Oxidative stress and ALS

Increased ROS formation and associated damage to all important biomolecules have been documented in a number of studies on ALS (Barber and Shaw 2010). An analysis of samples from patients with ALS revealed increased levels of oxidative damage. Most studies have examined damage to the three most important biomolecules, DNA, proteins, and lipids, in postmortem neuronal tissue, plasma, cerebrospinal fluid, and urine. Increased biomarkers of oxidative damage underscore the involvement of oxidative stress in the pathogenesis of ALS; however, because biomarker values, especially in the early stages of the disease, often fall at the threshold of detection and the life expectancy of ALS patients is short, it would be speculated to declare whether oxidative stress is a primary cause or a consequence of the disease.

The most representative biomarkers of oxidative stress in patients with ALS are the levels of 3-nitrotyrosine and modified protein carbonyls, indicating irreversible protein damage (Ferrante et al. 1997; Niebrój-Dobosz et al. 2004). Key biomarkers of lipid peroxidation include malondialdehyde (MDA), isoprostanes (such as 8-iso-PGF2α), 4-hydroxynonenal (4-HNE), and lipid hydroperoxides, reflecting various phases of lipid degradation, with isoprostanes offering greater specificity and reliability than MDA does, despite the continued widespread use of MDA. Erythrocytes from patients with ALS show elevated lipid peroxidation, which is correlated with suppressed activity of antioxidant enzymes such as catalase, glutathione reductase, and glucose-6-phosphate dehydrogenase, accompanied by decreased levels of glutathione (Babu et al. 2008).

The primary biomarker of oxidative DNA damage is the modified nucleoside 8-hydroxy-2′-deoxyguanosine (8-OHdG). In addition, phosphorylated histones, such as γH2AX (phosphorylated histone H2AX), are classic markers of DNA double-strand breaks (DSBs). Several mitochondrial parameters and antioxidant activities have been analyzed to compare sporadic ALS and familial ALS (Walczak et al. 2019).

Fibroblast samples from patients with sporadic ALS presented suppressed expression of all four mitochondrial electron transport chain complexes, reduced mitochondrial membrane potential, and decreased levels of superoxide dismutase 1 and catalase (Walczak et al. 2019). Samples from familial ALS patients were characterized by high respiration with substrates for complexes I and II and a high level of the complex I NDUFB8 subunit.

### SOD1 mutations, calcium homeostasis, and ALS

Analysis of postmortem tissues and biofluids from patients with ALS revealed increased levels of ROS-induced damage. Interestingly, lymphoblasts from familial ALS, characterized by SOD1 mutations, and fibroblasts from G4C2 repeat expansions within the C9ORF72 gene, the most common genetic cause of ALS, exhibit increased ROS levels. Conversely, fibroblasts and lymphoblasts from sporadic ALS patients do not show signs of increased ROS damage (Shibata et al. 2001).

Like other neurodegenerative diseases, DNA and RNA are prone to oxidative damage in ALS. mRNA oxidation is considered a precursor to motor neuron degeneration. mRNAs play a role in the electron transport chain (ETC) and ATP synthesis, both of which are sensitive targets of oxidative damage (Chang et al. 2008). In addition, oxidative stress may cause misfolding and aggregation of Cu, Zn-SOD (SOD1), manifested by impaired mitochondrial function and increased formation of superoxide radical anions, forming a detrimental loop in which “initial” oxidative stress-mediated misfolding of SOD1 causes mitochondrial damage and “secondary” oxidative stress further exacerbates SOD1 misfolding and subsequent mitochondrial damage (Rakhit et al. 2002).

Oxidative stress and calcium homeostasis are closely linked; ROS-induced oxidative stress disrupts delicate calcium homeostasis within the cell by triggering an influx of calcium into the cytoplasm from internal reservoirs (ER/SR) and from the extracellular environment (Ermak and Davies 2002). This results in mitochondrial overload, compromised cellular function, and increased risk of cell death.

Disrupted calcium homeostasis has been documented in both in vitro and in vivo models of mutant SOD1, as well as in the motor nerve terminals of ALS patients (Gupta et al. 2025). The SOD1-G93A mutation is a specific genetic mutation (glycine replaced by alanine at position 93) in the SOD1 enzyme and represents an extensively studied model for amyotrophic lateral sclerosis. The SOD1-G93A transgenic mouse ALS model is characterized by suppressed mitochondrial calcium capacity in the CNS, indicating that impaired calcium homeostasis may be associated with ALS pathogenesis (Damiano et al. 2006).

In contrast to oculomotor neurons originating in the midbrain, motor neurons in ALS patients have disrupted calcium management, as evidenced by decreased uniporter-dependent mitochondrial calcium uptake (Fuchs et al. 2013). The exact mechanism of deficient mitochondrial calcium uptake is not known; however, SOD1-G93A motor neurons exhibit, following the activation of AMPA receptors, a suppressed and restored capacity for calcium levels (Guatteo et al. 2007).

The endoplasmic reticulum (ER) and mitochondria interact at specialized contact points known as mitochondria-associated membranes (MAMs). These sites serve as essential hubs for orchestrating critical cellular functions, including calcium signaling, lipid metabolism, oxidative stress management, and the regulation of apoptosis (Zhang et al. 2023). Dysregulation of endoplasmic reticulum–mitochondrion processes has been associated with various diseases, including Alzheimer’s disease, Parkinson’s disease, and cancer. The link between the ER and mitochondria is well documented, for example, through homo and heterotypic interactions involving mitofusin (Mfn) 1 and 2, as well as several other protein‒protein interactions. ER‒mitochondrion interactions facilitate the transport of calcium between these two organelles, and mutant SOD1 disrupts this interaction and consequently negatively affects the exchange of calcium ions (Stoica et al. 2014). The interruption of contacts can result in elevated cytosolic calcium concentrations and disturbances in calcium-dependent cellular functions, such as axonal transport, ATP production, and protein homeostasis. Dysregulated calcium in ALS is a primary factor in the apoptosis of motor neurons, which are more susceptible to calcium dysregulation than other types of neurons are (Shaw and Eggett 2000). Motor neurons are abundant in excitatory neurotransmitter calcium-permeable AMPA receptors at the postsynaptic terminal, making them highly vulnerable to excitotoxicity through excessive calcium influx during excitatory neurotransmission events.

Disrupted calcium homeostasis results in several other toxic mechanisms associated with ALS, involving axonal transport and loss of protein homeostasis (De Vos and Hafezparast 2017). Calcium homeostasis is tightly associated with the regulation of enzyme-mediated ATP production and the management of oxidative phosphorylation. A key characteristic of ALS is reduced ATP production, which negatively affects the axonal transport of mitochondria.

Mitochondrial calcium overload leads to reduced calcium levels in the endoplasmic reticulum, resulting in protein misfolding and the induction of ER stress (Webster et al. 2017). Both chronic mitochondrial calcium overload and protein misfolding contribute to the induction of apoptosis. In the next paragraph, we discuss the role of proapoptotic signaling in ALS.

### TDP-43 mutations and ALS

Oxidative stress has been shown to promote acetylation and cysteine oxidation, resulting in aggregation of an essential RNA-binding protein, TDP-43 (TAR DNA-binding protein 43), a typical pathological hallmark of several devastating neurodegenerative diseases, including ALS (Cohen et al. 2015). COS-7 (fibroblast-like cell lines derived from monkey kidney tissue) treated with 4-hydroxynonenal, a byproduct of the lipid peroxidation process, led to the phosphorylation and insolubilization of TDP-43 (Kabuta et al. 2015). The overexpression of TDP-43 has been shown to increase ROS formation and induce oxidative damage (Hong et al. 2012), which in turn, paradoxically triggers the recruitment of TDP-43 to stress granules, thus manifesting its protective properties (Meyerowitz et al. 2011).

### Oxidative stress and apoptosis in ALS

Apoptosis is a process that eliminates cells with irreparable damage and is closely associated with mitochondria. Stress signals such as DNA damage or growth factor withdrawal converge upon the mitochondria, resulting in the permeabilization of the mitochondrial outer membrane (MOMP) and the release of cytochrome c (Duval et al. 2018). This release facilitates the binding of cytochrome c to apoptotic protease-activating factor 1 (Apaf-1), leading to the formation of the apoptosome complex, which subsequently activates caspase-9. This activation triggers downstream executioner caspases, such as caspase-3, which are responsible for the dismantling of the cell. The regulation of apoptosis is mediated by the concerted action of proapoptotic and antiapoptotic proteins belonging to the Bcl-2 family, which are categorized into antiapoptotic members (such as Bcl-2, Bcl-xL, and Mcl-1) that increase cell survival and proapoptotic members (including Bax, Bak, and BH3-only proteins) that initiate cell death (Ashkenazi and Salvesen 2014). The equilibrium between these two groups of proteins determines the fate of a cell. Enhanced apoptotic signaling resulting from toxic processes has been documented in various ALS models.

The accumulation of misfolded mutant SOD1-associated ALS within spinal cord mitochondria is believed to lead to mitochondrial dysfunction; however, the mechanism of toxicity has been the subject of studies. Previous studies revealed that Bcl-2 interacts with mutant SOD1 in the mitochondrial spinal cord (Pasinelli et al. 2004). More recent studies have shown that the toxicity of mutant SOD1 exclusively in the presence of Bcl-2 causes morphological alterations and disruption of mitochondrial membrane integrity, resulting in the release of cytochrome C (Pedrini et al. 2010). In mouse and human cells of the spinal cord, binding to mutant SOD1 triggers conformational changes in Bcl-2, resulting in the activation of the BH3 “death ligand” domain and thus the transfer of Bcl-2 into a toxic protein. Targeting Bcl-2 in mutant SOD1-induced mitochondrial toxicity raises the possibility of therapeutic interventions to prevent mitochondrial damage in ALS through the inhibition of the formation of the toxic mutant SOD1-Bcl-2 complex.

Mitochondrial fission (the process of splitting) and fusion (the process of joining) are tightly coordinated dynamic mechanisms that regulate the shape, quantity, and overall health of mitochondria, characterized by their energy demands and maintenance of quality control (Youle and van der Bliek 2012). Mitochondrial fission is responsible for dividing mitochondria, while fusion combines them into interconnected networks. Mitochondrial fission is mediated by a crucial GTPase enzyme, dynamin-related protein 1 (Drp1), and the process of mitochondrial fusion is regulated by the essential GTPase proteins MFN1 (mitofusin 1) and MFN2 (mitofusin 2), which are located on the outer mitochondrial membrane.

The significance of enhanced mitochondrial fission in the etiology of ALS is not straightforward. Smaller mitochondria are usually more susceptible to ROS-induced damage and may fail to rejoin the mitochondrial network (Hoitzing et al. 2015). Consequently, the observed fragmented mitochondrial network may be directly associated with the diminished mitochondrial membrane potential (MMP), reduced ATP levels, and elevated ROS concentrations noted in ALS. It has been suggested that mitochondrial fragmentation is closely linked to ALS. The SOD1-G93A mutation triggers a decrease in mitochondrial length, accumulation of fragmented mitochondria, and a reduction in neurite length. The restoration of the balance between mitochondrial fission and fusion through the expression of dominant-negative dynamin-related protein 1 (DRP1) mitigates the defects in mitochondrial morphology, dynamics, and cell viability induced by the mutant SOD1-G93A (Song et al. 2013).

### Mitochondrial transport impairments in ALS

Proteins associated with ALS may directly affect the machinery responsible for axonal transport. Impaired mitochondrial axonal transport is a well-established early characteristic of ALS that is essential for the health of motor neurons (Jiang et al. 2015; Smith et al. 2019). Defects have been observed in models featuring mutant SOD1, TAR DNA-binding protein 43 (TDP-43), and fused in sarcoma (FUS), resulting in energy deficits, calcium imbalances, and ultimately axon degeneration, although the precise cause‒and‒effect relationships are complex.

Both TDP-43 and FUS may play a role in regulating the expression of various kinesins, including kinesin-1, whereas TDP-43 is known to bind to TRAK1 mRNA (Xiao et al. 2011; Colombrita et al. 2012). In models of SOD1-G93A and G37R, the ALS-mutant SOD1 has been shown to directly interact with cytoplasmic dynein, which is a motor protein complex that transports cargo (e.g., RNA, viruses) along microtubules toward their negative ends (the center of the cell), and this interaction becomes increasingly significant as the disease progresses (Shi et al. 2010).

**Fig. 6:**
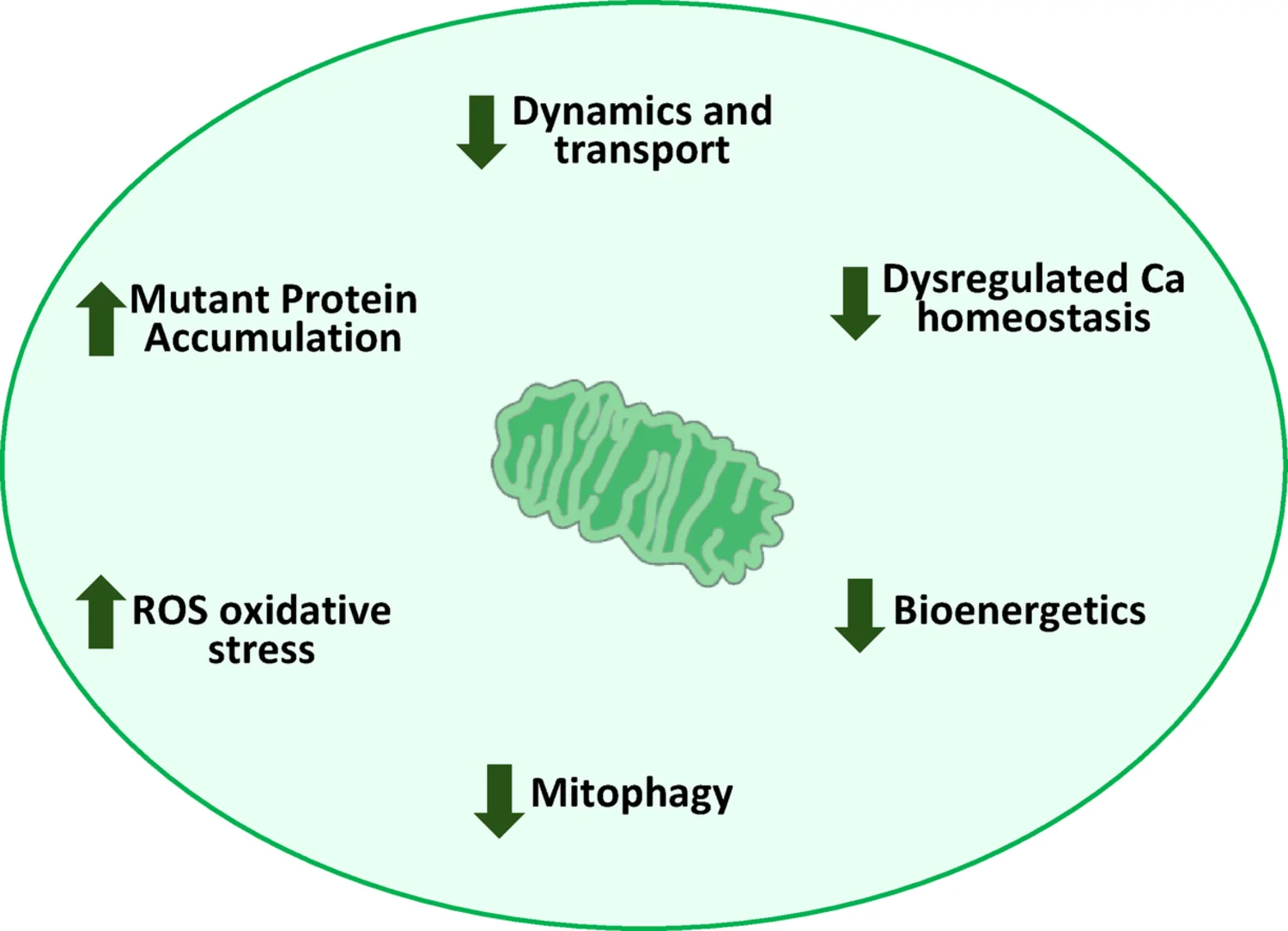
Mitochondrial dysfunction in ALS involves (i) bioenergetic failure related to decreased ATP production, (ii) mitophagy characterized by failed mechanisms responsible for clearing dead or damaged mitochondria, (iii) ROS-induced oxidative stress causing severe damage, (iv) the misfolding and physical accumulation of ALS-related proteins (e.g., SOD1) inside mitochondria, (v) disruption of mitochondrial fusion and fission, which affects their movement along axons, and (vi) dysregulation of calcium homeostasis, which triggers excitotoxicity and apoptosis

Several mechanisms exist through which impaired axonal transport of mitochondria can contribute to disease. These processes involve disrupted ATP production and calcium regulation at synaptic terminals and, together with mitochondrial injury, can lead to axonal degeneration, a typical pathology associated with ALS (Moloney et al. 2014). Disturbed reverse transport is linked to the inability to eliminate damaged organelles via mitophagy, potentially accounting for the mitochondrial clusters observed in the axons of ALS patients (Sasaki et al. 1996). Additionally, impaired mitochondrial axonal transport may affect the movement of other axonal components, such as signaling endosomes, which appear to be closely associated with ALS pathology (De Vos and Hafezparast 2017) (Fig. 6).

### Treatment of amyotrophic lateral sclerosis

While ALS treatment procedures cannot reverse the harm caused by ALS, they are capable of decelerating the progression of symptoms.

Riluzole is a medication that appears to reduce glutamate release from neurons, thereby safeguarding motor neurons from excitotoxicity and subsequent degeneration (Hinchcliffe and Smith 2017). Additionally, Riluzole may influence motor neuron disease (MND) through alternative mechanisms, although this has not been conclusively verified. Riluzole is recommended to be started as soon as possible after diagnosis and may prolong survival by up to 18 months. Riluzole delays the onset of ventilator-dependence or tracheostomy. It is sold under several trade names, including Rilutek (tablet), Tiglutik (oral suspension), and Exservan or Emylif (an oral film). These drugs may cause several adverse effects, such as dizziness, gastrointestinal issues, and liver complications.

Edaravone (Radicava) is a potent pyrazolone free radical scavenger and antioxidant that represents a new addition to the restricted array of current disease-modifying therapies for ALS (Ali and Baker 2017). Edaravone may slow functional decline in daily activities. It is administered intravenously or orally in liquid form. The possible side effects include bruising, headaches, and difficulty walking. Symptom management and therapies require a multidisciplinary approach involving physical therapy, respiratory therapy, speech therapy, nutritional support, and other specific therapies.

### Amyotrophic lateral sclerosis and antioxidants

Antioxidants such as vitamins E, C, and B have been investigated for their potential in managing oxidative stress conditions in ALS, although the findings are inconsistent (Wang et al. 2019). Some studies suggest that vitamin E may be effective in reducing the risk and progression of ALS. In contrast, deficiencies in B vitamins (B2, B9, and B12) can produce symptoms similar to those of neurological disorders. Furthermore, the overall antioxidant status among patients varies, underscoring a complex relationship that necessitates further epidemiological trials.

### Vitamin E

Vitamin E is characterized by its lipophilic nature and is one of the most frequently studied antioxidants because of its ability to cross the cell membrane and provide protection against the peroxidation of lipids. This property facilitates its dissolution in lipids, thereby affecting its absorption and transport within the body through lipoproteins (Carrera-Juliá et al. 2020). Furthermore, it plays an essential role in safeguarding fatty cell membranes against oxidative damage.

Erythrocyte activity measurements of three primary ROS-scavenging enzymes, Cu, Zn-SOD, catalase, and glutathione peroxidase, in patients diagnosed with sporadic and familial ALS revealed significantly lower values than those in healthy subjects (Przedborski et al. 1996). The administration of vitamin E (and vitamin C and beta-carotene) did not influence the activities of any of the three enzymes.

A group of 289 patients diagnosed with ALS for less than 5 years were randomly assigned to receive either daily doses of alpha-tocopherol or a placebo for a duration of one year (Desnuelle et al. 2001). Biomarkers of oxidative stress and a modified Norris limb scale were used to evaluate the rate of functional deterioration. Like in the previously discussed study, after 12 months of treatment, alpha-tocopherol did not significantly affect the primary outcome measure and did not affect survival rates. Alpha-tocopherol was able to reduce the likelihood of progressing from milder state A to more severe state B on the ALS health scale. Three months of treatment resulted in increased glutathione peroxidase activity in the plasma and reduced plasma levels of thiobarbituric acid reactive species (TBARS) in the cohort receiving alpha-tocopherol alongside traditional medication.

A search through the Cochrane Neuromuscular Disease Group trials register, MEDLINE, EMBASE, and other sources (all up to 2003) was performed to evaluate randomized or quasirandomized controlled trials of antioxidant treatment for amyotrophic lateral sclerosis (Orrell et al. 2004). No significant effects were noted from the administration of vitamin E (500 mg, twice daily), acetylcysteine (50 mg/kg subcutaneously, daily), or a combination of L-methionine (2 g), vitamin E (400 IU), and selenium (3 × 10^− 5^ g, three times daily). A meta-analysis of antioxidants in general revealed no significant effect on the primary outcome measure when the results were aggregated. Additionally, no significant differences were found in the secondary outcome measures. There is inadequate evidence supporting the efficacy of individual antioxidants or antioxidants as a whole in the treatment of individuals with amyotrophic lateral sclerosis.

A case‒control study involving 132 patients and 220 healthy controls was performed to evaluate the dietary intake of vitamin E (Veldink et al. 2007). A significant association was found between a high intake of polyunsaturated fatty acids (PUFAs) and vitamin E and a reduced risk of developing ALS. The effects of PUFAs and vitamin E appeared to be synergistic, as evidenced by a combined analysis that revealed a further reduction in vitamin E and PUFA, along with a significant interaction term.

### Carotenoids

Carotenoids counteract oxidative stress by neutralizing ROS, including singlet oxygen (^1^O_2_), thereby safeguarding cells from harm. However, in cancer cells characterized by elevated ROS levels, carotenoids may paradoxically function as pro-oxidants, leading to cell death and illustrating their dual role (Ribeiro et al. 2018). In addition to their antioxidant properties, carotenoids are advantageous in conditions such as cardiovascular diseases and eye disorders because of their ability to quench ROS and facilitate electron transport.

The ALS Multicenter Cohort Study across 16 ALS clinics in the USA involving 302 patients with ALS revealed a beneficial effect of carotene consumption on ALS (Nieves et al. 2016).

This study aimed to examine the hypothesis that consuming fruits, which are rich in antioxidant nutrients, is inversely associated with the risk of developing ALS (Jin et al. 2014). A total of 77 patients diagnosed with ALS, along with an equal number of healthy controls, were assessed via a standardized food frequency questionnaire. The findings from multivariate logistic regression analysis revealed that fruit consumption was inversely related to the risk of ALS. In contrast, the intake of beef, fish, and fast food was positively correlated with the risk of ALS. Furthermore, the risk of ALS was negatively correlated with the consumption of plant-based calcium and beta-carotene but positively correlated with the intake of total calcium and animal-derived calcium. Taken together, the consumption of fruits and beta-carotene may reduce the risk of sporadic ALS.

### Polyphenols

Stilbenes are a group of natural polyphenolic compounds found in plants and characterized by a 1,2-diphenylethylene nucleus. Resveratrol is a naturally occurring stilbenoid polyphenol that functions as a plant-derived antioxidant and has anti-inflammatory, antiaging, and cardiovascular protective effects. Resveratrol exists in both cis and trans isomeric forms and is present in grapes, berries, and peanuts as a defense agent against injury or pathogens. The potential benefit of resveratrol in ALS is substantiated by the activation of Sirtuin 1 (SIRT1), which improves mitochondrial function and promotes the expression of several protective genes. Sirtuin 1 is crucial for regulating longevity, metabolism, and stress resistance. Treatment of the ALS cell model with resveratrol had a dose-dependent protective effect, substantiated by enhanced cell viability, prevention of apoptosis, and increased ATP production mediated by improved mitochondrial biogenesis (Wang et al. 2011). This study confirmed the protective role of resveratrol against mutant SOD1 in an ALS cell model through the upregulation of SIRT1 expression. Thus, SIRT1 can be considered a potential therapeutic target for preventing the pathogenesis of ALS.

The impact of resveratrol on SOD1(G93A) ALS mice has been studied (Mancuso et al. 2014). Upper and lower motoneuron functionality were assessed via electrophysiological evaluations, and the survival rates of the subjects and the spinal motoneuron population were quantified. Treatment with resveratrol postponed the onset of the disease and maintained motoneuron function in both female and male subjects. Furthermore, resveratrol notably increased the lifespan of SOD1(G93A) mice and supported the survival of spinal motoneurons. When resveratrol was administered at 12 weeks of age, the preservation and survival of spinal motoneuron function were enhanced. The protective effects of resveratrol increased the expression and activation of Sirtuin 1 and AMP-activated protein kinase (AMPK), which enhanced mitochondrial biogenesis in the spinal cord of SOD1(G93A) mice.

It has been reported that bone marrow-mesenchymal stem cells (BM-MSCs) derived from ALS patients exhibit functional impairments in the secretion of neurotrophic factors and display characteristics of cellular senescence (Yun et al. 2019). The activities of sirtuin 1/adenosine monophosphate-activated protein kinase (SIRT1/AMPK) were notably lower in ALS-MSCs than in bone marrow mesenchymal stem cells (BM-MSCs) from healthy controls. This reduction was ameliorated through pretreatment with resveratrol. The morphology of differentiated ALS-MSCs (ALS-dMSCs) included cell bodies and dendrites akin to those of neurons. Furthermore, compared with untreated ALS-dMSCs, resveratrol-treated ALS-MSCs significantly increased the differentiation rate.

Epigallocatechin gallate (EGCG) is a powerful polyphenol and the main catechin present in green tea; it is recognized for its significant antioxidant and anti-inflammatory properties. EGCG crosses the blood‒brain barrier and modulates mitochondrial responses to oxidative stress (Chung et al. 2010). The effects of EGCG on motoneurons subjected to oxidative stress were investigated (Koh et al. 2004). The viability of cells with the G93A mutation was lower than that of wild-type cells, which was attributed to a notable decrease in the activation of PI3K and the phosphorylation levels of Akt and GSK-3 in G93A mutant cells compared with those in wild-type hSOD1 4.1 cells. Compared with wild-type cells, exposure to hydrogen peroxide significantly reduced the viability of G93A mutant cells; pretreatment with EGCG before hydrogen peroxide exposure increased the viability of both cell lines. Biochemical analysis confirmed that the G93A mutation and oxidative stress led to a reduction in survival signals, including PI3K/Akt, while simultaneously increasing death signals such as GSK-3; the beneficial effect of pretreatment with EGCG was documented by increased survival signals and diminished death signals.

The neuroprotective properties of EGCG were studied in a SOD1-G93A transgenic mouse model of ALS (Xu et al. 2006). The findings of this study revealed that the oral administration of EGCG significantly postponed disease onset and prolonged lifespan. Moreover, EGCG-treated transgenic mice presented an increased number of motor neurons, reduced microglial activation, decreased immunohistochemical reactions for NF-kappa B and cleaved caspase-3, and decreased protein levels of iNOS and NF-kappaB in the spinal cord. Taken together, EGCG has multiple therapeutic effects on the ALS mouse model.

Curcumin is a polyphenol compound found in turmeric that is responsible for its bright yellow color and significant anti-inflammatory and antioxidant properties. It has neuroprotective properties and defends against oxidative stress, mitochondrial damage, inflammation, and protein aggregation (Ghasemi et al. 2019).

### Melatonin

Melatonin (N-acetyl-5-methoxytryptamine) is a hormone produced by the pineal gland that is capable of scavenging ROS (Reiter et al. 2016). Given its antioxidant properties, melatonin has been evaluated as a potential therapeutic agent for various neurodegenerative disorders associated with elevated ROS levels. In addition, melatonin promotes the expression of the most important antioxidant enzymes, such as SOD, GPx, and GR, as well as increasing GSH levels. Melatonin, by suppressing oxidative stress, can maintain mitochondrial homeostasis.

The antioxidant properties of melatonin in cultured motoneuronal cells (NSC-34) confirmed that melatonin reduced glutamate-induced cell death (Weishaupt et al. 2006). A genetic mouse model of ALS (SOD1(G93A)-transgenic mice) presented delayed disease progression and an increased survival rate. A clinical study of a cohort of 31 patients diagnosed with sporadic ALS revealed normalized values of oxidative stress markers following chronic high-dose (300 mg/day) rectal melatonin treatment. These series of results confirmed that high-dose melatonin is a promising candidate for further clinical trials of neuroprotection in ALS.

In contrast to previous studies, intraperitoneal melatonin supplementation in the G93A-SOD1 transgenic mouse model of familial ALS led to exacerbated neurodegeneration (Dardiotis et al. 2013). Melatonin markedly diminished animal survival rates, increased the loss of motoneurons (based on histological examination of the spinal cord), and enhanced the lipid peroxidation marker 4-hydroxynonenal. Western blot analysis of the spinal cord revealed upregulated expression of SOD1 in melatonin-treated subjects, which has been explained in the context of decreased survival rates by overcoming natural antioxidant and antiapoptotic mechanisms.

### ALS Conclusion

Numerous studies have provided substantial evidence regarding the involvement of oxidative stress (OS) mechanisms in amyotrophic lateral sclerosis (ALS), which leads to mitochondrial dysfunction and cellular damage, thereby contributing to neurodegeneration. While excessive reactive oxygen species (ROS) production is widely accepted as a prevalent pathological characteristic among ALS patients, uncertainty remains about whether oxidative damage serves as a primary cause or a secondary effect of the disease, as well as the actual role of OS in the progression of ALS, particularly when the various subtypes of patients are considered. Although the mechanisms underlying OS and mitochondrial dysfunction present promising avenues for therapeutic intervention aimed at decelerating disease progression, it is critically important to delineate the distinct OS profiles exhibited by different patient types (for example, identifying specific OS mechanisms linked to various mutations). This understanding is essential for the development of personalized therapies that can effectively slow the progression of the disease in accordance with the OS profiles of individual patients.

## Conclusions

Oxidative stress is a key factor in the progression of brain aging and neurodegenerative disorders. Dysfunctional mitochondrial respiration is associated with the breakdown of oxidative phosphorylation (OXPHOS), which is accompanied by the production of reactive oxygen species (ROS) during both aging and Alzheimer’s disease. Mitochondrial damage promotes the production of amyloid beta, which is a toxic stressor. ROS damage all biomolecules, including DNA, causing double-strand breaks and altering the epigenome. The disrupted homeostasis of redox metal ions (iron and copper) makes them inherently vulnerable to oxidative damage. This damage often precedes the clinical and pathological symptoms of Alzheimer’s disease, making it a critical focus of disease research.

Oxidative stress plays a central role in the development of Parkinson’s disease, as it directly damages and kills dopaminergic neurons in the brain structure called the substantia nigra. The brains of patients with Parkinson’s disease contain high levels of iron, which is a source of ROS via the Fenton reaction. As oxidative stress increases, energy (ATP) production decreases, which together damages cellular lipids, proteins, and DNA. Oxidative stress is amplified by the normal breakdown and autooxidation of dopamine, which produces toxic byproducts such as dopamine-derived quinones that contribute to the increase in oxidative stress.

In Huntington’s disease, mutant aggregates of huntingtin protein trigger oxidative stress by impairing mitochondria and reducing cellular antioxidant defenses. This redox imbalance causes a damaging cycle of reactive oxygen species (ROS) production, leading to DNA damage, protein dysfunction, and the loss of vulnerable neurons in the brain. In addition, an important cellular safeguard, the NRF2 pathway, is hindered by the mutated protein, preventing the brain from effectively clearing damaged molecules.

A crucial connection between amyotrophic lateral sclerosis (ALS) and oxidative stress is the mutation of the superoxide dismutase 1 (SOD1) gene. SOD1 functions as an antioxidant enzyme; however, when it is mutated, it misfolds and forfeits its typical protective role, thereby directly contributing to toxicity and the production of reactive oxygen species (ROS). Furthermore, excitotoxicity, characterized by excessive stimulation of neurons by glutamate, results in an influx of intracellular calcium, which further harms mitochondria and intensifies oxidative damage.

Antioxidant therapies to mitigate oxidative stress have various impacts on cognitive function in both elderly individuals and patients suffering from neurodegenerative disorders. There is a significant translational gap in the field of antioxidant therapy for neurodegenerative diseases, as encouraging preclinical findings frequently do not translate into cognitive or functional improvements in human clinical trials. Although oxidative stress is undoubtedly a major contributor to neuronal death, universal antioxidant supplementation encounters substantial biological, pharmacological, and logistical challenges. To achieve reliable advantages, it is essential to meticulously evaluate factors such as dosage, timing, combinations of antioxidants, and dietary considerations. Major efforts require overcoming blood‒brain barrier (BBB) restrictions, the low bioavailability of antioxidants due to their rapid degradation in the gut, and subtherapeutic concentrations of active substances at the biological target.

## Data Availability

No datasets were generated or analysed during the current study.
